# Solvent organization in the ultrahigh-resolution crystal structure of crambin at room temperature

**DOI:** 10.1107/S2052252524007784

**Published:** 2024-08-27

**Authors:** Julian C.-H. Chen, Miroslaw Gilski, Changsoo Chang, Dominika Borek, Gerd Rosenbaum, Alex Lavens, Zbyszek Otwinowski, Maciej Kubicki, Zbigniew Dauter, Mariusz Jaskolski, Andrzej Joachimiak

**Affiliations:** a Structural Biology Center, X-ray Science Division Argonne National Laboratory Lemont IL60439 USA; b Bioscience Division, Los Alamos National Laboratory, Los Alamos, NM87545, USA; c Department of Chemistry and Biochemistry University of Toledo 2801 W. Bancroft Street Toledo OH43606 USA; d Institute of Bioorganic Chemistry Polish Academy of Sciences PoznańPoland; e Department of Crystallography, Faculty of Chemistry Adam Mickiewicz University in Poznań Poznań Poland; f Department of Biophysics and Department of Biochemistry The University of Texas Southwestern Medical Center Dallas TXUSA; g Macromolecular Crystallography Laboratory National Cancer Institute at Frederick Frederick MD21702 USA; h Department of Biochemistry and Molecular Biology University of Chicago Chicago ILUSA; UCL, United Kingdom

**Keywords:** sub-atomic resolution, independent atom model, static disorder, dynamic disorder, solvent modeling, radiation damage, water circuits, crambin

## Abstract

Ultrahigh-resolution structures provide unprecedented details about protein dynamics, hydrogen bonding and solvent networks. The reported 0.70 Å, room-temperature crystal structure of crambin is the highest-resolution ambient-temperature structure of a protein achieved to date. Sufficient data were collected to enable unrestrained refinement of the protein and associated solvent networks using *SHELXL*. Dynamic solvent networks resulting from alternative side-chain conformations and shifts in water positions are revealed, demonstrating that polypeptide flexibility and formation of clathrate-type structures at hydro­phobic surfaces are the key features endowing crambin crystals with extraordinary diffraction power.

## Abstract 2

Using synchrotron radiation, diffraction data extending to 0.70 Å resolution were collected from crystals of the small protein crambin at room temperature (297 K), and the structure was refined with spherical-atom approximation to an *R* factor of 0.0591, revealing (i) protein regions with multiple conformations, (ii) extended water networks correlated with protein conformations and (iii) minimal radiation damage. The structure sets a standard for room-temperature refinement of macromolecular targets and provides accurate data for modeling protein–solvent interactions.

## 1. Introduction

Crambin is a small hydro­phobic storage protein of 46 residues (4.7 kDa) found in the embryonic tissue (cotyledons and hypocotyledons) of seeds from *Crambe abyssinica*, a relative of mustard and canola, commonly known as Abyssinian cabbage. Crambin belongs to a family of small proteins called thio­nins found only in higher plants and was reported to be membrane associated (VanEtten *et al.*, 1965 ▸; Lobb *et al.*, 1996 ▸; Teeter & Hendrickson, 1979 ▸). Thio­nins are widely distributed and play an important role in plant metabolism, growth and development. The specific function of thio­nins is not fully understood; some have antimicrobial activity and can suppress and kill a variety of plant pathogens, some have been shown to be cytotoxic to animal cells, including cancer cells, presumably acting as defensins by penetrating the cell membrane and making it permeable (Schrader-Fischer & Apel, 1994 ▸). Crambin shows structural homology to the membrane-active plant toxins puro­thio­nin and viscotoxin, but itself is not toxic (Stec *et al.*, 1995 ▸; Pal *et al.*, 2008 ▸; Teeter *et al.*, 1993 ▸; Hendrickson & Teeter, 1981 ▸). Its amino acid sequence contains no Gln, His, Lys, Met or Trp but is enriched in Cys and Pro residues. Six cysteines are paired into three di­sulfide bridges, and together with five proline residues endow the protein fold with extraordinary structural stability. Crambin purified from seeds exists as two isoforms that differ at two amino acid positions, Pro22/Leu25 and Ser22/Ile25, called the PL and SI forms, respectively (Teeter *et al.*, 1993 ▸). Because of its hydro­phobicity, crambin requires organic solvents (such as ethanol or acetone) for solubilization and extraction. Crambin crystallizes readily and forms the best-ordered macromolecular crystals known, which diffract X-rays to the highest sub-atomic resolution of any protein known to date (Teeter & Hendrickson, 1979 ▸; Schmidt *et al.*, 2011 ▸). For this reason, crambin has been used in numerous structural studies with single-crystal X-ray diffraction, neutron diffraction and solution NMR (Teeter & Hendrickson, 1979 ▸; Hendrickson & Teeter, 1981 ▸; Teeter *et al.*, 1993 ▸; Stec *et al.*, 1995 ▸; Yamano *et al.*, 1997 ▸; Lamzin *et al.*, 1999 ▸; Pal *et al.*, 2008 ▸; Schmidt *et al.*, 2011 ▸; Ahn *et al.*, 2006 ▸; Bonvin *et al.*, 1993 ▸; Chen *et al.*, 2012 ▸). The crystals of crambin have also been used as standards for a variety of crystallographic techniques, including sulfur anomalous phasing (Hendrickson & Teeter, 1981 ▸), and data collection and refinement at ultrahigh resolution (Schmidt *et al.*, 2011 ▸; Jelsch *et al.*, 2000 ▸).

### 1.1. Brief outline of our project

Prior to the shutdown of the Advanced Photon Source (APS) in April 2023 for the scheduled upgrade, a final sub-atomic resolution experiment was conducted at the 19-ID beamline of the Structural Biology Center (SBC), utilizing crystals of the small protein crambin. During the operational lifetime of 19-ID, the beamline has been used for measuring sub-atomic resolution data for many proteins (*e.g.* Howard *et al.*, 2004 ▸; Wang *et al.*, 2007 ▸; Rosenbaum *et al.*, 2015 ▸), all collected under cryogenic conditions. A large number of crambin crystals were prepared and their diffraction characterized initially at room temperature (RT) and later also under cryogenic conditions. The crystals diffracted at RT to better than 0.70 Å resolution, and to better than 0.40 Å under cryogenic conditions. A complete dataset was collected to establish the highest-resolution RT crystal structure of a protein. As of July 2024, the Protein Data Bank (PDB; Burley *et al.*, 2018 ▸) has 28 X-ray crystallographic entries with resolutions higher than 1.0 Å, where data were acquired at temperatures above 273 K (Table S1 of the supporting information). This sub-atomic resolution structure of a protein and its associated solvent sets a standard for RT independent atom model (IAM) and can serve as a reference for comparisons of structures determined under different cryogenic conditions.

As part of an ongoing effort to describe a macromolecule at true atomic resolution, we report here the structure of crambin at 0.70 Å, which is the highest-resolution protein structure determined at RT to date, with data and model quality approaching those of small-molecule crystals. The structure was determined with synchrotron radiation using 31 keV (0.40 Å) X-rays and refined with independent spherical-atom approximation to an *R* factor of 0.0591 using *SHELXL*, resulting in the best refined macromolecular structure at RT to date. The model of the ordered part of the protein structure was refined without stereochemical restraints, thus providing high-accuracy geometrical parameters that can be used to validate the existing restraint libraries (Engh & Huber, 1991 ▸, 2001 ▸) and indeed to define protein-based protein restraints. The structure revealed (i) a protein molecule with numerous multiple amino acid conformations; (ii) extended and complex water networks, with water positions correlated with protein conformations. The structure shows only minimal radiation damage as indicated by analysis of the electron density near the three di­sulfide bridges present in the structure. The main focus of this paper is on the solvent structure in this RT protein crystal, which is unperturbed by any flash-vitrification procedures.

## 2. Materials and methods

### 2.1. Isolation, purification and crystallization

Crambin was isolated from seeds of *Crambe abyssinica* using acetone extraction as reported previously (VanEtten *et al.*, 1965 ▸; Lobb *et al.*, 1996 ▸). Sitting-drop vapor-diffusion crystallizations were set up at 289 K using 30 mg ml^−1^ protein in 80%(*v*/*v*) ethanol:water solution, equilibrated against 59%(*v*/*v*) ethanol:water reservoir (Teeter & Hendrickson, 1979 ▸; Schmidt *et al.*, 2011 ▸).

### 2.2. Set-up of the synchrotron beamline for sub-atomic resolution data collection

The X-ray diffraction experiments were carried out at the SBC 19-ID beamline at the Advanced Photon Source, Argonne National Laboratory (Rosenbaum *et al.*, 2006 ▸).

#### 2.2.1. Determining the optimal photon energy for sub-atomic resolution data collection

As previous tests with crambin crystals had shown diffraction to 0.40 Å, the beamline was set up so that 0.35 Å resolution would be achievable on half-frames on the Pilatus3 6M detector, *i.e.* with the beam center close to one edge of the detector and 0.35 Å resolution spots close to the opposite edge (Rosenbaum *et al.*, 2015 ▸). The detective quantum efficiency (DQE) of the detector decreases steeply at higher photon energies which will increase the radiation dose versus recorded diffraction intensity. The aim is to keep the photon energy as low as possible considering the physical limits of the detector and the endstation instrumentation. For 19-ID and the Pilatus3 X 6M detector, the maximum scattering angle is 70° resulting in a minimum photon energy of 31 keV (0.40 Å wavelength). For data collection at RT, test exposures showed that full frames (*i.e.* aligning the detector center with the beam center) will record all diffraction spots to 0.45 Å resolution.

#### 2.2.2. Extending the energy range of the monochromator to 31 keV and handling of diffraction effects from multiple λ

Beamline 19-ID was designed for a maximum energy of 19 keV using the 111 reflection of a silicon monochromator crystal which also defined the minimum Bragg angle. Thus, 31 keV photon energy requires the use of a higher-order reflection. The only practically available option is using the 333 reflection. The setup for 31 keV followed the details reported earlier (Rosenbaum *et al.*, 2015 ▸) for a 30 keV setup. We have used the undulator gap setting and low-energy absorbing filters to reduce the 111 reflection (10.333 keV) intensity and carefully adjusted the mirror angle to reduce the 444 and 555 reflections. The 10.333 keV contamination, though below the detector energy threshold and, thus, not counted, adds to the radiation dose.

#### 2.2.3. Beam intensities and doses

The uncollimated beam size at the sample is 80 µm × 40 µm (FWHM *H* × *V*). With a low-energy absorbing filter of 0.75 mm aluminium inserted, the flux of the 31 keV component was 4.4 × 10^11^ photons s^−1^. The flux of the 10.333 keV component was 4.6 × 10^8^ photons s^−1^.

For RT data collection, with the beam-defining slits closed to 50 µm × 50 µm (50 µm × 40 µm on the sample), the flux of the 31 keV component on the sample was 2.4 × 10^11^ photons s^−1^ and the flux density was approximately 1.2 × 10^14^ photons s^−1^ mm^−2^. Doses were calculated using the *RADDOSE-3D* webserver (Bury *et al.*, 2018 ▸). For a 180° rotation dataset at 0.2 s exposure per 0.2° rotation, the average diffraction-weighted dose from the 31 keV photons was 0.22 MGy. The maximum dose at the rotation center was 1.6 MGy. The 10.333 keV photons added an average dose of less than 1%.

### 2.3. X-ray data collection

Large crystals were mounted in glass capillaries (Charles Supper Company) with a small droplet of mother liquor and tested for diffraction. The approximate size of the crystal used for data collection was 0.45 mm × 0.39 mm × 0.20 mm. The temperature (297 K) was measured at the crystal position with a thermocouple over a period of several hours. Diffraction images were recorded on the Pilatus3 X 6M detector from four different sections of one crystal separated by 60 µm (see the supporting information), with datasets RTs4 and RTs5 collected from the same sector. The sample-to-detector distance was set to 160 mm with the beam position set to the center of the detector. To obtain highly redundant data and reduce radiation damage, 180° of data were collected from each section of the crystal. The first and second sections were exposed to X-rays for 0.2 s per 0.2° to collect lower-resolution data. The third section was exposed for 1 s per 0.2°, and the fourth section was exposed for 5 s per 0.2° to collect the highest-resolution data. Data were processed and scaled with *HKL2000* (Otwinowski & Minor, 1997 ▸) with modifications to account for specific features of the experiment as described in Sections 2.4, 2.5 and 2.6. Briefly, each dataset was indexed with an additional macro applied (*weak level 4.0*) and then integrated with the same spot size (0.50) and spot background (0.60) parameters, with the spot elongation limit adjusted to 2.5 mm and profile fitting radius adjusted to 120 mm so that the highest-resolution reflections are not rejected. After integration, all datasets were scaled and merged with the additional macros ‘*radiation dose B b factor*’ and ‘*anisotropy removal 3. 2.*’ applied during scaling to model the scaling *B* factor increase across data so that zero-dose extrapolation can be performed.

### 2.4. Handling of detector absorption and reflection profile integration

The Pilatus3 X 6M detector at 19-ID has a 1 mm-thick silicon sensor. At 31 keV, the DQE is 0.20 (*i.e.* only ∼1/5 of the incident radiation produces recorded diffraction). Radiation damage is a concern as higher doses are required. This is partially offset for the weak diffraction spots at high scattering angles: at a 70° scattering angle the extended path through the sensor increases the DQE 2.3× to 0.55.

Detector absorption and spot profiles were obtained from diffraction patterns of a silicon crystal. At the resolution of interest, the spot elongation was 2.4 mm. During data processing, the radial spot elongation profile was applied as implemented in the *HKL2000* software package (Otwinowski & Minor, 1997 ▸) with the elongation limit set to 2.5 mm (*elongation limit* parameter in *HKL2000*).

### 2.5. Applicability of crystal absorption correction

Absorption depends on the wavelength, and at 31 keV (0.40 Å), directional differences in absorption in the crystal and the thin-wall glass capillary should be approximately 1–2%. The overall scale factor, which includes directional effects other than absorption, varied up to ±12% across the virtual absorption surface, which is small in comparison with typical macromolecular data acquired at ∼1 Å wavelength (Otwinowski *et al.*, 2003 ▸).

### 2.6. Detection of and correction for sample radiation damage

Exposing crystals to X-rays during data collection induces various types of radiolytic processes, which proceed in the irradiated crystal volume and modify the molecules building the crystal lattice reproducibly, but in a manner specific to a particular molecule in a particular crystal lattice (de la Mora *et al.*, 2020 ▸). Recombination between products and intermediates of radiolytic reactions not only modifies the molecules in the crystal lattice chemically but also generates molecular gasses that rearrange these molecules leading to expansion and/or contraction of the unit cell. These direct and indirect effects are modeled in *HKL2000* with a two-component model, where the scaling *B* factor describing intensity decay as a function of dose is used together with reflection-specific extrapolation procedures, adjusting intensities for radiation-induced specific changes. The fitting procedure with as many as one parameter per unique *hkl* requires stabilization that is accomplished by Tikhonov regularization (Tikhonov & Arsenin, 1977 ▸). The Tikhonov regularization coefficient, expressed as a fraction of native intensity, serves as a quantification of specific radiation damage. The physical model underlying this correction has been published by Borek *et al.* (2010 ▸, 2013 ▸). In this experiment, we merged datasets acquired from multiple sectors of the same crystal, each exhibiting varying levels of crystal lattice disorder. The mosaicity estimates obtained in post-refinement for the crystal lattice parts used in the experiment are as follows: dataset RTs2: 0.050–0.091; dataset RTs3: 0.047–0.074; dataset RTs4: 0.072–0.074; dataset RTs5: 0.070–0.195; dataset RTs7: 0.274–0.399. The volumes of the sectors were exposed unevenly since the beam size was smaller than the diameter of the crystal. Consequently, during crystal rotation, unexposed unit cells were entering the beam while some exposed unit cells were exiting the beam, despite good centering. Additionally, the high-resolution pass datasets were exposed five times longer per oscillation step than low-pass datasets. Such variable exposures and the mixing of exposed and unexposed states during rotation necessitated additional assumptions during scaling about the doses across different experiments. We used the increase in the scaling *B* factor as a proxy for dose, as we previously demonstrated that overall radiation damage, described by the scaling *B* factor, depends linearly on the dose, at least for the doses used in these experiments (Borek *et al.*, 2013 ▸). This approach allowed us to associate each observation with a dose proxy obtained from scaling and then use these observations together for extrapolation to zero dose. Zero-dose extrapolated data were used in the structure refinement, as described below, and to analyze patterns of specific radiation damage using radiation damage maps (Diederichs *et al.*, 2003 ▸; Borek *et al.*, 2007 ▸, 2010 ▸, 2013 ▸).

Section 2.2 presents direct measurements of flux and associated calculations of theoretical dose for the 90 µm-diameter cylinder of sample around the rotation axis. However, these and other similar calculations do not account for irregularly shaped crystals larger than the X-ray beam, in which unexposed parts of the crystal are introduced into the beam at different times during rotation, while exposed parts of the crystal may leave the beam temporarily or permanently. In such a case, the result of the structure solution (*i.e.* the electron density map) will represent the state averaged across all partially damaged states, with the merged data representing the state corresponding to much lower dose than theoretically calculated. That effect is accounted for in all scaling procedures where the scale factor and the scaling *B* factor are used to model both overall radiation damage and the crystal volume changing in the beam. In the scaling procedures of *HKL2000* and *HKL3000*, one unit of scaling *B* factor increase corresponds to 1 MGy at 100 K (Borek *et al.*, 2007 ▸). However, at RT that correspondence has not been calibrated in a crystal-specific manner. We observed a scaling *B* factor increase of ∼0.93 Å^2^ for all four datasets merged together. In the past, we observed a ∼30-fold increase of the scaling *B* factor for the same dose used at two different temperatures, 80 K and 200 K, with exponential increase between these two temperatures (Borek *et al.*, 2007 ▸). However, at RT, there is no expectation that different systems will follow the same exponential increase in the scaling *B* factor. With the increased temperature during data collection, radicals produced by X-rays can easily recombine. These recombination processes and their rates are driven by local reactions specific to the particular crystal lattice and crystallization conditions. Thus, for all crystals the *B* factor will increase exponentially, but for each of them the exponent value will be different. Therefore, we cannot estimate the dose accurately, but collectively, the size of the crystal, the pattern of specific radiation damage and the scaling *B* factor values indicate that the dose was likely to be significantly lower than 1 MGy and significantly lower than the theoretically simulated values described in Section 2.2. As we could not accurately estimate the dose without additional calibration experiments, we extrapolated the data to zero dose, which provided a benefit in the refinement and assured a consistent reference point in data analysis.

### 2.7. Refinement protocol

The RT monoclinic *P*2_1_ crystal structure of crambin was initially refined using using PDB entry 3nir as the starting model with the program suite *Phenix* (Adams *et al.*, 2010 ▸). This initial model was rebuilt with *Coot* (Emsley & Cowtan, 2004 ▸) and *Phenix* refinement continued with data to 1.0 Å resolution. Because of the ultrahigh resolution (0.70 Å) and high quality of the diffraction data, the refinement was ultimately carried out with *SHELXL* (Sheldrick, 2015 ▸) without an extra *R*_free_ validation. After a few cycles of isotropic refinement of the preliminary model, the initial electron density maps were generated and thoroughly inspected in *Coot*. The maps clearly showed the positions of all protein atoms and well defined water molecules. For further refinement, standard stereochemical restraints for each amino acid residue, generated by the *SHELXPRO* (Sheldrick, 2015 ▸) program using the Engh & Huber (1991 ▸, 2001 ▸) dictionary, were included. After each round of 20 cycles of conjugate-gradient least-squares (CGLS) minimization, the program *Coot* was used for visualization of the electron density maps and for manual adjustment of the atomic model. The full resolution was used from the very beginning of the *SHELXL* refinement. The atomic scattering for *SHELXL* calculations was adjusted for the short wavelength (0.39995 Å) by providing DISP instructions with proper dispersion coefficients for the relevant elements.

After the initial stage of refinement, the geometrical restraints were globally relaxed for all protein residues. Subsequently, the weights of the restraints were gradually correlated with the degree of disorder of the individual amino acid residues. The restraints were individually tightened for specific residues showing disorder, especially in the fragments corresponding to the PL and SI isoforms of the protein, and at residues in more than two conformations (Table S2). The effective standard deviations of the restraints for bond lengths and angles of the disordered residues were adjusted in subsequent refinement steps. Ultimately, all geometric restraints for ordered protein fragments were removed, leaving in place only restraints for disordered residues with weights adjusted to the degree of disorder. We consider an atom to be disordered if its occupancy refines to a value lower than 1.0. In some cases, other distinct conformations of that atom can be modeled, and occupancies of these distinct states will add up to 1.0, indicating static disorder. However, in other cases, modeling with a number of distinct conformations is not sufficient to achieve full occupancy, indicating dynamic disorder. Because the structure contains a mixture of two (partially disordered) isoforms (PL and SI) and residues with triple conformations, the occupancy factors of the individual atoms in such residues must be treated in a special way. For Val8 and Tyr29, the occupancy factors of each conformation (the same for all atoms of a given conformer) were refined as three free variables [FVAR instruction of *SHELXL* (Sheldrick, 2015 ▸)], constrained to sum up to 1.0. However, for residues at positions 22 and 25, additional restrictions are necessary: the sum of occupancies of fractional conformations combined with the fractions of the two isoforms for each atom should be equal to unity. For the double conformations of Ser22A/B and Ile25A/B of the SI isoform, the occupancies refined to 0.310 (16)/0.332 (16) and 0.354 (18)/0.288 (18), respectively. This means that the total occupancy of the SI form is 0.642 (16) (0.310 + 0.332 or 0.354 + 0.288, with rounding precision), while in the single conformation PL isoform, the Pro22 and Leu25 residues are present with an occupancy of 0.358 (8). This fraction, together with that for the SI isoform, gives the total occupancy of one. We note that in this crystal of crambin the proportion of the PL and SI forms is different (35.8% and 64.2%, respectively) than reported previously (60% and 40%, respectively) (Teeter *et al.*, 1993 ▸). Additionally, the occupancy coefficients of the three discrete Tyr29 conformations were coupled to the occupancies of residues 22 and 25 through a common free variable for the occupancy of one of the Tyr29 conformations and the occupancy of Pro22. Each of the occupancy factors mentioned above was refined as a separate free variable, and the sum of all occupancies for each position was additionally constrained to 1.0 using an appropriate SUMP instruction. The SUMP instruction of *SHELXL* allows us to set a linear relationship among free variables and is mostly used to constrain the occupancy factors of more than two atoms sharing the same site, or of three or more complementary conformations. Twelve residues of the protein (Thr1, Thr2, Ile7, Arg10, Phe13, Pro19, Ser/Pro22, Ile/Leu25, Ile34, Gly37, Thr39 and Asp43) are present in double conformation, and two (Val8 and Tyr29) in triple conformation (Fig. S1 and Table S2 of the supporting information).

Water molecules were included in the model manually based on the difference electron density maps (*mF*_o_ − *DF*_c_) and stereochemical considerations. All occupancy factors of disordered protein and solvent atoms (except hydrogen atoms) were refined. Most of the water molecules (60 out of 73 sites) were refined to partial occupancy. The occupancy of any water molecule that was close to unity was fixed at 1.0. The electron density maps indicated the presence of two partially occupied molecules of ethanol from the crystallization buffer, which were refined as well.

Hydrogen atoms of the protein molecule were added and refined at riding positions. The *SHELXL* AFIX 87 instruction was used to refine the hydrogen positions of the hydroxyl groups. Hydrogen atoms, for which there was clear difference electron density, were added to 15 water molecules. There was no attempt to model the hydrogen atoms of the ethanol molecules. All hydrogen atoms were treated isotropically.

At the final stage of the refinement, one cycle of full-matrix least-squares (L.S.) minimization was calculated with the DAMP 0 0 instruction (‘ignore the corrections’) and all the restraints removed, for the purpose of estimating the standard uncertainties in all individual refined parameters and all derived geometrical parameters.

The number of reflections per parameter in the final refinement (more than 9) matches the best situations encountered for non-centrosymmetric small-molecule structures. This allowed us to reduce the number of stereochemical restraints to the absolute indispensable minimum.

Considering the mobility, disorder or inhomogeneity of some of the crambin fragments, the residues that should be stereochemically restrained were selected manually (15 residues of the 46 total) and included all residues in multiple conformation and Pro41.

### 2.8. Residual bulk solvent visualization

To visualize bulk solvent contribution (*i.e.* the solvent that has not been explained with the atomic model), we calculated maps with and without bulk solvent correction applied. The operation was carried out using *REFMAC* (Murshudov *et al.*, 1997 ▸), where we selected ‘simple’ scaling with or without the ‘calculate the contribution from the solvent region’ option, and ran the program with zero cycles of refinement. The resulting two .mtz files were visualized in *Coot* (Emsley & Cowtan, 2004 ▸), and *Coot* tools were used to calculate the difference between the two maps (*F*_c_, φ_c_) by applying a scale factor of −1 to the map obtained without bulk solvent correction.

## 3. Results and discussion

### 3.1. Quality of the diffraction data

The structure is of high quality with data collected from four segments of one large crystal scaled together, providing excellent coverage and redundancy at low (14.47 Å, reflection 110) and high resolution (0.70 Å). We nearly doubled (1.75×) the number of observations compared with previous RT structures of crambin (PDB entries 1crn and 3u7t) with excellent crystallographic statistics [*R*_merge_ (0.048), mean *I*/σ(86.4), CC_1/2_ (1.00), completeness (98.2%), redundancy (13.7) and Wilson *B* factor (1.14 Å^2^), see Table 1 ▸]. These statistics approach those observed in small-molecule X-ray crystallography.

### 3.2. Overall quality of the model

**Figure 1:**
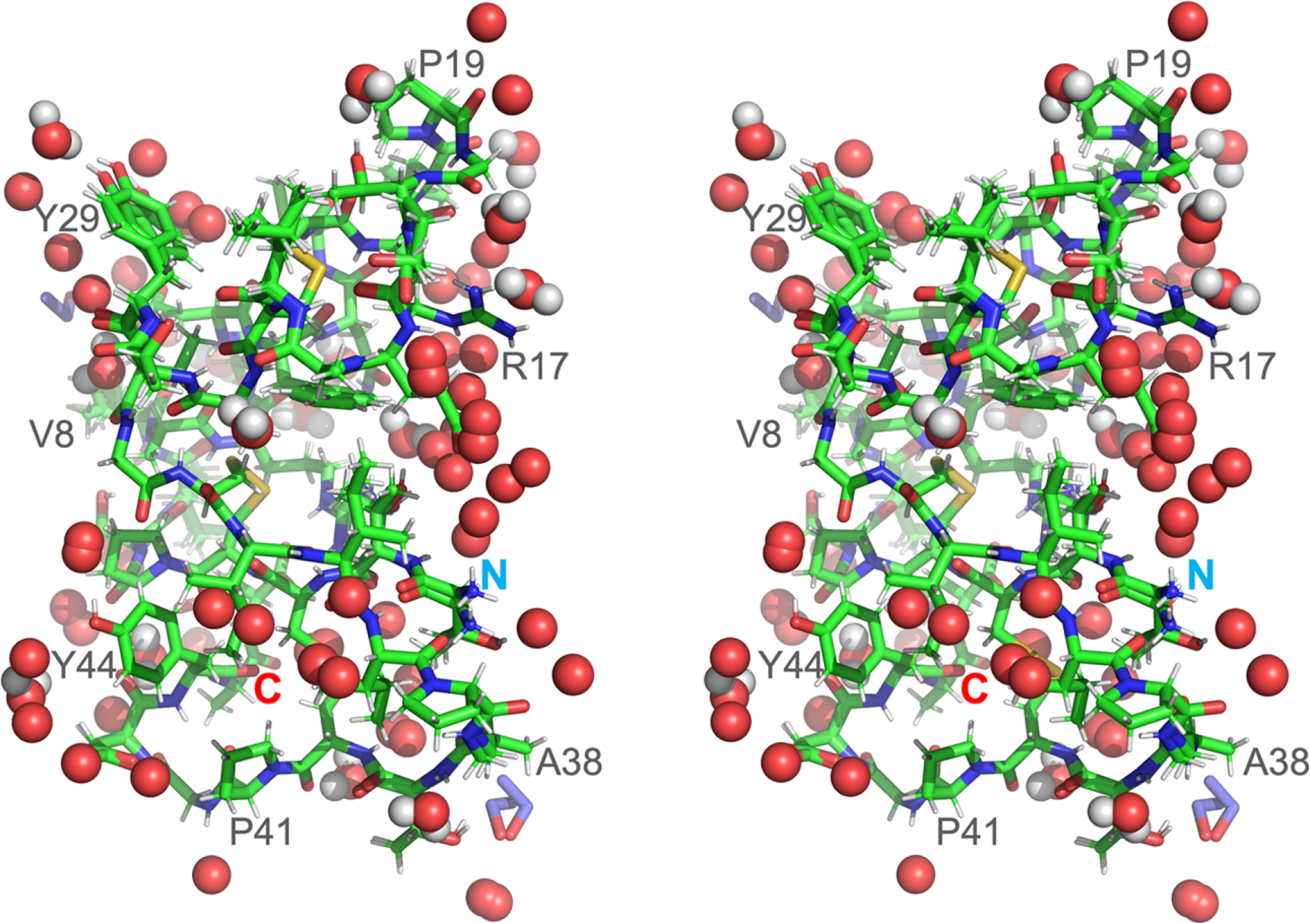
RT structure of crambin at 0.70 Å resolution with solvent bound in stereo representation [protein is represented by sticks: green (C), blue (N), red (O), yellow (S), silver (H); water molecules are red spheres with hydrogen atoms in silver and ethanol molecules are blue/red sticks]. N- and C-termini as well as some residues are labeled.

**Figure 2:**
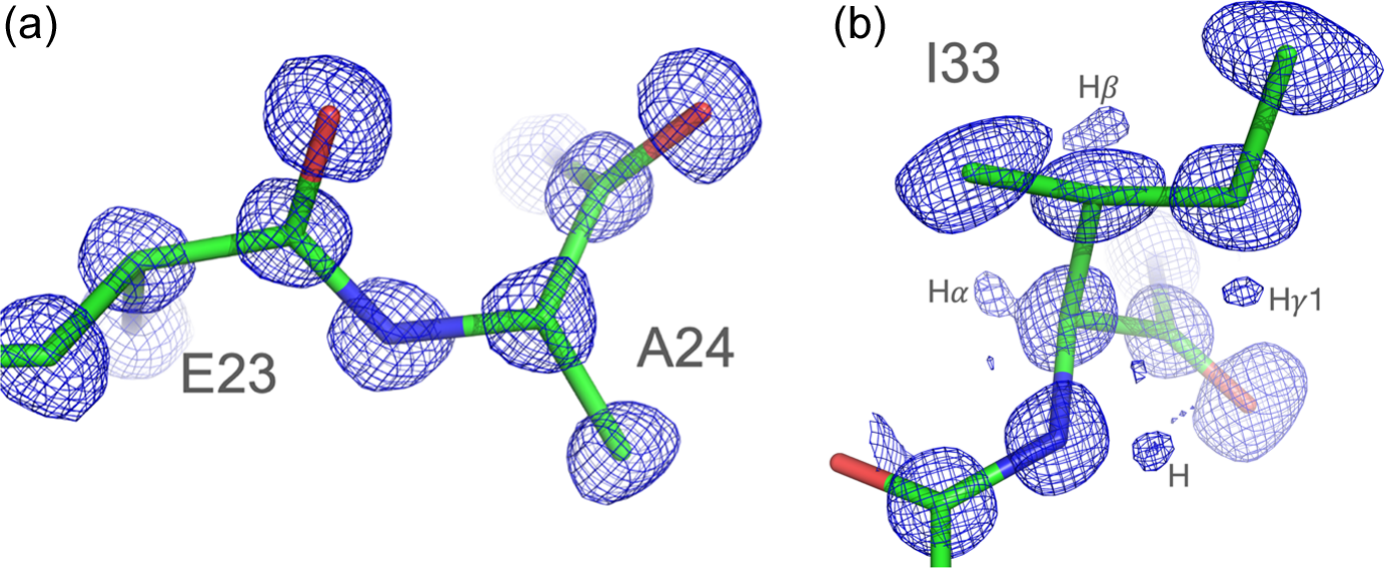
2*mF*_o_ − *DF*_c_ electron density map of protein regions. (*a*) Glu23–Ala24 peptide electron density contoured at the 1.2σ level. Note that the electron density peaks are approximately proportional to the number of electrons of their atoms. (*b*) Electron density map (*mF*_o_ − *DF*_c_; contour level 1.1σ) around Ile33. Ile33 is in the hydro­phobic core and is very well ordered. Hydrogen atom electron densities for H, Hα, Hβ, Hγ1 are very visible.

Our structure of crambin (Fig. 1 ▸) refined to the crystallographic *R* factors of 0.0591 (4σ*F*_o_ cutoff) and 0.0759 (no σ cutoff) is of the highest quality compared with the previously reported ambient-temperature crystal structures of the mixed form of crambin available in the PDB (PDB entry 1crn; Teeter, 1984 ▸), and a more recent structure of H/D exchanged crambin (PDB entry 3u7t; Chen *et al.*, 2012 ▸). The electron density of the protein and solvent region is outstanding (Figs. 2 ▸, 3 ▸ and S1). A comparison of the three RT structures shows that the crambin structures are very similar. Using the jFATCAT algorithm in the pairwise structure alignment tool at https://www.rcsb.org, the main-chain root-mean-square deviation (RMSD) values are 0.10 Å and 0.11 Å against 3u7t and 1crn, respectively. The main-chain RMSD between 3u7t and 1crn is 0.07 Å. The largest differences between our RT structure and those reported previously are in improving the interpretation of multiple protein conformations, partially occupied solvent sites and describing overlapping continuous networks of solvent structure in intermolecular regions, typically termed static disorder, but here interpreted as alternative solvent networks.

The refined protein molecule is similar to other models of crambin deposited in the PDB, but the structure reported here stands out for the achieved accuracy of the refined parameters. The standard uncertainty (s.u.) values of the fully occupied atomic positions (0.00008–0.0005 Å, except for sulfur atoms, where they are about 0.00003 Å), calculated by the inversion of the LS matrix, are very close to values typical for crystal structures of small organic compounds. The s.u. values of the coordinates are inversely proportional to the atomic number of the refined atoms and correlate with their atomic displacement parameters (ADPs) as expected (Fig. S2).

The estimated standard deviation (e.s.d.) values characterizing C—C bond distances, commonly used as a global indicator of the quality of organic small-molecule structures, are very low and have an average value of 0.016 Å (0.006 Å for ordered atoms). For comparison, the average σ(C—C) of 0.005 Å is a threshold for the most precise organic crystal structures in the Cambridge Structural Database (CSD; Groom *et al.*, 2016 ▸). In well defined regions of the present crambin model, the e.s.d. values for the carbonyl bonds of the ordered main chain range from 0.003 Å to 0.009 Å, with a mean of 0.004 Å. This indicates that the present structure can be classified in the group of the most precise structures, taking into account also small-molecule structures.

When comparing the final protein model with the ideal stereochemical geometry (Engh & Huber, 1991 ▸, 2001 ▸), very low RMSDs for bond lengths (0.016 Å) and bond angles (1.77°) were obtained, even though the major part of the protein was refined without stereochemical restraints.

All bond lengths and angles of the main and side chains, together with their calculated values of standard uncertainties, are included in an Excel spreadsheet in the supporting information, in a fashion often followed for small-molecule structures. Additionally, all the values listed are compared with the standard reference parameters and their standard deviations (σEH) tabulated by Engh & Huber (2001 ▸), which in lower-resolution refinements are used as restraints for protein covalent geometry. The values of bond lengths and angles of the present crambin structure agree well with the ideal geometry, even though our refinement was essentially restraint-free. The difference of bond lengths is <1σEH for ∼90% of the data. Only two cases of side-chain bond lengths exceed 2σEH. About 80% of the valence angle values are within 1σEH. Only two side-chain bond angles differ by more than 3σEH from the targets.

The refinement was performed using zero-dose extrapolated intensities and so the model represents the state without or with minimal radiation damage. However, the analysis of radiation damage maps calculated from non-extrapolated data indicates that the radiation damage was either minimal or that extensive recombination, possible at RT through diffusion, was able to ‘heal’ the damaged parts. The highest radiation damage peaks are present at the di­sulfide bridges. However, there was no sign of de­carboxyl­ation. Minimal damage also indicates that the data collection protocol, which consisted of two ‘low-dose’ passes in different sections of the crystal, followed by one medium dose pass and a longer exposure dataset to collect the high-resolution data, was successful in managing and minimizing radiation damage.

The radiation damage maps were calculated using the procedure described in Section 2.6. For each unique reflection, a Tikhonov-regularized line was fitted. The slope of this line serves as the difference map coefficient, representing the difference between the most damaged and the zero-dose states. The analysis of this map shows that the four strongest negative and the four strongest positive peaks were located at the three di­sulfide bridges, namely −23.73σ (−0.57 e^−^ Å^−3^) and +7.97σ (+0.19 e^−^ Å^−3^) peaks located at Cys32 (Cys4–Cys32 di­sulfide bridge), −15.52σ (−0.37 e^−^ Å^−3^) and +11.88σ (+0.29 e^−^ Å^−3^) peaks located at Cys26 (Cys16–Cys26 di­sulfide bridge), −12.60σ (−0.30 e^−^ Å^−3^) and +7.76σ (+0.19 e^−^ Å^−3^) peaks located at Cys3 (Cys3–Cys40 di­sulfide bridge), and −11.37σ (0.27 e^−^ Å^−3^) and +11.10σ (+0.27 e^−^ Å^−3^) peaks located at Cys16. Overall, in the asymmetric unit (ASU) there were 86 negative and 9 positive peaks exceeding the ±5σ level.

Crambin is lacking His, Met, Lys, Trp and Gln and the reduced diversity of its sequence might be an additional contributor to the relatively low level of specific radiation-induced changes we observed, although the data collection protocol, low cumulative dose and RT were likely the dominant factors. In general, di­sulfide bridges, Asp, Glu and Met are the most strongly affected by specific radiation-induced changes in X-ray diffraction experiments. For His, Lys, Trp and Gln little to no changes are usually observed. In our case, with a dose below 1 MGy, we observed radiation damage localized at all three di­sulfide bridges, as expected, and the Cys residues were damaged to varying extents, which is also expected as the local environment is either able to accommodate Cys residues in different conformation after di­sulfide bridge disruption, or it is too constricted to accommodate such changes, or else it facilitated more frequent recombination back to a conformation similar to the original (Petrova *et al.*, 2010 ▸). We did not observe any damage to Pro residues or water molecules. This is also expected because overall radiation damage in macromolecular structures does not significantly depend on amino acid composition. All amino acids contain similar elements (C, N, O, S) that, on average – after correcting for the absorption cross-section – sustain damage in a similar manner. However, radiation damage depends on the temperature, as the mobility of radicals causing secondary events is much higher at RT. Thus, while overall radiation damage is not amino acid-specific, it progresses much faster at RT. All scaling programs correct for overall radiation damage, so its impact is removed from the diffraction data.

Specific radiation damage describes deviations from the overall radiation damage. It is relatively small, typically representing 0.5–2% of the signal change per 1 Å^2^ of the scaling *B* factor increase owing to overall radiation damage. We do not expect these small deviations to change significantly with temperature. In other words, while overall damage will occur faster at RT, specific radiation damage will still constitute a similar fraction of the overall radiation damage. Additionally, specific radiation damage may be lower at RT for some systems because the increased mobility of radicals allows recombination reactions to occur more easily, compared with cryotemperatures where limited diffusion for species heavier than OH groups prevents recombination.

In our case, a dose of less than 1 MGy results in at most 2% of the change in structure factors meaning that, even for extremely accurate data, we will observe minimal specific radiation damage. The number of peaks crossing the threshold of ±5σ is consistent with these expectations.

As an additional proof of the quality, the model was checked using the checkcif procedure for small-molecule structure validation (Spek, 2020 ▸). The model passed this scrutiny quite well, considering the degree of disorder and the size of the system (reported in the supporting information). The only serious problems reported are related to the shapes (eccentricity) of thermal ellipsoids for atoms in disordered and solvent areas. Other alerts are of relatively lower importance (*e.g.* an isolated oxygen atom; a *D*—H bond without an acceptor; short H⋯H contacts). It might be hoped that with lowering the temperature of the crystal, the structure will approach small molecule standards.. At 0.70 Å resolution, the data:parameter ratio of 9:1 allows for anisotropic modeling of non-hydrogen atom vibrations (ADPs). The introduction of anisotropic ADPs reduced the *R*/*R*_free_ factors from 0.1711/0.1788 to 0.1165/0.1275.

### 3.3. Regions with multiple conformations

**Figure 3:**
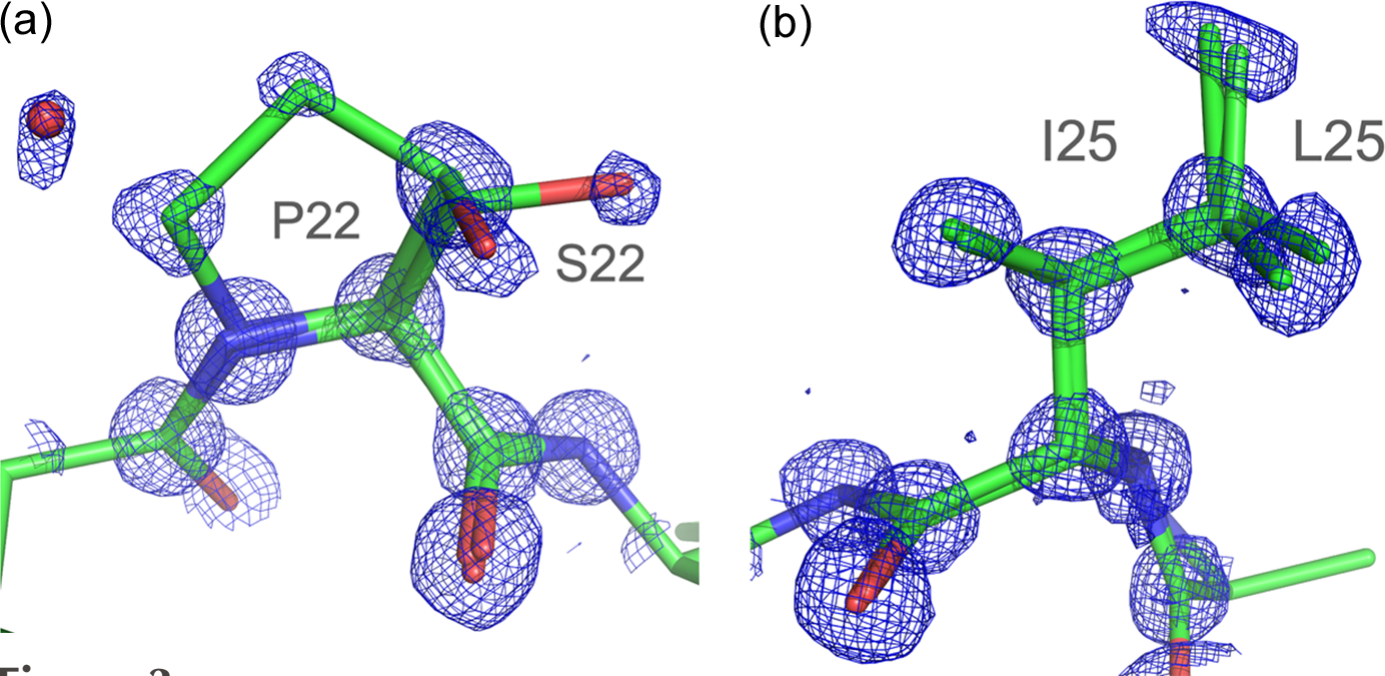
2*mF*_o_ − *DF*_c_ electron density map around residues Pro/Ser22 and Ile/Leu25. (*a*) Dual sequence with triple conformation of residue 22. One proline and two serine conformers occupy the same location. All corresponding atoms have solid electron density at the 1.2σ level. W46A, close to the proline side chain, also has partial occupancy. (*b*) Ile/Leu25 dual sequence with multiple conformations. Single conformer for Leu and two for Ile occupy the same location (contour level 1.3σ).

The protein crystallizes as a mixture of isoforms differing at two sequence positions, and in addition there are several residues that were modeled in multiple conformations. Residue Ser22, part of the two distinct sequences present within the crystal, has its side chain in two conformations (Fig. 3 ▸), which in turn influences the nearby solvent structure as well as interactions with symmetry-related molecules. While there is crystal-to-crystal variation in the relative amount of the two different isoforms, the structure presented here, determined from a single crystal, refines to approximately 64.2 (16)% SI form and 35.8 (8)% PL form. Aside from occupancy refinement, one way to determine this distribution crystallographically is to examine the region of crystal contacts between symmetry-related molecules and their associated solvent networks. The side chain of residue Tyr29 adopts three different orientations, with two related orientations present at 22.7 (23)% (B) and 41.5 (23)% (C) occupancy, separated by 0.7 Å and interacting with Thr30 and Cys16; and the third orientation at 35.8 (8)% (A) [Fig. S1(*b*)]. Conformation A is moving closer to Thr30 and is significantly shifted from the first two (B and C) by 1.9 Å to accommodate a van der Waals interaction with an adjacent, symmetry-related Pro22. The more common conformations (B and C) would be in steric clash if Pro22 were present; therefore, we conclude that these conformations are only consistent with a crystal contact with Ser22. This means that Tyr29 exists in one conformation together with the PL isoform, while in the presence of the SI isoform, it occupies two other alternative positions (Fig. S3).

The proportion of the PL and SI isoforms in natural preparations of crambin deserves a special note here. In our crystal structure the PL/SI ratio is 0.358/0.642, arrived at a convergent LS refinement and accompanied by a standard uncertainty of ∼0.01. Therefore, it is very puzzling that, in almost all previous crystal structure determinations of crambin purified from native sources, an inverse PL/SI ratio was reported, with the PL form being systematically more abundant. For example, the PL/SI ratio is 0.60/0.40 in 1cbn (Teeter *et al.*, 1993 ▸) and 0.57/0.43 in 1ejg (Jelsch *et al.*, 2000 ▸). In a series of crambin structures (PDB entries 1jxt, 1jxw, 1jxx and 1jxy) determined by Teeter *et al.* (2001 ▸) at different temperatures, the Pro:Ser ratio at position 22 is 0.55:0.45 but the Leu:Ile ratio at position 25 is 0.50:50, even though Pro is in one sequence with Leu, and Ser with Ile. An exception in this series is 1jxu, where the PL/SI ratio is 0.60:0.40. A similar inconsistency is found in structure 3u7t (Chen *et al.*, 2012 ▸), where the Pro:Ser22 ratio is 0.47:0.53 while the Leu:Ile25 ratio is 0.39:0.61. In 3nir (Schmidt *et al.*, 2011 ▸), which is currently the highest-resolution (0.48 Å) structure of crambin, where atoms in the same residue are listed with different occupancies, for example, the atoms of Ser22 appear with occupancies between 0.28–0.36. We do not have a simple explanation for these strange observations, other than the assumption that natural crambin might crystallize with different (but concrete) proportions of the isoforms. On the other hand, our X-ray diffraction experiments with crystals of crambin grown from entirely different protein preparations (not shown) still agree with the PL:SI proportion (0.358:0.642) found in the present work. A test run of full-matrix LS refinement with the occupancy factors of the PL and SI forms defined as independent free variables with a 0.5:0.5 ratio, converged with practically the same occupancy ratio as under proper restraints (and with the estimated standard uncertainties of the occupancies of 0.01–0.02), additionally confirming the validity of the isoform proportion reported in this work. A mass spectrometry spectrum recorded for crystals (identical to those used for the X-ray diffraction experiments) dissolved in ethanol confirmed that the lower molecular weight form SI was roughly twice in abundance relative to the higher-molecular-weight form PL, in agreement with the results of our crystallographic refinement. This, together with the often-inversed ratio reported in previous papers, makes this issue very mysterious, indeed.

In the final model, 14 residues of the total 46 were modeled with more than one conformation, with conformational variability primarily involving side-chain rotamers (Table S2). One residue, Gly37, has two main chain conformations. These multiple conformations cluster around a crystal contact region that involves Tyr29 and three adjacent symmetry-related molecules. Tyr29 is in contact with the regions of alternative sequences, as described above, and in addition makes contacts with hydro­phobic residues (Ile7 and Val15) from a symmetry-related molecule, which show different side-chain conformations. Tyr29 also contacts a symmetry-related Leu18, which is in a single conformation. We suggest that this flexibility is important for the formation of a tightly packed and highly stable crystal, through accommodation of different side-chain conformations and an associated rearrangement of nearby solvent atoms. Residues in the hydro­phobic core are very well ordered and show electron density for hydrogen atoms, as in the example provided by Ile33 (Figs. 2 ▸ and S3).

### 3.4. Hydrogen-bonded networks coupling the protein and solvent regions

#### 3.4.1. Dense packing of crambin molecules in the crystal

There is one crambin molecule in the ASU and 33.6% solvent by volume. The ordered part of solvent consists of water molecules (see detailed discussion below), and only two molecules (one partial) of ethanol (sufficiently ordered to allow modeling). The packing of the crambin molecules is very tight and 12 symmetry-related protein molecules interact with the central molecule, via direct or water-mediated contacts (Fig. S4). The coordination polyhedron defined by the crambin molecules is a distorted (elongated) cuboctahedron and is quite different from the closest packing of spheres (Fig. S4), resulting in a different contribution of symmetry-related protein and solvent molecules to the interactions.

Crambin is a hydro­phobic protein and in fact there are a number (12) of hydro­phobic side chains exposed on the surface. As a result, only 32.4% of the crambin surface is polar. The hydro­phobic side chains are engaged in contacts with symmetry-related hydro­phobic mates [for example, Ile7 interacts with Ile33′(*x*, *y* + 1, *z*), Val8 interacts with Leu18′′(*x* + 1, *y*, *z*), Val15/Leu18 interact with Ile25′(*x*, *y* + 1, *z*), *etc*.].

**Figure 4:**
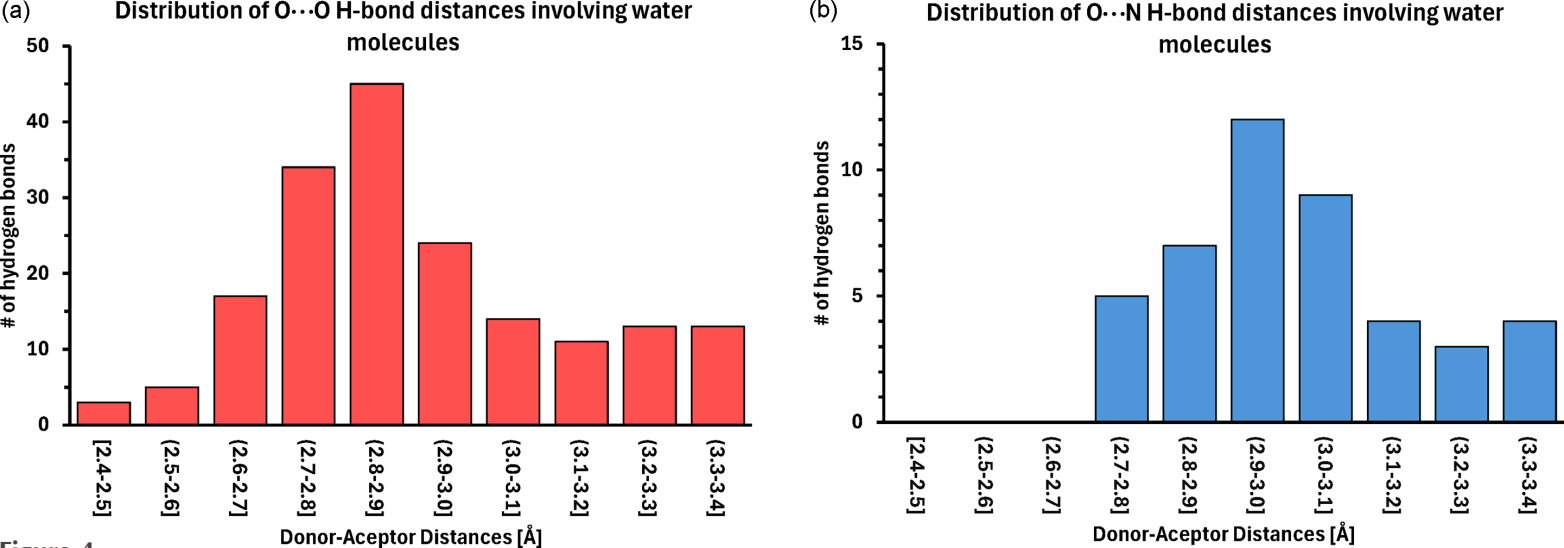
Distribution of H-bond distances [(*a*) O—H⋯O and (*b*) O—H⋯N] in the crystal of crambin at RT.

Water molecules form extensive networks of hydrogen-bond interactions with protein molecules and other water molecules. The water hydrogen-bond distances (in O, N, S and π interactions) range from 2.42 Å to 3.40 Å, with the majority (74.9%) within 2.62–3.10 Å, and an average hydrogen-bond distance of 2.93 Å (Fig. 4 ▸). The distances are slightly shorter in O—H⋯O bonds (average 2.91 Å) than in O—H⋯N bonds (3.02 Å), as expected from the difference (0.1 Å) between the van der Waals radii of the N and O atoms (Pauling, 1969 ▸).

Water molecules formally assigned to the principal ASU are only part of the ordered solvent story because extensive interactions exist between symmetry-related molecules. By including these molecules in the analysis, we can obtain a complete picture of the solvent structure. The water molecules can be divided into several categories. Some have direct hydrogen bonds to protein side-chain or main-chain atoms. There are also water molecules interacting with solvent only and there are a number of molecules with partial occupancy that exist in two or three alternative positions separated by less than 1 Å. These sites cannot be occupied at the same time.

The ethanol molecules interact more with the water network than with the protein hydro­phobic side chains. One ethanol molecule exists in double conformations, and it also has hydrogen-bonded water molecules showing similar behavior near its OH group.

There are several water molecules with extensive hydrogen-bond contacts. W22 has 5 possible interactions, 14 water molecules (8, 10, 11, 12, 20, 21, 26, 30, 32, 38, 41, 42, 45, 48) have 2 hydrogen-bond donor and 2 acceptor interactions. In the crystal, we observe contiguous solvent chains composed of more than 10 water molecules. Some are linear, some form circuits, and some branch out and connect to other water chains.

#### 3.4.2. Water hydrogen atoms

Out of the 73 water sites in the ASU, assignment of (isotropic and riding) hydrogen atoms based on difference (*mF*_o_ − *DF*_c_) peaks was possible for as many as 15 water molecules (1, 2, 3, 4, 5, 6, 7, 9, 12, 13, 14, 15, 17, 19, 20). Here we analyze the O—H⋯*X* interactions of these hydrogen atoms.

The H2 atom of W1 is directed towards the ring centroid (CM) of Tyr44 from an *x*-translated crambin molecule, thus forming an O—H⋯π hydrogen bond (H⋯CM 2.88 Å, O⋯CM 3.69 Å). The second hydrogen atom of W1 (H1) is engaged in hydrogen bonding to W5. Atom H1 of W5 is bridging it to W8. No hydrogen atoms were found at W8. The second hydrogen atom of W5 (H2) bridges this water molecule to the carbonyl group of Gly20.

W1 is an acceptor of the O—H1 donor from water W7. H2 of W7 forms a link to W27, which has no hydrogen atoms modeled. W27 accepts, however, another O—H1 group from W20. The O—H2 group of W20 could be forming a hydrogen-bond to the side-chain oxygen atom of Tyr29. However, the automatic refinement of the O—H orientation of this (disordered) Tyr29 residue is not compatible with such a possibility.

The hydrogen atoms of W2 link it to W11 (no hydrogen atoms present) and to the carbonyl group of Leu18. The W2 and W20 molecules are within hydrogen-bonding distance but, again, the disposition of the hydrogen atoms of W20 is incompatible with such a bond. Atom H1 of W20 is, however, properly oriented for hydrogen bond formation with W11.

The hydrogen atoms of W3 are utilized for bridging two carbonyl groups of the protein, Pro5 and Asp43. W4 functions as a similar bridge (Ala24, Ser11′) but between two symmetry-related protein molecules.

The hydrogen atoms of W6 could link this molecule with W51A and the hydroxyl group of Thr21, but also in this case the automatic placement of the Thr21 O—H group precludes such a scenario. W9 is hydrogen bonded to the carbonyl group of Cys40 and uses its second hydrogen atom in an H-bridge with W33.

W12 is a hydrogen-bond donor to the hydroxyl group of Ser11 and to W18 and is in turn an acceptor of the O—H2 donor from W14. The second O—H group of W14 is a donor to W4. The two hydrogen atoms of W13 are directed towards naked oxygen atoms of W26 and W73. The hydrogen atoms of W15 anchor it to the carbonyl group of the Asn14 side chain and to W57. Likewise, W17 bridges the carbonyl group of Cys32 and W69. W17 is an acceptor from the O—H group of Ser11.

W19 is peculiar because its O—H groups are not directed toward any proper hydrogen-bond acceptors. On the other hand, the oxygen atom of W19 is within (weak) hydrogen-bond distances of the carbonyl group of Thr30 and the side-chain NH_2_ group of Asn12.

The neutron structure of crambin (PDB entry 4fc1) provides a detailed model of hydrogen atom positions in the protein as well as in solvent regions (Chen *et al.*, 2012 ▸). A comparison with the X-ray structure presented here shows that the solvent network is largely identical, with minor variation in the modeled hydrogen atom positions of solvent molecules, when available. The X-ray structure, owing to the high resolution, is more completely modeled in terms of multiple conformations of the polypeptide chain as well as the number of solvent molecules (73 versus 42 in the neutron structure). However, several features of the neutron structure need to be noted. Due to the strong scattering of neutrons by hydrogen atoms (as H or D), nearly all hydrogen atoms could be modeled in the protein as well as the solvent region, effectively doubling the number of refined parameters. The neutron structure, though reported at what could be considered ultrahigh resolution (1.10 Å), still necessitated careful refinement to avoid overfitting of the experimental data. As a result, a conservative final model was reported comprised of only the major (PL) isoform of the protein and a limited number of solvent molecules. Anisotropic ADPs were used for protein non-hydrogen atoms and a small number of well ordered D atoms. Nevertheless, for first-shell solvent molecules, the nuclear density for the D atoms was very clear, with unambiguous assignment of hydrogen-bond donors and acceptors. Taken together, the neutron and X-ray structures are complementary. Neutron crystallography was able to experimentally resolve nearly all hydrogen atoms in the polypeptide chain and provided accurate hydrogen bonding information for the well ordered solvent molecules, while the X-ray structure was able to reveal more features of the solvent region and more dynamic features within the crambin polypeptide.

### 3.5. Dissection of the water structure

The solvent structure shows an overlapping continuous network of water molecules in the intermolecular region, spanning the space between twelve symmetry-related molecules. Despite crystallization conditions containing a 59% ethanol:41% water (*v*/*v*) mixture as the precipitating agent, only two ethanol molecules are resolved in the structure, showing signs of partial occupancy or disorder. The solvent structure also highlights protein sequence-dependent networks. Crystals of crambin grow most readily and diffract best as a mixed form. In our crystals of crambin the proportion of the PL and SI forms is different (35.8% and 64.2%, respectively) than those reported previously (60–50% and 40–30%, respectively) (Teeter *et al.*, 1993 ▸, 2001 ▸; Jelsch *et al.*, 2000 ▸; Chen *et al.*, 2012 ▸; Schmidt *et al.*, 2011 ▸). This difference is mysterious, but it may be due to different protein preparation procedures or small changes in the crystallization conditions, or even different refinement protocols. The changes in the alternative side chains lead to local perturbations of the solvent network around these residues. The sequence variations enable alternative solvent networks, resulting in more stable crystal packing and lower energy of interactions, thus improving crystal stability and X-ray diffraction power.

With the PL and SI isoforms of crambin present in the crystal structure in the (refined) proportion of 64.2 (16)/35.8 (8)%, the molecular mass of the protein is 4726.9 Da, yielding a Matthews volume of 1.853 Å^3^ Da^−1^ and the corresponding solvent fraction of the crystal of 0.336. From this fraction one can estimate that the ASU should contain ∼98 molecules of water, assuming that the specific density of liquid water is 1 g cm^−3^ and that there is no distinction between the protein hydration layer and bulk solvent. The number of water sites modeled in this RT structure is 73 (*i.e.* 75.5% of this expected number). However, since many (60) of these water sites have fractional occupancy, the real experimentally determined water content (*i.e.* the sum of all occupancies) is 49.14. This would correspond to modeling of about 50.1% of the solvent structure. However, we also have 1.814 molecules of ethanol in the model, which makes the modeled water fractions more optimistic. Assuming the specific density of liquid ethanol to be 0.79 g cm^−3^, the volume occupied by the ethanol molecules in the ASU is estimated at 175.4 Å3, and the remaining volume would be occupied by ∼92 water molecules. Relative to this total number of water molecules in the ASU, the number of modeled sites (73) would amount to 79.3%, and the total sum of water occupancies (49.14) to 53.4%.

We note here that in the early paper describing an isomorphous crystal structure of crambin at 0.945 Å resolution, Teeter (1984 ▸) estimated the number of water molecules in the asymmetric unit at 85.

Among the 73 water sites per ASU in the present RT crambin structure, 46 are in direct hydrogen-bonding contact with only one protein molecule (there may be other water-mediated contacts, but we do not count them in this inventory). Nine and four water molecules bridge together two protein molecules related by *x*- and *z*-translations, respectively. Five bridging water molecules sit between 2_1_-related protein molecules. Five water molecules have good electron density but no hydrogen-bond contacts within 3.4 Å.

There are many hydrogen bonds interlinking the hydration sphere into a complex network. Most of the chains of hydrogen-bonded water molecules are traced as not very long, just a few links, but we have to remember that there are also circuits and chains involving protein atoms.

**Figure 5:**
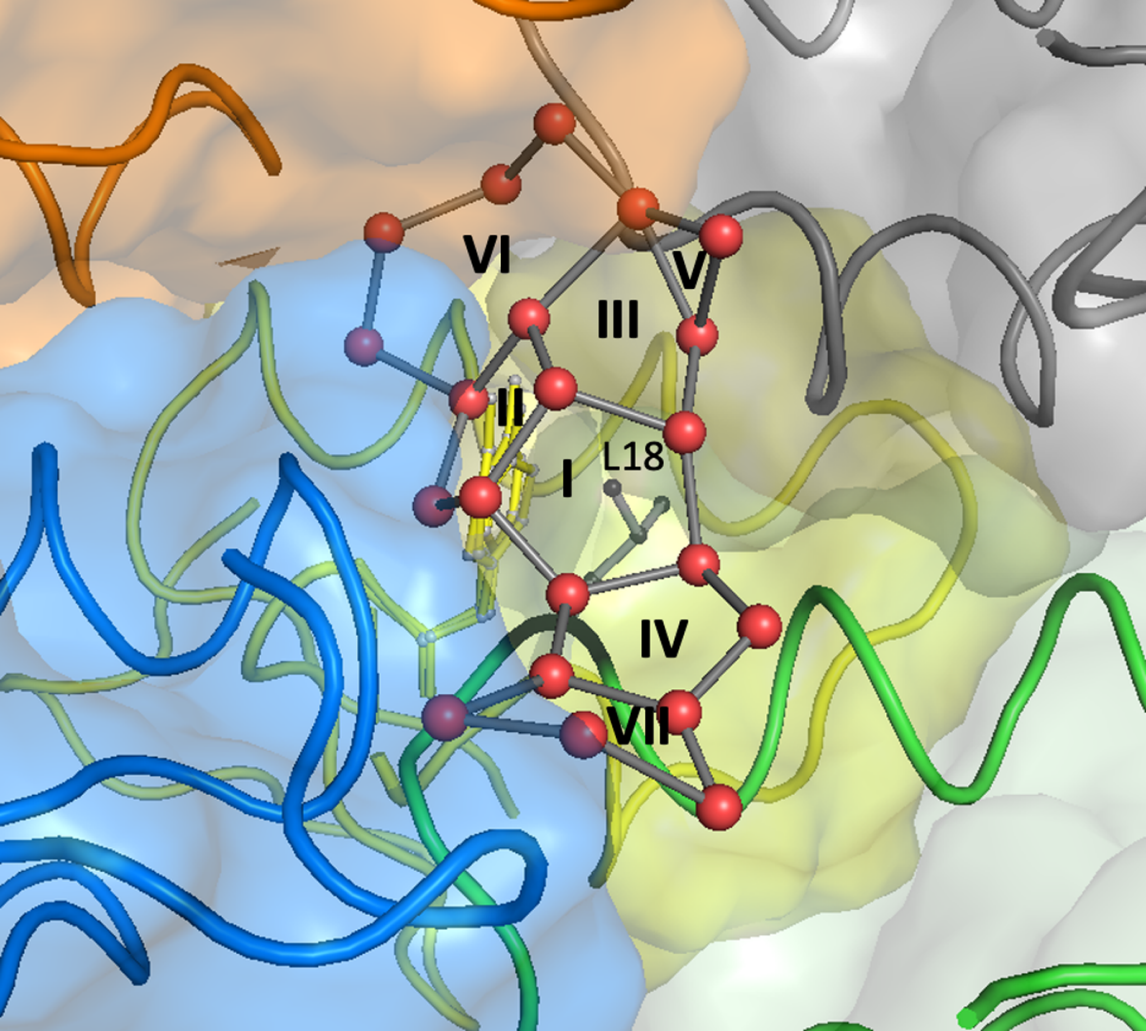
Network of water rings at the hydrophobic interface between symmetry-related protein molecules shown in different colors in ribbon and surface representations. Water molecules are shown as red spheres connected by grey sticks, representing hydrogen bonds. The Leu18 side chain is presented as a grey ball and stick model.

The most prominent patterns that can be discerned in this network are water pentagons, formed at the hydro­phobic interface between several symmetry-related protein molecules. In her paper reporting the crambin structure at 0.945 Å resolution, Teeter (1984 ▸) describes five such circuits, A–E, near Leu18, joined pairwise by common edges. In our structure, we see the same four central pentagonal rings (A, C, D, E in Teeter’s nomenclature), but the rest of this system is different (Fig. 5 ▸). To avoid confusion, we will label our rings using Roman numerals with a subscript designating the ring size. The most important is a cone-shaped motif formed by rings I_5_ (C), II_5_ (A) and III_5_ (E), which have a common tri-valent vertex and share pair-wise edges. This 13-node water cap is a perfect shield of the aliphatic side chain of Leu18, which lies directly below ring I_5_ (Fig. 5 ▸).

Ring IV_5_ (D) shares one edge (of the two free nodes) with ring I_5_ (C), and is further extended, again by edge fusion, to ring VII_5_, which fades to bulk solvent, as some its nodes already have low occupancies. On the other end, ring III_5_ is extended by one triangular motif V_3_ (with two O⋯O contacts to the apex), which is formed in our structure instead of the B ring described by Teeter (1984 ▸). Finally, an extended water heptagon, VI_7_, is fused to rings II_5_ and III_5_ with a common tri-valent vertex. Together with the adjacent system of pentagons, the seven-membered ring covers an area that is a void partially occupied by the threefold disordered Tyr29 residue and the juxtaposed system formed by the sequential diversity of Pro/Ser25, with the additional twofold disorder of the Ser residue [Figs. 4 ▸ and S1(*b*)].

It appears that the system of the fused water rings, with particular importance of the seven-membered circuit, plays an essential role in isolating a volume in this crystal packing, where the sequential variability of crambin (at least at residue 25) can be safely accommodated, allowing in addition for its correlation with the variability of the only residue displaying a significant level of disorder (Tyr29) in this structure.

Assuming that the central Leu18 residue in this area is from the principal copy of the protein in the ASU, the surrounding crambin molecules creating this ‘hydro­phobic hub’ in the crystal structure are generated by the *x*-, *y*- and diagonal *x*/*y*-translations, and by the 2_1_ screw axes of the space group.

### 3.6. Alternative water circuits and water clusters and alternative protein conformations

A number of water molecules surround the protein and occupy alternative sites. At least 16 water molecules (8, 11, 16, 18, 22, 26, 29, 30, 32, 33, 44, 46, 49, 51, 53, 57) exist in two or sometimes three partially occupied positions that cannot be occupied at one time because these molecules would be too close to each other. These molecules have partial occupancies that sometimes add to full occupancy, for example W8 (0.63 and 0.37), W11 (0.66 and 0.34), W18 (0.62 and 0.38), W51 (0.75 and 0.25) and W57 (0.43, 0.32, 0.26), but in some cases they add up to less than 1.0, for example W29 (0.51 and 0.30) or W49 (0.36 and 0.43). These water molecules most often cluster together near side chains of residues that exist in multiple conformations. The classic example in crambin is Tyr44, which has seven surrounding water molecules at double/triple positions (W3, W8, W22, W16′, W29′, W53′, W57′). Interestingly, this water network also includes molecules with very well defined positions (W7, W27) that refine at full or near-full occupancy. There are other regions at the crambin surface that contain clusters of water molecules with double positions. For instance, a region near Thr2, Arg10 and Glu23 has multiple water sites (W16, W29, W30, W52, W57 and W22′), and Ser6 also has several such associated water molecules.

Since the water molecules also interact with symmetry-related crambin molecules and respond to conformational changes of their side chains, the crystal should be considered as a complex network of interactions between protein molecules, solvent and other components (ethanol). Through partially occupied, correlated sites, these networks of interactions seem to switch from one state to the other and there may be many such states in this crystal of crambin. Because of the atomic resolution and sophisticated refinement protocols, we can visualize these networks and explain their nature. These observations suggest that there are distinct alternative states associated with the solvent structure. Because of the high resolution of our experimental data, the well defined positions of these solvent molecules may be refined with high confidence. It will be interesting to compare the water structure in crambin crystals at RT and at very low temperature, achieved by flash vitrification (work in progress).

**Figure 6:**
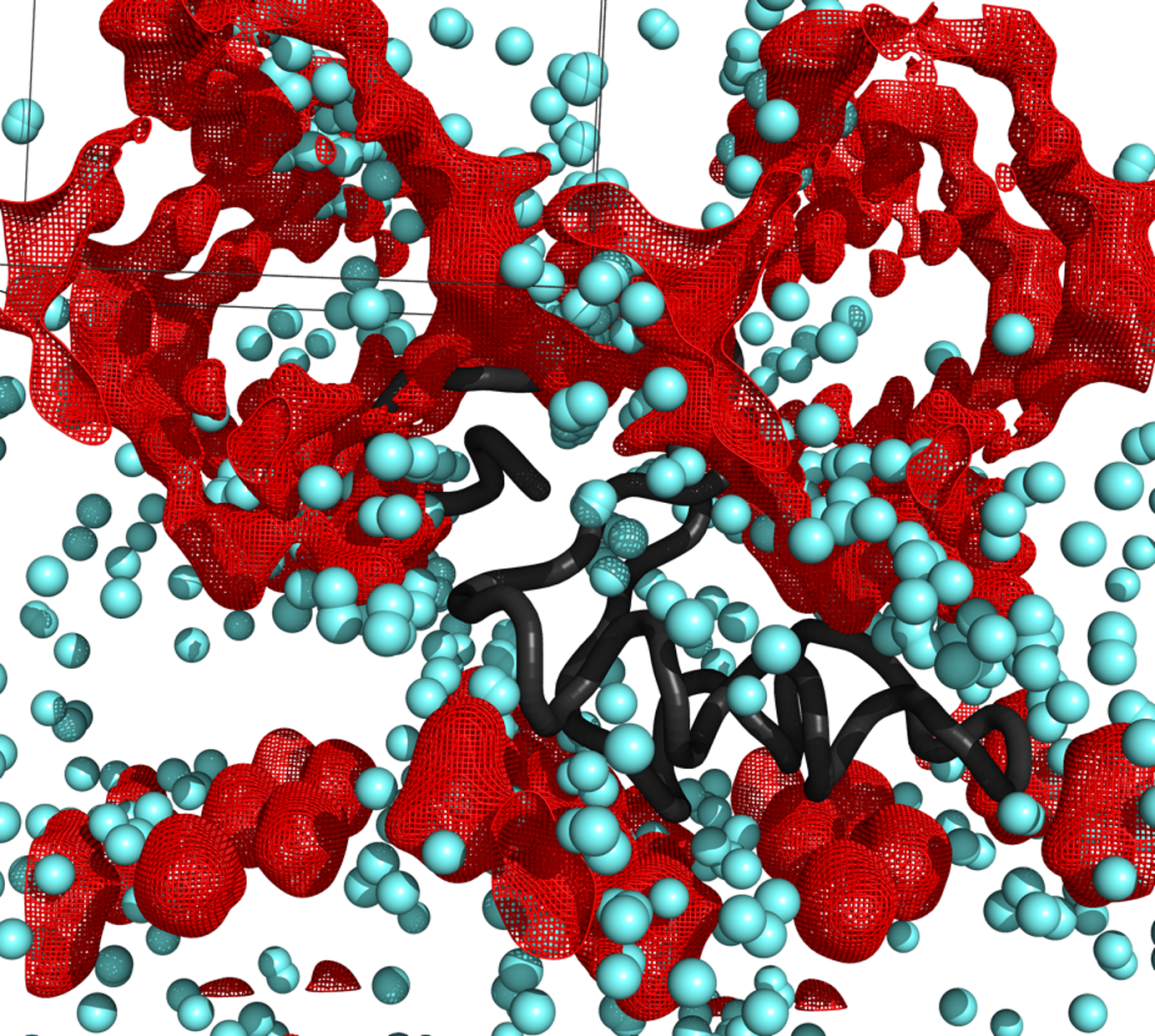
Bulk solvent around the molecule of crambin. Ordered water molecules are blue spheres, bulk solvent channels are shown as red mesh. Symmetry-related crambin molecules (occupying the empty spaces) are not shown.

Water is a solvent with unusual properties, and the features of its organization on macromolecular surfaces remain the subject of intense debate (Mondal *et al.*, 2017 ▸; Mondal & Bagchi, 2022 ▸). Even in the tightly packed crambin crystal lattice determined here at RT to 0.70 Å resolution, organized water constitutes only 79.3% of the expected total solvent content, with the remaining solvent described by the bulk solvent model (Fig. 6 ▸). We attribute this level of solvent disorder to the dynamic exchange of solvent molecules on the surface of the protein, an exchange not constrained by cryo-cooling, and to the increased number of conformations away from the protein surface, for which the residual electron density could not be modeled. It has been reported that water molecules interacting with crambin can exchange quite rapidly (Chen *et al.*, 2012 ▸; Ahn *et al.*, 2006 ▸; Bonvin *et al.*, 1993 ▸). Therefore, the solvent networks system visualized in our structure represents a collection of dynamic states where water molecules from crystal channels exchange with bound ‘ordered’ waters.

### 3.7. Detection and refinement of mobile hydrogen atoms in O/N—H groups and in water molecules

Of the 73 modeled water molecules in the structure, 15 have hydrogen atoms visible in the *mF*_o_ − *DF*_c_ density maps, generally corresponding to highly ordered waters in the first shell of hydration and those involved in the polygonal networks described above.

### 3.8. C/N—H⋯π hydrogen bonds

Two hydrogen bonds were observed between delocalized electron systems and water molecules, with W1 forming an interaction with the aromatic ring of Tyr44, and W22 forming an interaction with the delocalized electron system at the guanidinyl group of Arg17. These interactions were also observed in the high-resolution neutron structure of crambin (Chen *et al.*, 2012 ▸).

### 3.9. The N- and C-termini

The N-terminus of the protein, composed of Thr1 and Thr2, is modeled in alternative conformations reflecting the lack of crystal contacts and the expected flexibility of the amino terminus. The side chain of Thr1 is influenced by the nearby Gly37, which has been modeled in two alternative main-chain conformations. This is in contrast to the C-terminal Asn46, where the carboxyl­ate terminal is involved in a salt bridge interaction with the positively charged guanidinyl group of Arg10.

## 4. Determination of dataset resolution; comparison of our resolution standard to the nominal maximal resolution

Our excellent-quality data were acquired with high multiplicity and processed in a manner that minimized the impact of systematic effects, including radiation damage. Our decision to select 0.70 Å resolution as the cutoff in model refinement was driven by consideration of the interplay between properties of the data and uncertainties contributing to the accuracy of the refinement. In crystallography, even the best refined models have some level of error, while the experimental errors are estimated as uncertainty of the intensity. In macromolecular structures, at lower resolution the model errors are larger than experimental errors and at higher resolution the converse is true (Borek *et al.*, 2003 ▸; Holton *et al.*, 2014 ▸). The weight of these contributions to the refinement for each reflection index is *w_hkl_* = 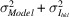. These two weights are generally calculated at the level of structure factors amplitudes. For every refinement process, we will have a resolution range, in which the model error dominates, and so each reflection index contributes about the same amount of information to the refinement process in this range. At resolutions higher than the bounds of this range, the experimental errors start to dominate over the model errors and conversely the contribution of reflection indices exceeding this resolution to the refinement becomes smaller. The point at which the experimental error starts to dominate over model errors is project dependent. In macromolecular crystallography, the typical *R* factors of 15% to 25% correspond to uncertainty of intensities between 30% to 50%, so the resolution limits defined by correlations between halves of data or *I*/σ(*I*) ≃ 1 are appropriate. However, in our case we reached an *R* factor of ∼6%, thus we applied a stricter criterion of *I*/σ(*I*) ≃ 2 and debated even harsher cutoffs, but decided against it as the refinement remained stable with the addition of data from higher-resolution shells. Also, the measurable diffraction extended beyond 0.70 Å, but was slightly anisotropic, which suggested that some reflections in the last resolution shells would carry more information than others and so were worth keeping in the data analysis. Scaling and merging indicated that diffraction intensities in the best direction were on average stronger than *I*/σ(*I*) ≃ 2, while in the worst direction, *I*/σ(*I*) ≃ 2 was reached at approximately 0.72 Å. We selected the nominal resolution of 0.70 Å for data analysis. Although we indexed and integrated 54 039 possible diffraction peaks, the ellipsoidal truncation applied to correct this residual anisotropy resulted in the rejection of 6928 reflections from resolution shells between 0.73 Å and 0.70 Å during merging.

## 5. Conclusions and outlook

### 5.1. Summary of this work and its place in the wider context of ultrahigh-resolution studies of crambin at different temperatures

Ultrahigh-resolution structures provide a great depth of detail about protein structure, dynamics, interactions, hydrogen bonding and solvent networks. In this 0.70 Å RT study of the crystal structure of crambin we focused our attention on the water structure rather than on the protein itself. It is a rather unusual possibility to have a view of the hydration structure of a protein crystal in its natural RT state, rather than at some roughly determined temperature of flash-vitrification. This seemingly modest increase in resolution, from 0.86 Å in the H/D exchanged crambin X-ray structure 3u7t (Chen *et al.*, 2012 ▸) to 0.70 Å reported here, nearly doubled (1.75×) the number of experimental observations compared with previous RT structures and increased by more than 50% (1.52) the number of observations used in the refinement. We improved the refinement protocols and show that it was possible to identify over 75% of the water sites, many of which are partially occupied and participate in correlated static disorder, also involving protein components. Remarkably, for 15 of the fully occupied water molecules we could model their hydrogen atoms in *mF*_o_ − *DF*_c_ electron density peaks, showing how far one can extend map interpretation at sub-atomic resolution, RT and with superb data quality.

Some of the most outstanding water patterns (pentagonal networks surrounding hydro­phobic islands) were partly observed previously by Teeter and colleagues. The present study (PDB entry 9ewk) should therefore be considered as the standard reference for crambin crystals at RT and ultrahigh resolution.

The protein was refined freely with *SHELXL*, down to *R* = 0.0759 [0.0591 for |*F*_o_| > 4σ(*F*_o_)], with geometrical restraints applied only to the disordered residues, including the two sequence positions (22 and 25) that have heterogenic amino acid compositions. The observed molecular geometry is, therefore, free of any prior bias. This will be very important, when ultimately, as it is hoped, protein geometry will be derived from proteins in the PDB, and not only from small-molecule analogs in the CSD. The accuracy and precision, the latter gauged by the estimated standard uncertainties calculated by full-matrix LS minimization, reach a level that is comparable with what is usually achievable in small-molecule crystallography.

Finally, we can validate our structural model not only using PDB tools, but also following the strict protocols used by small-molecule crystallography. The protein model in this analysis passes the tests well. However, the water structure requires special treatment because it is normally not encountered to such an extent in small-molecule crystals.

This sub-atomic resolution structure of protein and its associated solvent sets a standard for the RT atomic model and can serve as a reference for comparisons of structures determined under different cryogenic conditions. This will be the subject of a forthcoming report.

### 5.2. Is the solvent structure at RT supposed to be different from that trapped upon flash-cryo-cooling (of undetermined temperature *T* > *T*_target_)?

We note that during this data collection session, a dataset of ∼0.40 Å was also collected at 15 K, using helium-cooled crystals. Though the analysis and structure solution are still underway, it will be useful to compare structures resolved at ambient and helium temperatures. Parallel examples of this type are still very rare, as >90% of crystal structures reported in the PDB were determined under nitro­gen cooling conditions, at ∼90–100 K. In the present case, the extraordinary resolution will be used for a detailed evaluation of both the protein and the solvent structure in the crystal.

## Supplementary Material

All bond lengths and angles. DOI: 10.1107/S2052252524007784/ro5041sup1.txt

scl. DOI: 10.1107/S2052252524007784/ro5041sup2.txt

Water H-bonds list. DOI: 10.1107/S2052252524007784/ro5041sup3.txt

Main-side-chain-B. DOI: 10.1107/S2052252524007784/ro5041sup4.xlsx

RTs_2_00001.cbf. DOI: 10.1107/S2052252524007784/ro5041sup5.png

RTs_3_00001.cbf. DOI: 10.1107/S2052252524007784/ro5041sup6.png

RTs_3_00001.cbf. DOI: 10.1107/S2052252524007784/ro5041sup7.png

RTs_5_00001.cbf. DOI: 10.1107/S2052252524007784/ro5041sup8.png

RTs_7_00001.cbf. DOI: 10.1107/S2052252524007784/ro5041sup9.png

checkCIF/PLATON report. DOI: 10.1107/S2052252524007784/ro5041sup10.pdf

Data collection setup. DOI: 10.1107/S2052252524007784/ro5041sup11.pdf

Supporting tables and figures. DOI: 10.1107/S2052252524007784/ro5041sup12.pdf

PDB reference: ultrahigh-resolution crambin RT structure, 9ewk

## Figures and Tables

**Table 1:** Data processing and refinement statistics

Data processing	
Temperature (K)	297
Wavelength (Å)	0.39995
Resolution range (Å)†	14.47–0.70 (0.71–0.70)
Space group	*P*2_1_
Unit cell (Å, °)	*a* = 22.73, *b* = 18.77, *c* = 41.06, β = 90.55
Unique reflections, Bijvoet pairs merged	54039 (1320)
Multiplicity	13.7 (14.3)
Completeness (%)	98.2 (99.0)
Mean *I*/σ(*I*)	68.4 (2.0)
Wilson *B* factor (Å^2^)	1.14
*R*_merge_ ‡	0.048 (2.218)
*R*_p.i.m._	0.013 (0.569)
CC_1/2_§	1.00 (0.62)
Refinement	
Resolution range (Å)	14.47–0.70
Reflections	47117
*R* factor (*F*_o_> 4σ/all)¶	0.0591/0.0759
No. of atoms (all/hydrogen)	1027/481
Protein (all/hydrogen)	915/451
Non-protein (all/hydrogen)	112/30
RMSD (bonds) (Å)	0.016
RMSD (angles) (°)	1.77
Average *B* factor (Å^2^)	6.58
Protein (all/main/side chain)	5.37/2.49/6.45
Solvent	16.49
Ramachandran plot (preferred/allowed/outliers) (%)††	100/0/0

† Values in parentheses correspond to the highest-resolution shell.

‡ *R*_merge_ = Σ*h*Σ*j*|*Ihj* − 〈*Ih*〉|/Σ*h*Σ*jIhj*, where *Ihj* is the intensity of observation *j* of reflection *h*.

§ As defined by Karplus & Diederichs (2012 ▸).

¶ *R* = Σ*h*|*F*_o_| − |*F*_c_|/Σ*h*|*F*_o_| for all reflections, where *F*_o_ and *F*_c_ are the observed and calculated structure factors, respectively. *R*_free_ is calculated analogously for the test reflections, randomly selected and excluded from the refinement.

†† As defined by *MolProbity* (Davis *et al.*, 2004 ▸).

## Data Availability

The authors confirm that the data supporting the findings of this study are available within the article and its supplementary materials.

## References

[bb1] Adams, P. D., Afonine, P. V., Bunkóczi, G., Chen, V. B., Davis, I. W., Echols, N., Headd, J. J., Hung, L.-W., Kapral, G. J., Grosse-Kunstleve, R. W., McCoy, A. J., Moriarty, N. W., Oeffner, R., Read, R. J., Richardson, D. C., Richardson, J. S., Terwilliger, T. C. & Zwart, P. H. (2010). *Acta Cryst.* D**66**, 213–221. 10.1107/S0907444909052925 PMC2815670 20124702

[bb2] Ahn, H. C., Juranić, N., Macura, S. & Markley, J. L. (2006). *J. Am. Chem. Soc.* **128**, 4398–4404. 10.1021/ja057773d PMC2533276 16569017

[bb3] Bonvin, A. M., Rullmann, J. A., Lamerichs, R. M., Boelens, R. & Kaptein, R. (1993). *Proteins*, **15**, 385–400. 10.1002/prot.340150406 8460109

[bb4] Borek, D., Cymborowski, M., Machius, M., Minor, W. & Otwinowski, Z. (2010). *Acta Cryst.* D**66**, 426–436. 10.1107/S0907444909040177 PMC2852307 20382996

[bb5] Borek, D., Dauter, Z. & Otwinowski, Z. (2013). *J. Synchrotron Rad.* **20**, 37–48. 10.1107/S0909049512048807 PMC3526920 23254654

[bb6] Borek, D., Ginell, S. L., Cymborowski, M., Minor, W. & Otwinowski, Z. (2007). *J. Synchrotron Rad.* **14**, 24–33. 10.1107/S0909049506046589 17211069

[bb7] Borek, D., Minor, W. & Otwinowski, Z. (2003). *Acta Cryst.* D**59**, 2031–2038. 10.1107/s0907444903020924 14573959

[bb900] Burley, S. K., Berman, H. M., Christie, C., Duarte, J. M., Feng, Z., Westbrook, J., Young, J. & Zardecki, C. (2018). *Protein Sci.* **27**, 316–330. 10.1002/pro.3331 PMC5734314 29067736

[bb8] Bury, C. S., Brooks–Bartlett, J. C., Walsh, S. P. & Garman, E. F. (2018). *Protein Sci.* **27**, 217–228. 10.1002/pro.3302 PMC5734275 28921782

[bb10] Chen, J. C., Hanson, B. L., Fisher, S. Z., Langan, P. & Kovalevsky, A. Y. (2012). *Proc. Natl Acad. Sci. USA*, **109**, 15301–15306. 10.1073/pnas.1208341109 PMC3458323 22949690

[bb9] Chen, J. C.-H., Fisher, Z., Kovalevsky, A. Y., Mustyakimov, M., Hanson, B. L., Zhurov, V. V. & Langan, P. (2012). *Acta Cryst.* F**68**, 119–123. 10.1107/S1744309111051499 PMC3274385 22297981

[bb11] Davis, I. W., Murray, L. W., Richardson, J. S. & Richardson, D. C. (2004). *Nucleic Acids Res.* **32**, W615–W619. 10.1093/nar/gkh398 PMC441536 15215462

[bb12] Diederichs, K., McSweeney, S. & Ravelli, R. B. G. (2003). *Acta Cryst.* D**59**, 903–909. 10.1107/s0907444903006516 12777808

[bb13] Emsley, P. & Cowtan, K. (2004). *Acta Cryst.* D**60**, 2126–2132. 10.1107/S0907444904019158 15572765

[bb14] Engh, R. A. & Huber, R. (1991). *Acta Cryst.* A**47**, 392–400.

[bb15] Engh, R. A. & Huber, R. (2001). *International Tables for Crystallography*, Vol. F, edited by M. G. Rossmann & E. Arnold, pp. 382–392. Dordrecht: Kluwer Academic Publishers.

[bb101] Groom, C. R., Bruno, I. J., Lightfoot, M. P. & Ward, S. C. (2016). *Acta Cryst.* B**72**, 171–179. 10.1107/S2052520616003954 PMC4822653 27048719

[bb16] Hendrickson, W. A. & Teeter, M. M. (1981). *Nature*, **290**, 107–113. 10.1038/290107a0 PMC5536114 28769131

[bb17] Holton, J. M., Classen, S., Frankel, K. A. & Tainer, J. A. (2014). *FEBS J.* **281**, 4046–4060. 10.1111/febs.12922 PMC4282448 25040949

[bb18] Howard, E. I., Sanishvili, R., Cachau, R. E., Mitschler, A., Chevrier, B., Barth, P., Lamour, V., Van Zandt, M., Sibley, E., Bon, C., Moras, D., Schneider, T. R., Joachimiak, A. & Podjarny, A. (2004). *Proteins*, **55**, 792–804. 10.1002/prot.20015 15146478

[bb19] Jelsch, C., Teeter, M. M., Lamzin, V., Pichon-Pesme, V., Blessing, R. H. & Lecomte, C. (2000). *Proc. Natl Acad. Sci. USA*, **97**, 3171–3176. 10.1073/pnas.97.7.3171 PMC16211 10737790

[bb20] Karplus, P. A. & Diederichs, K. (2012). *Science*, **336**, 1030–1033. 10.1126/science.1218231 PMC3457925 22628654

[bb103] Lamzin, V. S., Morris, R. J., Dauter, Z., Wilson, K. S. & Teeter, M. M. (1999). *J. Biol. Chem.* **274**, 20753–20755. 10.1074/jbc.274.30.20753 10409612

[bb21] Lobb, L., Stec, B., Kantrowitz, E. K., Yamano, A., Stojanoff, V., Markman, O. & Teeter, M. M. (1996). *Protein Eng. Des. Sel.* **9**, 1233–1239. 10.1093/protein/9.12.1233 9010938

[bb22] Mondal, S. & Bagchi, B. (2022). *Curr. Opin. Struct. Biol.* **77**, 102462. 10.1016/j.sbi.2022.102462 36150344

[bb23] Mondal, S., Mukherjee, S. & Bagchi, B. (2017). *J. Phys. Chem. Lett.* **8**, 4878–4882. 10.1021/acs.jpclett.7b02324 28978201

[bb24] Mora, E. de la, Coquelle, N., Bury, C. S., Rosenthal, M., Holton, J. M., Carmichael, I., Garman, E. F., Burghammer, M., Colletier, J. P. & Weik, M. (2020). *Proc. Natl Acad. Sci. USA*, **117**, 4142–4151. 10.1073/pnas.1821522117 PMC7049125 32047034

[bb102] Murshudov, G. N., Vagin, A. A. & Dodson, E. J. (1997). *Acta Cryst.* D**53**, 240–255. 10.1107/S0907444996012255 15299926

[bb25] Otwinowski, Z., Borek, D., Majewski, W. & Minor, W. (2003). *Acta Cryst.* A**59**, 228–234. 10.1107/s0108767303005488 12714773

[bb26] Otwinowski, Z. & Minor, W. (1997). *Methods Enzymol.* **276**, 307–326. 10.1016/S0076-6879(97)76066-X 27754618

[bb27] Pal, A., Debreczeni, J. É., Sevvana, M., Gruene, T., Kahle, B., Zeeck, A. & Sheldrick, G. M. (2008). *Acta Cryst.* D**64**, 985–992. 10.1107/S0907444908022646 18703848

[bb28] Pauling, L. (1969). *Proc. Natl Acad. Sci. USA*, **64**, 807–809. 10.1073/pnas.64.3.807 PMC223305 16591799

[bb29] Petrova, T., Ginell, S., Mitschler, A., Kim, Y., Lunin, V. Y., Joachimiak, G., Cousido-Siah, A., Hazemann, I., Podjarny, A., Lazarski, K. & Joachimiak, A. (2010). *Acta Cryst.* D**66**, 1075–1091. 10.1107/S0907444910033986 20944241

[bb30] Rosenbaum, G., Alkire, R. W., Evans, G., Rotella, F. J., Lazarski, K., Zhang, R.-G., Ginell, S. L., Duke, N., Naday, I., Lazarz, J., Molitsky, M. J., Keefe, L., Gonczy, J., Rock, L., Sanishvili, R., Walsh, M. A., Westbrook, E. & Joachimiak, A. (2006). *J. Synchrotron Rad.* **13**, 30–45. 10.1107/S0909049505036721 PMC2603069 16371706

[bb31] Rosenbaum, G., Ginell, S. L. & Chen, J. C.-H. (2015). *J. Synchrotron Rad.* **22**, 172–174. 10.1107/S1600577514022619 25537605

[bb32] Schmidt, A., Teeter, M., Weckert, E. & Lamzin, V. S. (2011). *Acta Cryst.* F**67**, 424–428. 10.1107/S1744309110052607 PMC3080141 21505232

[bb33] Schrader-Fischer, G. & Apel, K. (1994). *Mol. Gen. Genet.* **245**, 380–389. 10.1007/BF00290119 7816048

[bb34] Sheldrick, G. M. (2015). *Acta Cryst.* C**71**, 3–8.

[bb36] Spek, A. L. (2020). *Acta Cryst.* E**76**, 1–11. 10.1107/S2056989019016244 PMC6944088 31921444

[bb37] Stec, B., Rao, U. & Teeter, M. M. (1995). *Acta Cryst.* D**51**, 914–924. 10.1107/S0907444995002976 15299761

[bb38] Teeter, M. M. (1984). *Proc. Natl Acad. Sci. USA*, **81**, 6014–6018. 10.1073/pnas.81.19.6014 PMC391849 16593516

[bb39] Teeter, M. M. & Hendrickson, W. A. (1979). *J. Mol. Biol.* **127**, 219–223. 10.1016/0022-2836(79)90242-0 430565

[bb40] Teeter, M. M., Roe, S. M. & Heo, N. H. (1993). *J. Mol. Biol.* **230**, 292–311. 10.1006/jmbi.1993.1143 8450543

[bb41] Teeter, M. M., Yamano, A., Stec, B. & Mohanty, U. (2001). *Proc. Natl Acad. Sci. USA*, **98**, 11242–11247. 10.1073/pnas.201404398 PMC58714 11572978

[bb42] Tikhonov, A. N. & Arsenin, V. I. A. (1977). *Solutions of Ill-posed Problems*. Winston.

[bb43] VanEtten, C. H., Nielsen, H. C. & Peters, J. E. (1965). *Phytochemistry*, **4**, 467–473.

[bb44] Wang, J., Dauter, M., Alkire, R., Joachimiak, A. & Dauter, Z. (2007). *Acta Cryst.* D**63**, 1254–1268. 10.1107/S0907444907054224 18084073

[bb45] Yamano, A., Heo, N. H. & Teeter, M. M. (1997). *J. Biol. Chem.* **272**, 9597–9600. 10.1074/jbc.272.15.9597 9092482

