# Supplementary figures and images for: Solvent organization in the ultrahigh-resolution crystal structure of crambin at room temperature
Source: IUCrJ. 2024 Aug 27;11(Pt 5):649–63. doi: 10.1107/S2052252524007784 (PMC11364037; doi:10.1107/S2052252524007784)

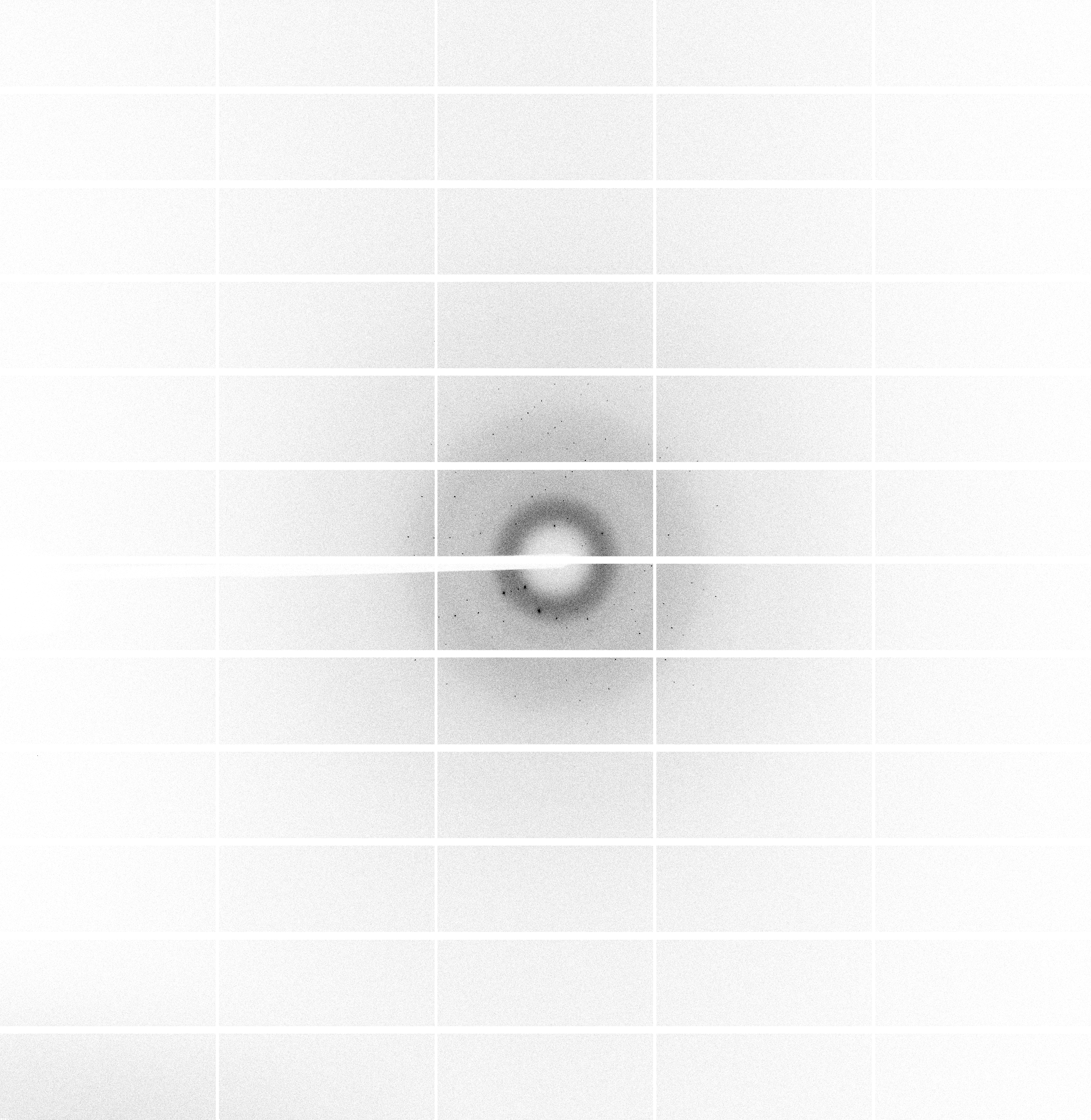

Supplement: Supplementary file 5 [file m-11-00649-sup5.png]

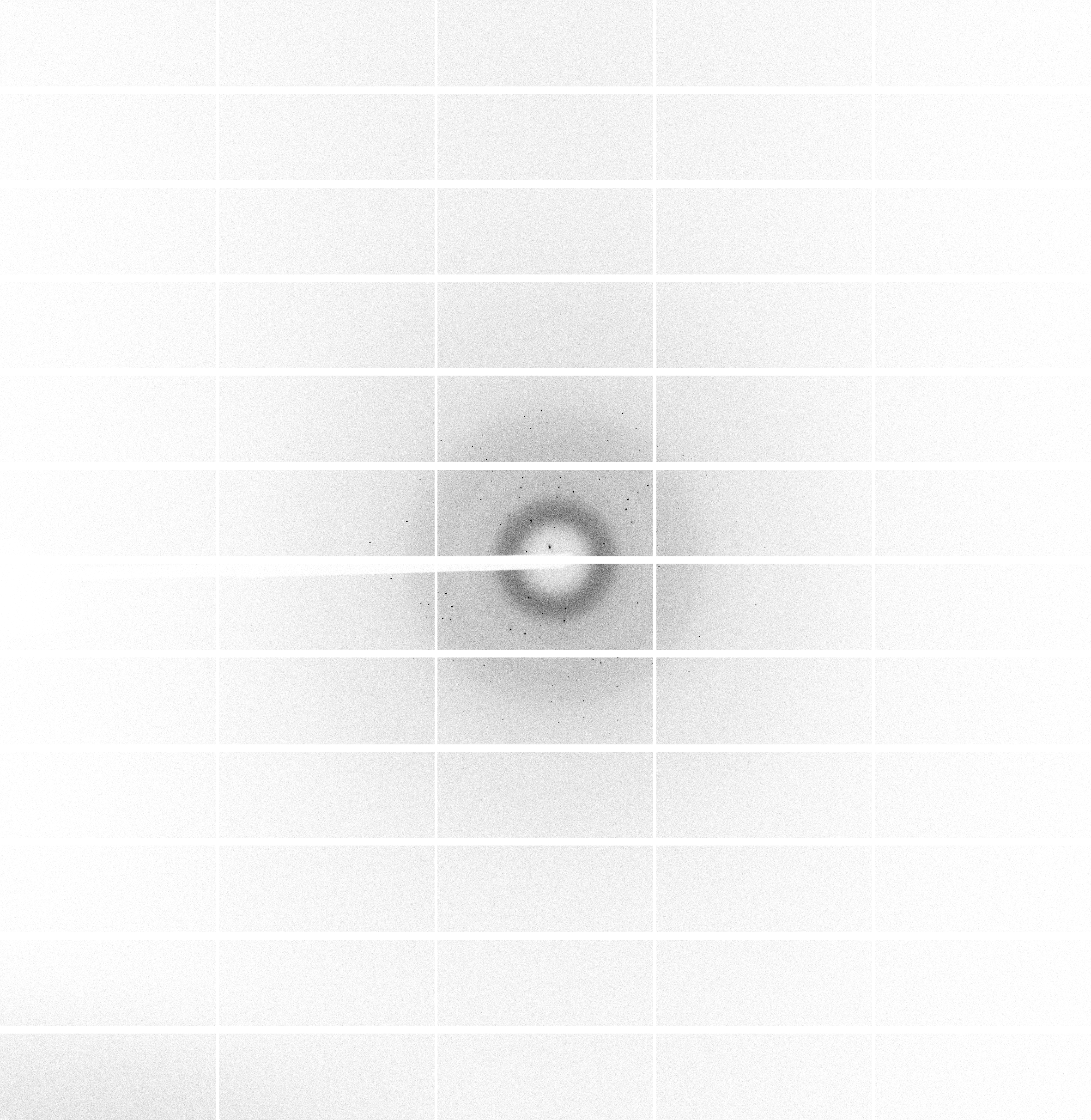

Supplement: Supplementary file 6 [file m-11-00649-sup6.png]

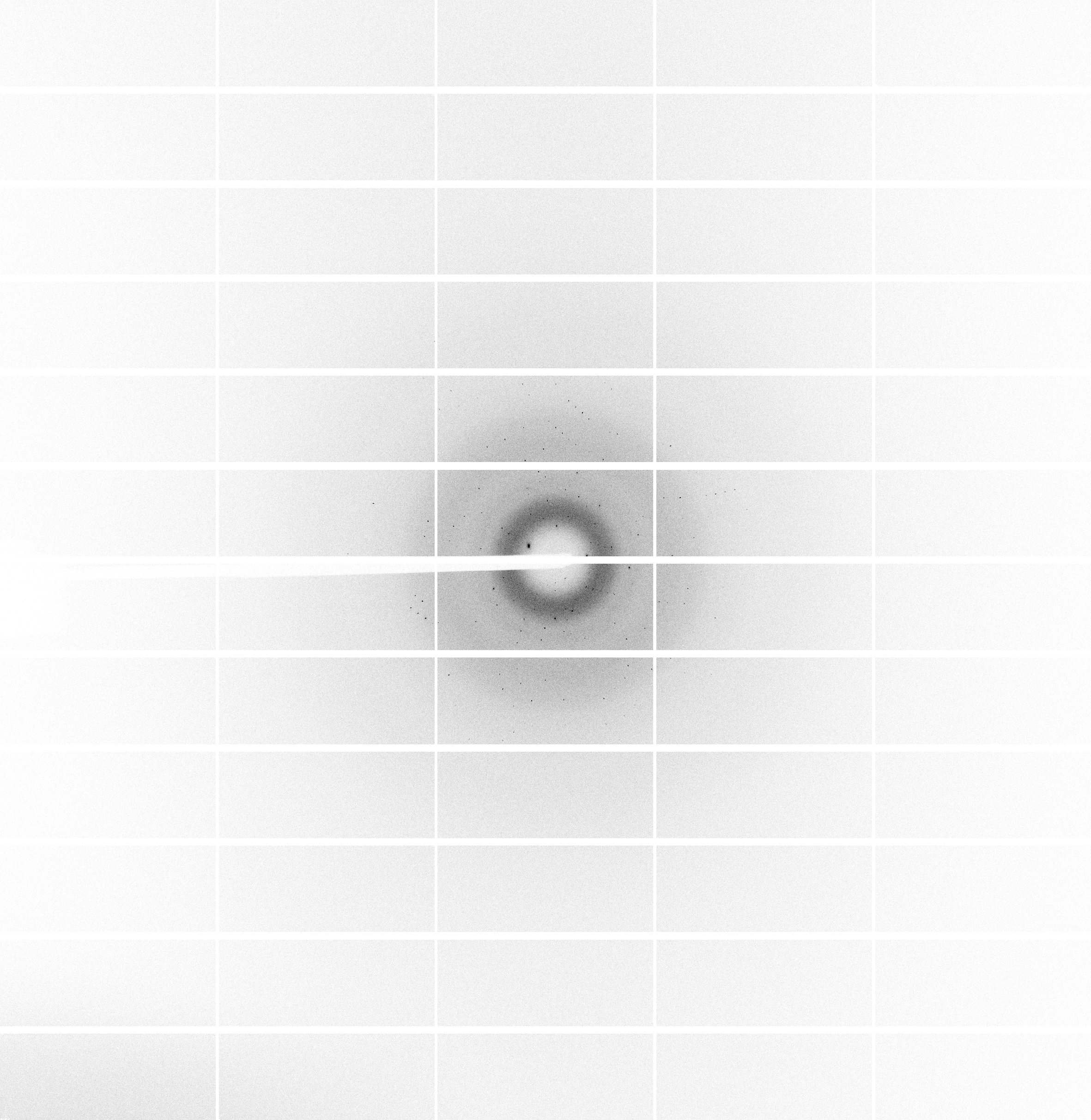

Supplement: Supplementary file 7 [file m-11-00649-sup7.png]

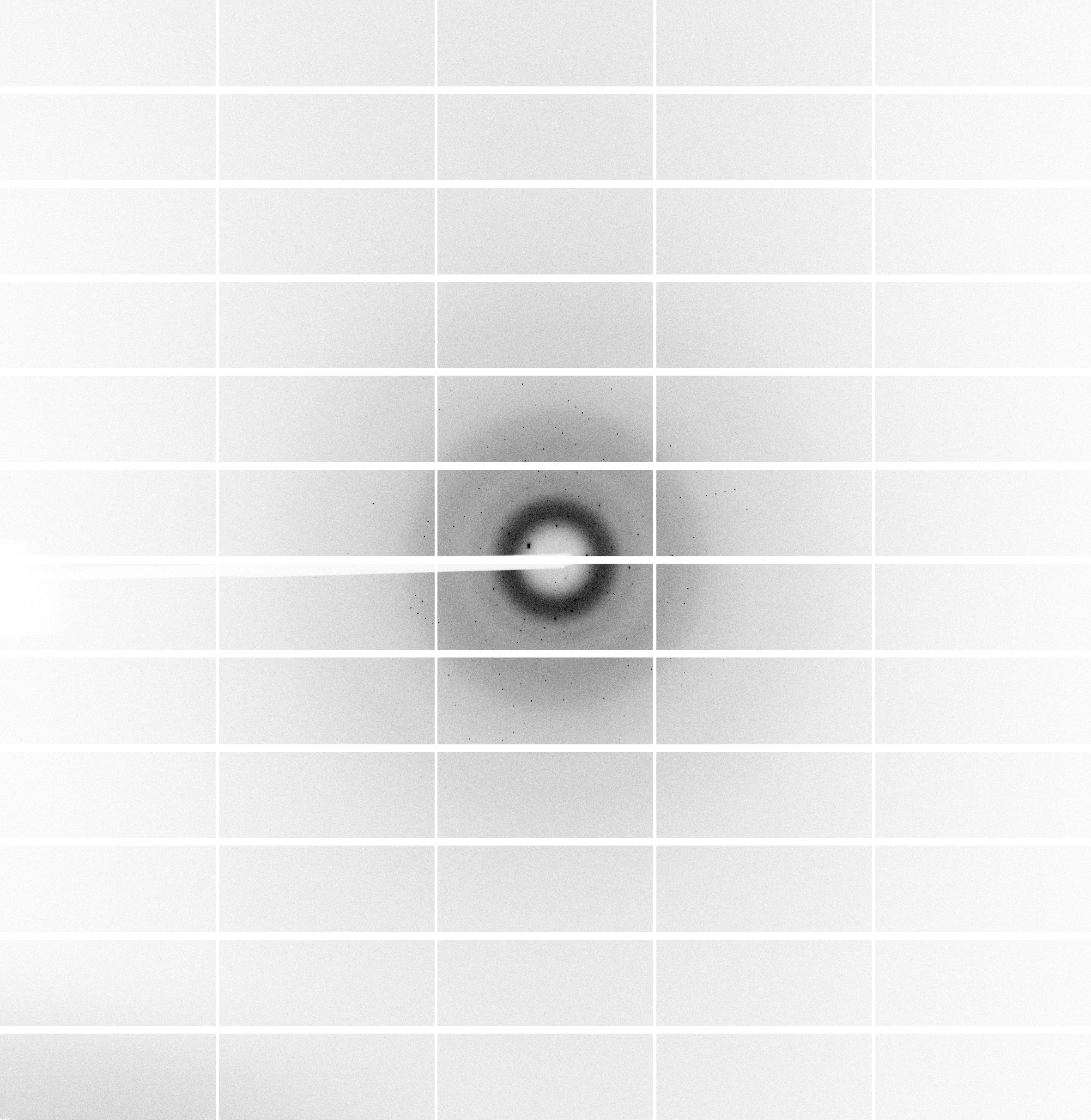

Supplement: Supplementary file 8 [file m-11-00649-sup8.png]

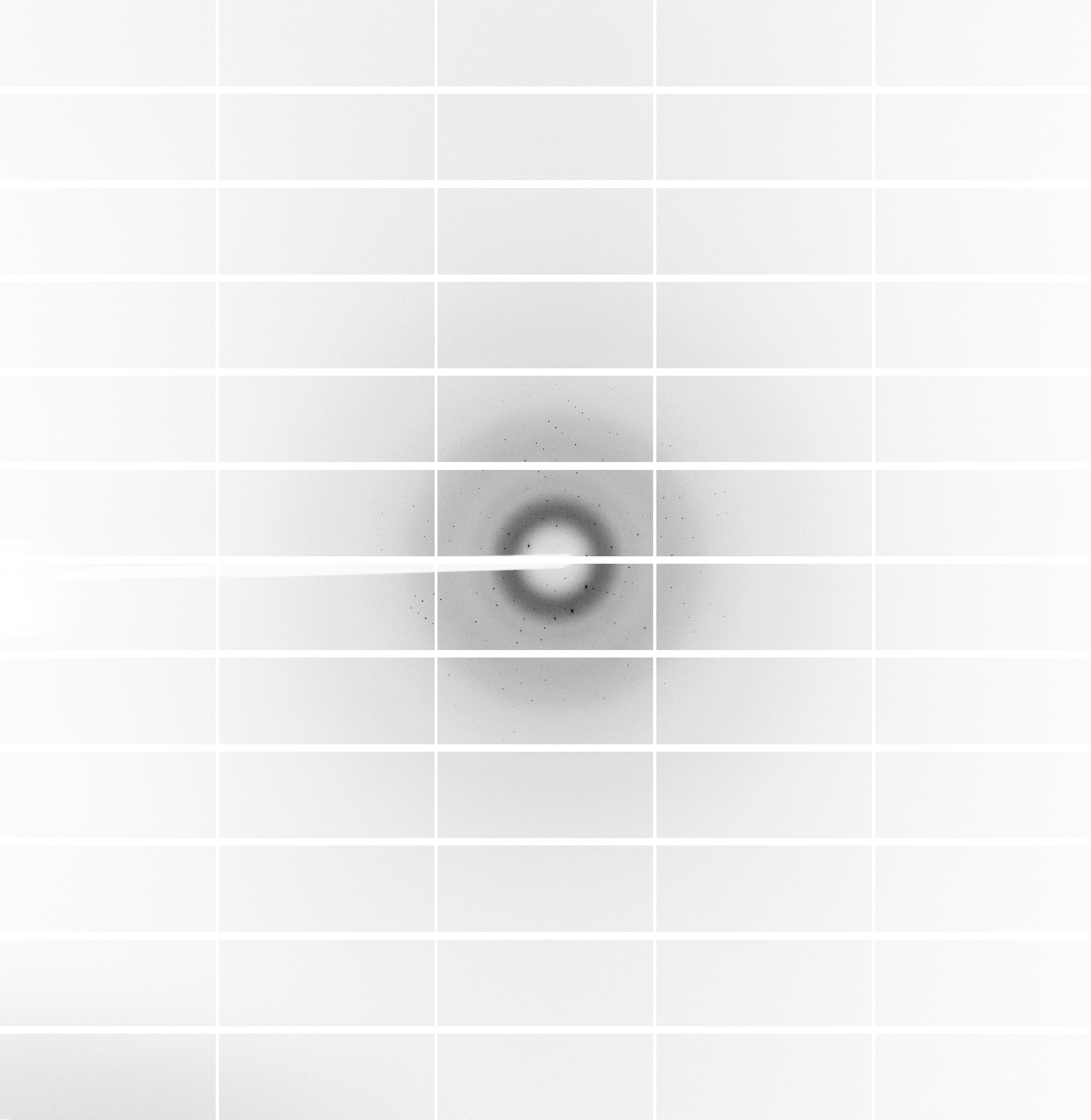

Supplement: Supplementary file 9 [file m-11-00649-sup9.png]

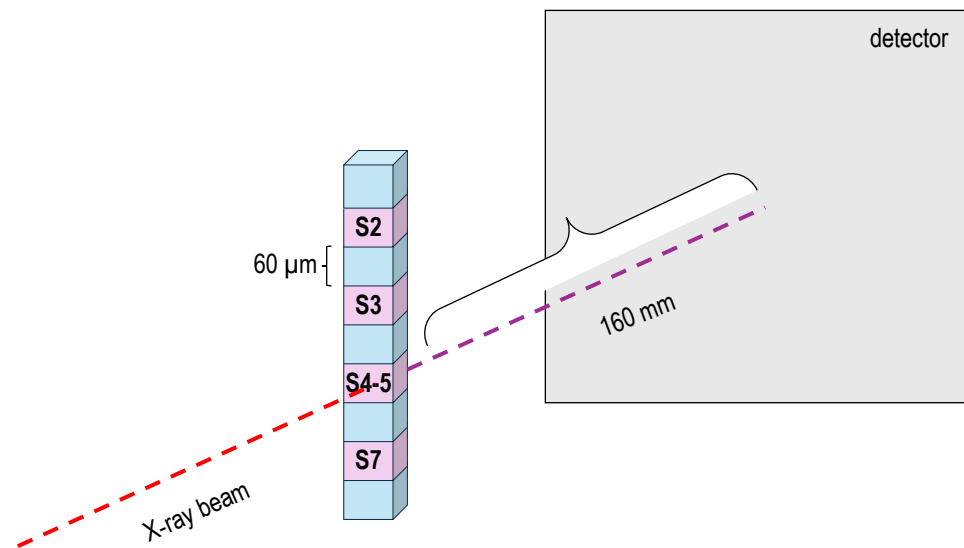

Supplement: Supplementary file 11 [file m-11-00649-sup11.pdf]
