# Supplementary material for: Solvent organization in the ultrahigh-resolution crystal structure of crambin at room temperature
Source: IUCrJ. 2024 Aug 27;11(Pt 5):649–63. doi: 10.1107/S2052252524007784 (PMC11364037; doi:10.1107/S2052252524007784)

checkCIF/PLATON report

Structure factors have been supplied for datablock(s) crambin_rt

THIS REPORT IS FOR GUIDANCE ONLY. IF USED AS PART OF A REVIEW PROCEDURE FOR PUBLICATION, IT SHOULD NOT REPLACE THE EXPERTISE OF AN EXPERIENCED CRYSTALLOGRAPHIC REFEREE.

No syntax errors found. CIF dictionary Interpreting this report

Datablock: crambin_rt

Bond precision: C-C = 0.0015 A Wavelength=0.39950

Cell:	a=22.74(2)	b=18.777(19)	c=41.07(4)
	alpha=90	beta=90.54(5)	gamma=90
Temperature:	293 K		

	Calculated	Reported
Volume	17536(29)	17533(31)
Space group	P 21	P 21
Hall group	P 2yb	P 2yb
Moiety formula	C200.33 H311.74 N54.92 O64.56 S6, C1.26 O5.04, C2 O, O1.34, 29.	?
Sum formula	C203.59 H336.57 N54.92 O114.67 S6 [+ solvent]	C203.59 H336.57 N54.92 O114.67 S6
Mr	5580.89	5580.87
Dx,g cm-3	1.057	1.057
Z	2	2
Mu (mm-1)	0.038	0.038
F000	5911.9	5912.0
F000'	5911.52	
h,k,lmax	32,26,58	0,0,0
Nref	106871[54930]	47115
Tmin,Tmax	0.992,0.992	
Tmin'	0.992	

Correction method= Not given

Data completeness= 0.86/0.44 Theta(max)= 16.569

R(reflections)= 0.0613(40587) wR2(reflections)= 0.1698(47115)

S = 1.141 Npar= 114

The following ALERTS were generated. Each ALERT has the format
test-name_ALERT_alert-type_alert-level.
Click on the hyperlinks for more details of the test.

Alert level A

PLAT213_ALERT_2_A Atom O_A:7 has ADP max/min Ratio ..... 7.1 prolat
PLAT213_ALERT_2_A Atom O_A:10 has ADP max/min Ratio ..... 6.8 prolat
PLAT213_ALERT_2_A Atom O_A:11 has ADP max/min Ratio ..... 5.9 oblate
PLAT213_ALERT_2_A Atom O_A:12 has ADP max/min Ratio ..... 9.2 prolat
PLAT213_ALERT_2_A Atom Og1_A:39 has ADP max/min Ratio ..... 12.9 prolat
PLAT213_ALERT_2_A Atom O_A:45 has ADP max/min Ratio ..... 7.0 prolat
PLAT213_ALERT_2_A Atom N_A:10 has ADP max/min Ratio ..... 7.5 prolat
PLAT213_ALERT_2_A Atom N_A:11 has ADP max/min Ratio ..... 8.3 prolat
PLAT213_ALERT_2_A Atom Nx_A:13 has ADP max/min Ratio ..... 5.2 oblate
PLAT213_ALERT_2_A Atom N_A:14 has ADP max/min Ratio ..... 6.6 oblate
PLAT213_ALERT_2_A Atom N_A:17 has ADP max/min Ratio ..... 5.6 prolat
PLAT213_ALERT_2_A Atom N_A:27 has ADP max/min Ratio ..... 8.5 prolat
PLAT213_ALERT_2_A Atom N_A:46 has ADP max/min Ratio ..... 10.7 prolat
PLAT213_ALERT_2_A Atom Ox_A:7 has ADP max/min Ratio ..... 8.2 prolat
PLAT213_ALERT_2_A Atom Ox_A:8 has ADP max/min Ratio ..... 6.4 prolat
PLAT213_ALERT_2_A Atom O_A:13 has ADP max/min Ratio ..... 5.9 prolat
PLAT213_ALERT_2_A Atom Ox_A:19 has ADP max/min Ratio ..... 5.5 prolat
PLAT213_ALERT_2_A Atom OgY_A:22 has ADP max/min Ratio ..... 5.2 prolat
PLAT213_ALERT_2_A Atom Ox_A:25 has ADP max/min Ratio ..... 8.3 prolat
PLAT213_ALERT_2_A Atom Oh_A:29 has ADP max/min Ratio ..... 6.2 prolat
PLAT213_ALERT_2_A Atom O_A:29 has ADP max/min Ratio ..... 7.6 prolat
PLAT213_ALERT_2_A Atom Ox_A:29 has ADP max/min Ratio ..... 7.5 oblate
PLAT213_ALERT_2_A Atom Oy_A:29 has ADP max/min Ratio ..... 7.6 prolat
PLAT213_ALERT_2_A Atom Ox_A:43 has ADP max/min Ratio ..... 9.4 prolat
PLAT213_ALERT_2_A Atom C_A:7 has ADP max/min Ratio ..... 10.0 prolat
PLAT213_ALERT_2_A Atom Ca_A:7 has ADP max/min Ratio ..... 5.2 prolat
PLAT213_ALERT_2_A Atom Cg2_A:7 has ADP max/min Ratio ..... 25.7 prolat
PLAT213_ALERT_2_A Atom Cb_A:7 has ADP max/min Ratio ..... 8.9 prolat
PLAT213_ALERT_2_A Atom Ca_A:10 has ADP max/min Ratio ..... 9.7 prolat
PLAT213_ALERT_2_A Atom Cb_A:10 has ADP max/min Ratio ..... 9.8 oblate
PLAT213_ALERT_2_A Atom Ca_A:11 has ADP max/min Ratio ..... 6.5 prolat
PLAT213_ALERT_2_A Atom C_A:12 has ADP max/min Ratio ..... 5.6 oblate
PLAT213_ALERT_2_A Atom CaX_A:13 has ADP max/min Ratio ..... 6.8 prolat
PLAT213_ALERT_2_A Atom CbX_A:13 has ADP max/min Ratio ..... 9.3 prolat
PLAT213_ALERT_2_A Atom CgX_A:13 has ADP max/min Ratio ..... 6.5 prolat
PLAT213_ALERT_2_A Atom CzX_A:13 has ADP max/min Ratio ..... 8.9 prolat
PLAT213_ALERT_2_A Atom Cx_A:13 has ADP max/min Ratio ..... 5.6 prolat
PLAT213_ALERT_2_A Atom Ca_A:15 has ADP max/min Ratio ..... 5.1 prolat
PLAT213_ALERT_2_A Atom Cb_A:15 has ADP max/min Ratio ..... 7.2 prolat
PLAT213_ALERT_2_A Atom Cg2_A:15 has ADP max/min Ratio ..... 5.3 prolat
PLAT213_ALERT_2_A Atom CaX_A:37 has ADP max/min Ratio ..... 11.9 prolat
PLAT213_ALERT_2_A Atom Cx_A:37 has ADP max/min Ratio ..... 7.3 oblate
PLAT213_ALERT_2_A Atom Cb_A:39 has ADP max/min Ratio ..... 9.2 prolat
PLAT213_ALERT_2_A Atom C_A:39 has ADP max/min Ratio ..... 9.2 prolat
PLAT213_ALERT_2_A Atom Cg_A:43 has ADP max/min Ratio ..... 14.0 prolat

```
PLAT213_ALERT_2_A Atom Ca_A:46         has ADP max/min Ratio .....         5.9 prolat
PLAT213_ALERT_2_A Atom Nx_A:7          has ADP max/min Ratio .....         6.7 prolat
PLAT213_ALERT_2_A Atom N_A:8           has ADP max/min Ratio .....        11.2 prolat
PLAT213_ALERT_2_A Atom Nx_A:8          has ADP max/min Ratio .....         7.4 prolat
PLAT213_ALERT_2_A Atom Ny_A:8          has ADP max/min Ratio .....         7.5 prolat
PLAT213_ALERT_2_A Atom Nx_A:19         has ADP max/min Ratio .....         6.0 prolat
PLAT213_ALERT_2_A Atom N_A:22          has ADP max/min Ratio .....         6.4 prolat
PLAT213_ALERT_2_A Atom Nx_A:22         has ADP max/min Ratio .....         5.4 prolat
PLAT213_ALERT_2_A Atom Ny_A:22         has ADP max/min Ratio .....         8.3 prolat
PLAT213_ALERT_2_A Atom N_A:25          has ADP max/min Ratio .....        10.5 prolat
PLAT213_ALERT_2_A Atom Nx_A:25         has ADP max/min Ratio .....         5.1 prolat
PLAT213_ALERT_2_A Atom Ny_A:25         has ADP max/min Ratio .....         8.9 prolat
PLAT213_ALERT_2_A Atom Ny_A:29         has ADP max/min Ratio .....         7.1 prolat
PLAT213_ALERT_2_A Atom CaX_A:7         has ADP max/min Ratio .....        11.3 prolat
PLAT213_ALERT_2_A Atom Cg2X_A:7        has ADP max/min Ratio .....         8.4 prolat
PLAT213_ALERT_2_A Atom Cb_A:8          has ADP max/min Ratio .....         5.5 prolat
PLAT213_ALERT_2_A Atom Cg2_A:8         has ADP max/min Ratio .....         5.7 oblate
PLAT213_ALERT_2_A Atom CaX_A:8         has ADP max/min Ratio .....         6.1 prolat
PLAT213_ALERT_2_A Atom Cx_A:8          has ADP max/min Ratio .....         9.1 prolat
PLAT213_ALERT_2_A Atom CaY_A:8         has ADP max/min Ratio .....        11.7 prolat
PLAT213_ALERT_2_A Atom Cg2Y_A:8        has ADP max/min Ratio .....        13.0 prolat
PLAT213_ALERT_2_A Atom Cy_A:8          has ADP max/min Ratio .....        17.0 prolat
PLAT213_ALERT_2_A Atom CbX_A:10        has ADP max/min Ratio .....         6.7 prolat
PLAT213_ALERT_2_A Atom CdX_A:10        has ADP max/min Ratio .....         6.1 prolat
PLAT213_ALERT_2_A Atom CgX_A:10        has ADP max/min Ratio .....         6.8 prolat
PLAT213_ALERT_2_A Atom Ca_A:13         has ADP max/min Ratio .....         9.7 prolat
PLAT213_ALERT_2_A Atom Cb_A:13         has ADP max/min Ratio .....         6.5 prolat
PLAT213_ALERT_2_A Atom CaX_A:19        has ADP max/min Ratio .....         9.6 prolat
PLAT213_ALERT_2_A Atom CbX_A:19        has ADP max/min Ratio .....        13.6 prolat
PLAT213_ALERT_2_A Atom Ca_A:22         has ADP max/min Ratio .....         5.5 prolat
PLAT213_ALERT_2_A Atom C_A:22          has ADP max/min Ratio .....         5.6 prolat
PLAT213_ALERT_2_A Atom CaX_A:22        has ADP max/min Ratio .....         7.1 prolat
PLAT213_ALERT_2_A Atom Cx_A:22         has ADP max/min Ratio .....         5.2 prolat
PLAT213_ALERT_2_A Atom CaY_A:22        has ADP max/min Ratio .....         7.0 prolat
PLAT213_ALERT_2_A Atom CbY_A:22        has ADP max/min Ratio .....         7.4 prolat
PLAT213_ALERT_2_A Atom Cy_A:22         has ADP max/min Ratio .....         6.5 prolat
PLAT213_ALERT_2_A Atom Cb_A:25         has ADP max/min Ratio .....         8.7 prolat
PLAT213_ALERT_2_A Atom Cg1_A:25        has ADP max/min Ratio .....         9.5 prolat
PLAT213_ALERT_2_A Atom CaX_A:25        has ADP max/min Ratio .....         6.2 prolat
PLAT213_ALERT_2_A Atom CbX_A:25        has ADP max/min Ratio .....         6.8 oblate
PLAT213_ALERT_2_A Atom Cd1X_A:25       has ADP max/min Ratio .....        13.5 prolat
PLAT213_ALERT_2_A Atom CgX_A:25        has ADP max/min Ratio .....        10.1 prolat
PLAT213_ALERT_2_A Atom Cx_A:25         has ADP max/min Ratio .....         5.5 prolat
PLAT213_ALERT_2_A Atom CbY_A:25        has ADP max/min Ratio .....         7.5 oblate
PLAT213_ALERT_2_A Atom Cd1Y_A:25       has ADP max/min Ratio .....        13.3 prolat
PLAT213_ALERT_2_A Atom Cy_A:25         has ADP max/min Ratio .....         5.3 prolat
PLAT213_ALERT_2_A Atom Ca_A:29         has ADP max/min Ratio .....         9.4 prolat
PLAT213_ALERT_2_A Atom Cb_A:29         has ADP max/min Ratio .....         5.7 prolat
PLAT213_ALERT_2_A Atom Ce1_A:29        has ADP max/min Ratio .....        11.8 oblate
PLAT213_ALERT_2_A Atom CaX_A:29        has ADP max/min Ratio .....         6.0 prolat
PLAT213_ALERT_2_A Atom CgX_A:29        has ADP max/min Ratio .....         9.4 oblate
PLAT213_ALERT_2_A Atom Cd1X_A:29       has ADP max/min Ratio .....         9.5 prolat
PLAT213_ALERT_2_A Atom Ce1X_A:29       has ADP max/min Ratio .....         7.7 prolat
PLAT213_ALERT_2_A Atom CzX_A:29        has ADP max/min Ratio .....        17.2 oblate
PLAT213_ALERT_2_A Atom Cx_A:29         has ADP max/min Ratio .....         6.5 prolat
PLAT213_ALERT_2_A Atom CaY_A:29        has ADP max/min Ratio .....        10.7 prolat
PLAT213_ALERT_2_A Atom Cy_A:29         has ADP max/min Ratio .....         8.4 prolat
```

```
PLAT213_ALERT_2_A Atom CaX_A:39        has ADP max/min Ratio .....       13.8 prolat
PLAT213_ALERT_2_A Atom Cg2X_A:39       has ADP max/min Ratio .....       10.7 prolat
PLAT213_ALERT_2_A Atom CaX_A:43        has ADP max/min Ratio .....        6.7 oblate
PLAT213_ALERT_2_A Atom Cx_A:43         has ADP max/min Ratio .....       10.5 prolat
PLAT214_ALERT_2_A Atom C2_A:102   (Anion/Solvent) ADP max/min Ratio       6.4 prolat
PLAT214_ALERT_2_A Atom C1_A:102   (Anion/Solvent) ADP max/min Ratio      34.1 oblate
PLAT214_ALERT_2_A Atom Ox_S:8     (Anion/Solvent) ADP max/min Ratio       7.1 prolat
```

Alert level B

```
PLAT213_ALERT_2_B Atom Sg_A:3          has ADP max/min Ratio .....        4.1 prolat
PLAT213_ALERT_2_B Atom Ox_A:13         has ADP max/min Ratio .....        5.0 prolat
PLAT213_ALERT_2_B Atom O_A:14          has ADP max/min Ratio .....        4.8 prolat
PLAT213_ALERT_2_B Atom O_A:16          has ADP max/min Ratio .....        4.5 oblate
PLAT213_ALERT_2_B Atom O_A:17          has ADP max/min Ratio .....        4.1 prolat
PLAT213_ALERT_2_B Atom O_A:19          has ADP max/min Ratio .....        4.9 prolat
PLAT213_ALERT_2_B Atom Ox_A:37         has ADP max/min Ratio .....        4.4 oblate
PLAT213_ALERT_2_B Atom Od2_A:43        has ADP max/min Ratio .....        4.7 oblate
PLAT213_ALERT_2_B Atom N_A:12          has ADP max/min Ratio .....        4.8 prolat
PLAT213_ALERT_2_B Atom N_A:16          has ADP max/min Ratio .....        4.7 prolat
PLAT213_ALERT_2_B Atom N_A:26          has ADP max/min Ratio .....        4.1 oblate
PLAT213_ALERT_2_B Atom N_A:40          has ADP max/min Ratio .....        4.2 oblate
PLAT213_ALERT_2_B Atom Ox_A:10         has ADP max/min Ratio .....        4.9 oblate
PLAT213_ALERT_2_B Atom Ox_A:22         has ADP max/min Ratio .....        4.3 prolat
PLAT213_ALERT_2_B Atom O_A:25          has ADP max/min Ratio .....        4.5 oblate
PLAT213_ALERT_2_B Atom OhX_A:29        has ADP max/min Ratio .....        4.5 prolat
PLAT213_ALERT_2_B Atom C_A:3           has ADP max/min Ratio .....        5.0 oblate
PLAT213_ALERT_2_B Atom Ca_A:3          has ADP max/min Ratio .....        4.2 oblate
PLAT213_ALERT_2_B Atom Ca_A:4          has ADP max/min Ratio .....        4.8 oblate
PLAT213_ALERT_2_B Atom Cg_A:5          has ADP max/min Ratio .....        4.9 oblate
PLAT213_ALERT_2_B Atom C_A:10          has ADP max/min Ratio .....        4.6 prolat
PLAT213_ALERT_2_B Atom Cb_A:11         has ADP max/min Ratio .....        4.1 prolat
PLAT213_ALERT_2_B Atom C_A:11          has ADP max/min Ratio .....        4.8 prolat
PLAT213_ALERT_2_B Atom Cg1_A:15        has ADP max/min Ratio .....        4.7 prolat
PLAT213_ALERT_2_B Atom C_A:15          has ADP max/min Ratio .....        4.5 prolat
PLAT213_ALERT_2_B Atom C_A:18          has ADP max/min Ratio .....        4.5 prolat
PLAT213_ALERT_2_B Atom Cd_A:19         has ADP max/min Ratio .....        5.0 prolat
PLAT213_ALERT_2_B Atom C_A:45          has ADP max/min Ratio .....        4.6 oblate
PLAT213_ALERT_2_B Atom N_A:13          has ADP max/min Ratio .....        4.9 prolat
PLAT213_ALERT_2_B Atom N_A:29          has ADP max/min Ratio .....        4.4 prolat
PLAT213_ALERT_2_B Atom CaX_A:1         has ADP max/min Ratio .....        4.3 prolat
PLAT213_ALERT_2_B Atom CbX_A:7         has ADP max/min Ratio .....        4.5 prolat
PLAT213_ALERT_2_B Atom CbX_A:8         has ADP max/min Ratio .....        4.3 prolat
PLAT213_ALERT_2_B Atom Cg1Y_A:8        has ADP max/min Ratio .....        4.1 prolat
PLAT213_ALERT_2_B Atom Cx_A:10         has ADP max/min Ratio .....        4.4 prolat
PLAT213_ALERT_2_B Atom C_A:13          has ADP max/min Ratio .....        4.3 prolat
PLAT213_ALERT_2_B Atom Ce1_A:13        has ADP max/min Ratio .....        4.3 prolat
PLAT213_ALERT_2_B Atom CgX_A:19        has ADP max/min Ratio .....        4.8 oblate
PLAT213_ALERT_2_B Atom Ca_A:25         has ADP max/min Ratio .....        4.4 prolat
PLAT213_ALERT_2_B Atom Cd1_A:25        has ADP max/min Ratio .....        4.8 prolat
PLAT213_ALERT_2_B Atom CzY_A:29        has ADP max/min Ratio .....        4.3 oblate
PLAT214_ALERT_2_B Atom O_A:102    (Anion/Solvent) ADP max/min Ratio       5.2 prolat
PLAT214_ALERT_2_B Atom Ox_S:22    (Anion/Solvent) ADP max/min Ratio       5.2 prolat
PLAT220_ALERT_2_B NonSolvent   Resd 1  C   Ueq(max)/Ueq(min) Range       10.0 Ratio
PLAT220_ALERT_2_B NonSolvent   Resd 1  N   Ueq(max)/Ueq(min) Range       10.0 Ratio
PLAT220_ALERT_2_B NonSolvent   Resd 1  O   Ueq(max)/Ueq(min) Range       10.0 Ratio
PLAT260_ALERT_2_B Large Average Ueq of Residue Including   O_A:101      0.524 Check
```

```
PLAT260_ALERT_2_B Large Average Ueq of Residue Including    O_S:81      0.417 Check
PLAT260_ALERT_2_B Large Average Ueq of Residue Including   O_A:102      0.498 Check
PLAT260_ALERT_2_B Large Average Ueq of Residue Including    O_S:31      0.319 Check
PLAT260_ALERT_2_B Large Average Ueq of Residue Including   Ox_S:32      0.322 Check
PLAT260_ALERT_2_B Large Average Ueq of Residue Including    O_S:39      0.435 Check
PLAT260_ALERT_2_B Large Average Ueq of Residue Including   Ox_S:44      0.481 Check
PLAT260_ALERT_2_B Large Average Ueq of Residue Including    O_S:45      0.506 Check
PLAT260_ALERT_2_B Large Average Ueq of Residue Including    O_S:47      0.462 Check
PLAT260_ALERT_2_B Large Average Ueq of Residue Including    O_S:48      0.491 Check
PLAT260_ALERT_2_B Large Average Ueq of Residue Including    O_S:50      0.515 Check
PLAT260_ALERT_2_B Large Average Ueq of Residue Including    O_S:51      0.350 Check
PLAT260_ALERT_2_B Large Average Ueq of Residue Including    O_S:53      0.370 Check
PLAT260_ALERT_2_B Large Average Ueq of Residue Including    O_S:71      0.715 Check
PLAT260_ALERT_2_B Large Average Ueq of Residue Including    O_S:73      0.469 Check
PLAT260_ALERT_2_B Large Average Ueq of Residue Including    Ox_S:8      0.308 Check
PLAT306_ALERT_2_B Isolated Oxygen Atom (H-atoms Missing ?) .......     O_S:81 Check
PLAT306_ALERT_2_B Isolated Oxygen Atom (H-atoms Missing ?) .......     O_S:31 Check
PLAT306_ALERT_2_B Isolated Oxygen Atom (H-atoms Missing ?) .......     O_S:45 Check
PLAT306_ALERT_2_B Isolated Oxygen Atom (H-atoms Missing ?) .......     O_S:48 Check
PLAT306_ALERT_2_B Isolated Oxygen Atom (H-atoms Missing ?) .......     O_S:50 Check
PLAT306_ALERT_2_B Isolated Oxygen Atom (H-atoms Missing ?) .......     O_S:71 Check
PLAT306_ALERT_2_B Isolated Oxygen Atom (H-atoms Missing ?) .......     O_S:73 Check
PLAT420_ALERT_2_B D-H Bond Without Acceptor  O_S:1    --H2_S:1   .     Please Check
PLAT420_ALERT_2_B D-H Bond Without Acceptor  O_S:17   --H2_S:17  .     Please Check
PLAT934_ALERT_3_B Number of (Iobs-Icalc)/Sigma(W) > 10 Outliers ..          7 Check
               -6  1  1,   5  0  2,   1  3  2,   1  4  5,   2  0  6,   6  0 11,
                3  4 11,
```

Alert level C

```
STRVA01_ALERT_4_C           Flack test results are ambiguous.
           From the CIF: _refine_ls_abs_structure_Flack    0.420
           From the CIF: _refine_ls_abs_structure_Flack_su    0.120
PLAT029_ALERT_3_C _diffrn_measured_fraction_theta_full value Low .      0.978 Why?
PLAT052_ALERT_1_C Info on Absorption Correction Method   Not Given     Please Do !
PLAT148_ALERT_3_C s.u. on the      b    - Axis is (Too) Large ....     0.0190 Ang.
PLAT213_ALERT_2_C Atom Og1_A:1        has ADP max/min Ratio .....        3.5 oblate
PLAT213_ALERT_2_C Atom O_A:3          has ADP max/min Ratio .....        3.6 prolat
PLAT213_ALERT_2_C Atom O_A:5          has ADP max/min Ratio .....        3.7 prolat
PLAT213_ALERT_2_C Atom Od1_A:12       has ADP max/min Ratio .....        3.1 oblate
PLAT213_ALERT_2_C Atom O_A:15         has ADP max/min Ratio .....        3.9 prolat
PLAT213_ALERT_2_C Atom O_A:20         has ADP max/min Ratio .....        3.6 prolat
PLAT213_ALERT_2_C Atom O_A:26         has ADP max/min Ratio .....        3.9 prolat
PLAT213_ALERT_2_C Atom O_A:27         has ADP max/min Ratio .....        3.9 prolat
PLAT213_ALERT_2_C Atom O_A:30         has ADP max/min Ratio .....        3.8 oblate
PLAT213_ALERT_2_C Atom Nx_A:2         has ADP max/min Ratio .....        3.9 prolat
PLAT213_ALERT_2_C Atom N_A:4          has ADP max/min Ratio .....        3.9 oblate
PLAT213_ALERT_2_C Atom Nd2_A:12       has ADP max/min Ratio .....        3.7 oblate
PLAT213_ALERT_2_C Atom N_A:15         has ADP max/min Ratio .....        3.6 prolat
PLAT213_ALERT_2_C Atom Nx_A:37        has ADP max/min Ratio .....        3.8 prolat
PLAT213_ALERT_2_C Atom N_A:39         has ADP max/min Ratio .....        4.0 prolat
PLAT213_ALERT_2_C Atom N_A:43         has ADP max/min Ratio .....        3.5 prolat
PLAT213_ALERT_2_C Atom O_A:2          has ADP max/min Ratio .....        3.4 prolat
PLAT213_ALERT_2_C Atom O_A:8          has ADP max/min Ratio .....        3.6 prolat
PLAT213_ALERT_2_C Atom Oy_A:8         has ADP max/min Ratio .....        3.4 prolat
PLAT213_ALERT_2_C Atom Oy_A:25        has ADP max/min Ratio .....        3.9 prolat
PLAT213_ALERT_2_C Atom OhY_A:29       has ADP max/min Ratio .....        3.3 oblate
```

```
PLAT213_ALERT_2_C Atom Og1X_A:39       has ADP max/min Ratio .....         3.9 prolat
PLAT213_ALERT_2_C Atom Cx_A:2          has ADP max/min Ratio .....         3.9 prolat
PLAT213_ALERT_2_C Atom C_A:4           has ADP max/min Ratio .....         3.7 prolat
PLAT213_ALERT_2_C Atom Cb_A:5          has ADP max/min Ratio .....         4.0 prolat
PLAT213_ALERT_2_C Atom Cd_A:5          has ADP max/min Ratio .....         3.1 prolat
PLAT213_ALERT_2_C Atom Cg1_A:7         has ADP max/min Ratio .....         3.8 oblate
PLAT213_ALERT_2_C Atom Cd1_A:7         has ADP max/min Ratio .....         3.6 oblate
PLAT213_ALERT_2_C Atom C_A:9           has ADP max/min Ratio .....         4.0 prolat
PLAT213_ALERT_2_C Atom Ce1X_A:13       has ADP max/min Ratio .....         3.2 prolat
PLAT213_ALERT_2_C Atom C_A:16          has ADP max/min Ratio .....         3.9 prolat
PLAT213_ALERT_2_C Atom Ca_A:17         has ADP max/min Ratio .....         3.8 prolat
PLAT213_ALERT_2_C Atom Cg_A:17         has ADP max/min Ratio .....         3.3 prolat
PLAT213_ALERT_2_C Atom Cd_A:17         has ADP max/min Ratio .....         3.4 oblate
PLAT213_ALERT_2_C Atom Cb_A:18         has ADP max/min Ratio .....         3.9 prolat
PLAT213_ALERT_2_C Atom Cd1_A:18        has ADP max/min Ratio .....         3.5 prolat
PLAT213_ALERT_2_C Atom Cb_A:24         has ADP max/min Ratio .....         3.3 prolat
PLAT213_ALERT_2_C Atom C_A:24          has ADP max/min Ratio .....         3.1 prolat
PLAT213_ALERT_2_C Atom Ca_A:27         has ADP max/min Ratio .....         4.0 prolat
PLAT213_ALERT_2_C Atom C_A:27          has ADP max/min Ratio .....         3.4 prolat
PLAT213_ALERT_2_C Atom Ca_A:30         has ADP max/min Ratio .....         3.6 prolat
PLAT213_ALERT_2_C Atom Cb_A:30         has ADP max/min Ratio .....         3.8 prolat
PLAT213_ALERT_2_C Atom Cd1_A:34        has ADP max/min Ratio .....         3.2 prolat
PLAT213_ALERT_2_C Atom Cb_A:36         has ADP max/min Ratio .....         3.3 prolat
PLAT213_ALERT_2_C Atom Cg_A:36         has ADP max/min Ratio .....         3.1 oblate
PLAT213_ALERT_2_C Atom Ca_A:39         has ADP max/min Ratio .....         3.8 prolat
PLAT213_ALERT_2_C Atom Cb_A:40         has ADP max/min Ratio .....         3.1 prolat
PLAT213_ALERT_2_C Atom Ca_A:40         has ADP max/min Ratio .....         3.9 prolat
PLAT213_ALERT_2_C Atom Cb_A:43         has ADP max/min Ratio .....         3.5 prolat
PLAT213_ALERT_2_C Atom C_A:43          has ADP max/min Ratio .....         3.1 prolat
PLAT213_ALERT_2_C Atom Ca_A:45         has ADP max/min Ratio .....         3.7 prolat
PLAT213_ALERT_2_C Atom N_A:2           has ADP max/min Ratio .....         4.0 prolat
PLAT213_ALERT_2_C Atom Nx_A:10         has ADP max/min Ratio .....         3.8 prolat
PLAT213_ALERT_2_C Atom Nh2X_A:10       has ADP max/min Ratio .....         3.9 prolat
PLAT213_ALERT_2_C Atom Nx_A:39         has ADP max/min Ratio .....         4.0 prolat
PLAT213_ALERT_2_C Atom CbX_A:1         has ADP max/min Ratio .....         3.4 oblate
PLAT213_ALERT_2_C Atom Cg2_A:2         has ADP max/min Ratio .....         3.1 prolat
PLAT213_ALERT_2_C Atom Cb_A:2          has ADP max/min Ratio .....         3.1 prolat
PLAT213_ALERT_2_C Atom Cx_A:7          has ADP max/min Ratio .....         4.0 prolat
PLAT213_ALERT_2_C Atom C_A:8           has ADP max/min Ratio .....         3.4 prolat
PLAT213_ALERT_2_C Atom Cg2X_A:8        has ADP max/min Ratio .....         3.7 prolat
PLAT213_ALERT_2_C Atom Cg1X_A:8        has ADP max/min Ratio .....         3.8 oblate
PLAT213_ALERT_2_C Atom Cg_A:13         has ADP max/min Ratio .....         3.2 oblate
PLAT213_ALERT_2_C Atom CdX_A:19        has ADP max/min Ratio .....         3.7 prolat
PLAT213_ALERT_2_C Atom Cx_A:19         has ADP max/min Ratio .....         3.2 prolat
PLAT213_ALERT_2_C Atom Cb_A:22         has ADP max/min Ratio .....         3.5 prolat
PLAT213_ALERT_2_C Atom C_A:25          has ADP max/min Ratio .....         3.3 prolat
PLAT213_ALERT_2_C Atom CaY_A:25        has ADP max/min Ratio .....         3.6 oblate
PLAT213_ALERT_2_C Atom C_A:29          has ADP max/min Ratio .....         3.1 prolat
PLAT213_ALERT_2_C Atom Ce2Y_A:29       has ADP max/min Ratio .....         3.6 prolat
PLAT213_ALERT_2_C Atom C_A:37          has ADP max/min Ratio .....         3.6 prolat
PLAT213_ALERT_2_C Atom CgX_A:43        has ADP max/min Ratio .....         3.2 prolat
PLAT214_ALERT_2_C Atom O_S:32     (Anion/Solvent) ADP max/min Ratio        5.0 prolat
PLAT214_ALERT_2_C Atom C1X_A:101 (Anion/Solvent) ADP max/min Ratio         4.1 prolat
PLAT218_ALERT_3_C Constrained U(ij) Components(s)  for N1#        .          6 Check
PLAT218_ALERT_3_C Constrained U(ij) Components(s)  for C1#        .          6 Check
PLAT218_ALERT_3_C Constrained U(ij) Components(s)  for C2#        .          6 Check
PLAT218_ALERT_3_C Constrained U(ij) Components(s)  for C3#        .          6 Check
```

```
PLAT218_ALERT_3_C Constrained U(ij) Components(s) for O1#        .          6 Check
PLAT218_ALERT_3_C Constrained U(ij) Components(s) for C4#        .          6 Check
PLAT218_ALERT_3_C Constrained U(ij) Components(s) for O2#        .          6 Check
PLAT218_ALERT_3_C Constrained U(ij) Components(s) for C5#        .          6 Check
PLAT218_ALERT_3_C Constrained U(ij) Components(s) for C6#        .          6 Check
PLAT218_ALERT_3_C Constrained U(ij) Components(s) for N2#        .          6 Check
PLAT218_ALERT_3_C Constrained U(ij) Components(s) for C7#        .          6 Check
PLAT218_ALERT_3_C Constrained U(ij) Components(s) for O3#        .          6 Check
PLAT218_ALERT_3_C Constrained U(ij) Components(s) for C8#        .          6 Check
PLAT218_ALERT_3_C Constrained U(ij) Components(s) for O4#        .          6 Check
PLAT218_ALERT_3_C Constrained U(ij) Components(s) for N3#        .          6 Check
PLAT218_ALERT_3_C Constrained U(ij) Components(s) for O5#        .          6 Check
PLAT218_ALERT_3_C Constrained U(ij) Components(s) for C9#        .          6 Check
PLAT218_ALERT_3_C Constrained U(ij) Components(s) for O6#        .          6 Check
PLAT218_ALERT_3_C Constrained U(ij) Components(s) for C10#       .          6 Check
PLAT218_ALERT_3_C Constrained U(ij) Components(s) for C11#       .          6 Check
PLAT218_ALERT_3_C Constrained U(ij) Components(s) for C12#       .          6 Check
PLAT218_ALERT_3_C Constrained U(ij) Components(s) for C13#       .          6 Check
PLAT218_ALERT_3_C Constrained U(ij) Components(s) for O7#        .          6 Check
PLAT218_ALERT_3_C Constrained U(ij) Components(s) for C14#       .          6 Check
PLAT218_ALERT_3_C Constrained U(ij) Components(s) for O8#        .          6 Check
PLAT218_ALERT_3_C Constrained U(ij) Components(s) for C15#       .          6 Check
PLAT218_ALERT_3_C Constrained U(ij) Components(s) for C16#       .          6 Check
PLAT218_ALERT_3_C Constrained U(ij) Components(s) for N4#        .          6 Check
PLAT218_ALERT_3_C Constrained U(ij) Components(s) for C17#       .          6 Check
PLAT218_ALERT_3_C Constrained U(ij) Components(s) for S1#        .          6 Check
PLAT218_ALERT_3_C Constrained U(ij) Components(s) for O9#        .          6 Check
PLAT218_ALERT_3_C Constrained U(ij) Components(s) for N5#        .          6 Check
PLAT218_ALERT_3_C Constrained U(ij) Components(s) for C18#       .          6 Check
PLAT218_ALERT_3_C Constrained U(ij) Components(s) for C19#       .          6 Check
PLAT218_ALERT_3_C Constrained U(ij) Components(s) for N6#        .          6 Check
PLAT218_ALERT_3_C Constrained U(ij) Components(s) for C20#       .          6 Check
PLAT218_ALERT_3_C Constrained U(ij) Components(s) for O10#       .          6 Check
PLAT218_ALERT_3_C Constrained U(ij) Components(s) for S2#        .          6 Check
PLAT218_ALERT_3_C Constrained U(ij) Components(s) for C21#       .          6 Check
PLAT218_ALERT_3_C Constrained U(ij) Components(s) for C22#       .          6 Check
PLAT218_ALERT_3_C Constrained U(ij) Components(s) for N7#        .          6 Check
PLAT218_ALERT_3_C Constrained U(ij) Components(s) for O11#       .          6 Check
PLAT218_ALERT_3_C Constrained U(ij) Components(s) for C23#       .          6 Check
PLAT218_ALERT_3_C Constrained U(ij) Components(s) for C24#       .          6 Check
PLAT218_ALERT_3_C Constrained U(ij) Components(s) for C25#       .          6 Check
PLAT218_ALERT_3_C Constrained U(ij) Components(s) for C26#       .          6 Check
PLAT218_ALERT_3_C Constrained U(ij) Components(s) for C27#       .          6 Check
PLAT218_ALERT_3_C Constrained U(ij) Components(s) for N8#        .          6 Check
PLAT218_ALERT_3_C Constrained U(ij) Components(s) for O12#       .          6 Check
PLAT218_ALERT_3_C Constrained U(ij) Components(s) for C28#       .          6 Check
PLAT218_ALERT_3_C Constrained U(ij) Components(s) for C29#       .          6 Check
PLAT218_ALERT_3_C Constrained U(ij) Components(s) for O13#       .          6 Check
PLAT218_ALERT_3_C Constrained U(ij) Components(s) for C30#       .          6 Check
PLAT218_ALERT_3_C Constrained U(ij) Components(s) for C31#       .          6 Check
PLAT218_ALERT_3_C Constrained U(ij) Components(s) for O14#       .          6 Check
PLAT218_ALERT_3_C Constrained U(ij) Components(s) for C32#       .          6 Check
PLAT218_ALERT_3_C Constrained U(ij) Components(s) for N9#        .          6 Check
PLAT218_ALERT_3_C Constrained U(ij) Components(s) for C33#       .          6 Check
PLAT218_ALERT_3_C Constrained U(ij) Components(s) for C34#       .          6 Check
PLAT218_ALERT_3_C Constrained U(ij) Components(s) for C35#       .          6 Check
PLAT218_ALERT_3_C Constrained U(ij) Components(s) for C36#       .          6 Check
```

```
PLAT218_ALERT_3_C Constrained U(ij) Components(s) for C37#       .          6 Check
PLAT218_ALERT_3_C Constrained U(ij) Components(s) for C38#       .          6 Check
PLAT218_ALERT_3_C Constrained U(ij) Components(s) for C39#       .          6 Check
PLAT218_ALERT_3_C Constrained U(ij) Components(s) for N10#       .          6 Check
PLAT218_ALERT_3_C Constrained U(ij) Components(s) for C40#       .          6 Check
PLAT218_ALERT_3_C Constrained U(ij) Components(s) for O15#       .          6 Check
PLAT218_ALERT_3_C Constrained U(ij) Components(s) for C41#       .          6 Check
PLAT218_ALERT_3_C Constrained U(ij) Components(s) for C42#       .          6 Check
PLAT218_ALERT_3_C Constrained U(ij) Components(s) for O16#       .          6 Check
PLAT218_ALERT_3_C Constrained U(ij) Components(s) for C43#       .          6 Check
PLAT218_ALERT_3_C Constrained U(ij) Components(s) for C44#       .          6 Check
PLAT218_ALERT_3_C Constrained U(ij) Components(s) for C45#       .          6 Check
PLAT218_ALERT_3_C Constrained U(ij) Components(s) for C46#       .          6 Check
PLAT218_ALERT_3_C Constrained U(ij) Components(s) for N11#       .          6 Check
PLAT218_ALERT_3_C Constrained U(ij) Components(s) for C47#       .          6 Check
PLAT218_ALERT_3_C Constrained U(ij) Components(s) for C48#       .          6 Check
PLAT218_ALERT_3_C Constrained U(ij) Components(s) for C49#       .          6 Check
PLAT218_ALERT_3_C Constrained U(ij) Components(s) for C50#       .          6 Check
PLAT218_ALERT_3_C Constrained U(ij) Components(s) for O17#       .          6 Check
PLAT218_ALERT_3_C Constrained U(ij) Components(s) for C51#       .          6 Check
PLAT218_ALERT_3_C Constrained U(ij) Components(s) for N12#       .          6 Check
PLAT218_ALERT_3_C Constrained U(ij) Components(s) for C52#       .          6 Check
PLAT218_ALERT_3_C Constrained U(ij) Components(s) for C53#       .          6 Check
PLAT218_ALERT_3_C Constrained U(ij) Components(s) for C54#       .          6 Check
PLAT218_ALERT_3_C Constrained U(ij) Components(s) for C55#       .          6 Check
PLAT218_ALERT_3_C Constrained U(ij) Components(s) for C56#       .          6 Check
PLAT218_ALERT_3_C Constrained U(ij) Components(s) for O18#       .          6 Check
PLAT218_ALERT_3_C Constrained U(ij) Components(s) for C57#       .          6 Check
PLAT218_ALERT_3_C Constrained U(ij) Components(s) for N13#       .          6 Check
PLAT218_ALERT_3_C Constrained U(ij) Components(s) for C58#       .          6 Check
PLAT218_ALERT_3_C Constrained U(ij) Components(s) for O19#       .          6 Check
PLAT218_ALERT_3_C Constrained U(ij) Components(s) for N14#       .          6 Check
PLAT218_ALERT_3_C Constrained U(ij) Components(s) for C59#       .          6 Check
PLAT218_ALERT_3_C Constrained U(ij) Components(s) for C60#       .          6 Check
PLAT218_ALERT_3_C Constrained U(ij) Components(s) for N15#       .          6 Check
PLAT218_ALERT_3_C Constrained U(ij) Components(s) for C61#       .          6 Check
PLAT218_ALERT_3_C Constrained U(ij) Components(s) for C62#       .          6 Check
PLAT218_ALERT_3_C Constrained U(ij) Components(s) for C63#       .          6 Check
PLAT218_ALERT_3_C Constrained U(ij) Components(s) for N16#       .          6 Check
PLAT218_ALERT_3_C Constrained U(ij) Components(s) for C64#       .          6 Check
PLAT218_ALERT_3_C Constrained U(ij) Components(s) for C65#       .          6 Check
PLAT218_ALERT_3_C Constrained U(ij) Components(s) for N17#       .          6 Check
PLAT218_ALERT_3_C Constrained U(ij) Components(s) for N18#       .          6 Check
PLAT218_ALERT_3_C Constrained U(ij) Components(s) for C66#       .          6 Check
PLAT218_ALERT_3_C Constrained U(ij) Components(s) for O20#       .          6 Check
PLAT218_ALERT_3_C Constrained U(ij) Components(s) for N19#       .          6 Check
PLAT218_ALERT_3_C Constrained U(ij) Components(s) for C67#       .          6 Check
PLAT218_ALERT_3_C Constrained U(ij) Components(s) for O21#       .          6 Check
PLAT218_ALERT_3_C Constrained U(ij) Components(s) for C68#       .          6 Check
PLAT218_ALERT_3_C Constrained U(ij) Components(s) for C69#       .          6 Check
PLAT218_ALERT_3_C Constrained U(ij) Components(s) for C70#       .          6 Check
PLAT218_ALERT_3_C Constrained U(ij) Components(s) for C71#       .          6 Check
PLAT218_ALERT_3_C Constrained U(ij) Components(s) for N20#       .          6 Check
PLAT218_ALERT_3_C Constrained U(ij) Components(s) for C72#       .          6 Check
PLAT218_ALERT_3_C Constrained U(ij) Components(s) for N21#       .          6 Check
PLAT218_ALERT_3_C Constrained U(ij) Components(s) for N22#       .          6 Check
PLAT218_ALERT_3_C Constrained U(ij) Components(s) for N23#       .          6 Check
```

```
PLAT218_ALERT_3_C Constrained U(ij) Components(s) for C73#        .          6 Check
PLAT218_ALERT_3_C Constrained U(ij) Components(s) for C74#        .          6 Check
PLAT218_ALERT_3_C Constrained U(ij) Components(s) for O22#        .          6 Check
PLAT218_ALERT_3_C Constrained U(ij) Components(s) for C75#        .          6 Check
PLAT218_ALERT_3_C Constrained U(ij) Components(s) for O23#        .          6 Check
PLAT218_ALERT_3_C Constrained U(ij) Components(s) for N24#        .          6 Check
PLAT218_ALERT_3_C Constrained U(ij) Components(s) for C76#        .          6 Check
PLAT218_ALERT_3_C Constrained U(ij) Components(s) for N25#        .          6 Check
PLAT218_ALERT_3_C Constrained U(ij) Components(s) for O24#        .          6 Check
PLAT218_ALERT_3_C Constrained U(ij) Components(s) for C77#        .          6 Check
PLAT218_ALERT_3_C Constrained U(ij) Components(s) for C78#        .          6 Check
PLAT218_ALERT_3_C Constrained U(ij) Components(s) for O25#        .          6 Check
PLAT218_ALERT_3_C Constrained U(ij) Components(s) for C79#        .          6 Check
PLAT218_ALERT_3_C Constrained U(ij) Components(s) for N26#        .          6 Check
PLAT218_ALERT_3_C Constrained U(ij) Components(s) for O26#        .          6 Check
PLAT218_ALERT_3_C Constrained U(ij) Components(s) for C80#        .          6 Check
PLAT218_ALERT_3_C Constrained U(ij) Components(s) for C81#        .          6 Check
PLAT218_ALERT_3_C Constrained U(ij) Components(s) for C82#        .          6 Check
PLAT218_ALERT_3_C Constrained U(ij) Components(s) for C83#        .          6 Check
PLAT218_ALERT_3_C Constrained U(ij) Components(s) for C84#        .          6 Check
PLAT218_ALERT_3_C Constrained U(ij) Components(s) for C85#        .          6 Check
PLAT218_ALERT_3_C Constrained U(ij) Components(s) for C86#        .          6 Check
PLAT218_ALERT_3_C Constrained U(ij) Components(s) for C87#        .          6 Check
PLAT218_ALERT_3_C Constrained U(ij) Components(s) for C88#        .          6 Check
PLAT218_ALERT_3_C Constrained U(ij) Components(s) for N27#        .          6 Check
PLAT218_ALERT_3_C Constrained U(ij) Components(s) for C89#        .          6 Check
PLAT218_ALERT_3_C Constrained U(ij) Components(s) for C90#        .          6 Check
PLAT218_ALERT_3_C Constrained U(ij) Components(s) for C91#        .          6 Check
PLAT218_ALERT_3_C Constrained U(ij) Components(s) for C92#        .          6 Check
PLAT218_ALERT_3_C Constrained U(ij) Components(s) for C93#        .          6 Check
PLAT218_ALERT_3_C Constrained U(ij) Components(s) for C94#        .          6 Check
PLAT218_ALERT_3_C Constrained U(ij) Components(s) for C95#        .          6 Check
PLAT218_ALERT_3_C Constrained U(ij) Components(s) for C96#        .          6 Check
PLAT218_ALERT_3_C Constrained U(ij) Components(s) for C97#        .          6 Check
PLAT218_ALERT_3_C Constrained U(ij) Components(s) for O27#        .          6 Check
PLAT218_ALERT_3_C Constrained U(ij) Components(s) for N28#        .          6 Check
PLAT218_ALERT_3_C Constrained U(ij) Components(s) for C98#        .          6 Check
PLAT218_ALERT_3_C Constrained U(ij) Components(s) for C99#        .          6 Check
PLAT218_ALERT_3_C Constrained U(ij) Components(s) for C100#       .          6 Check
PLAT218_ALERT_3_C Constrained U(ij) Components(s) for O28#        .          6 Check
PLAT218_ALERT_3_C Constrained U(ij) Components(s) for N29#        .          6 Check
PLAT218_ALERT_3_C Constrained U(ij) Components(s) for O29#        .          6 Check
PLAT218_ALERT_3_C Constrained U(ij) Components(s) for C101#       .          6 Check
PLAT218_ALERT_3_C Constrained U(ij) Components(s) for N30#        .          6 Check
PLAT218_ALERT_3_C Constrained U(ij) Components(s) for C102#       .          6 Check
PLAT218_ALERT_3_C Constrained U(ij) Components(s) for C103#       .          6 Check
PLAT218_ALERT_3_C Constrained U(ij) Components(s) for C104#       .          6 Check
PLAT218_ALERT_3_C Constrained U(ij) Components(s) for C105#       .          6 Check
PLAT218_ALERT_3_C Constrained U(ij) Components(s) for C106#       .          6 Check
PLAT218_ALERT_3_C Constrained U(ij) Components(s) for O30#        .          6 Check
PLAT218_ALERT_3_C Constrained U(ij) Components(s) for N31#        .          6 Check
PLAT218_ALERT_3_C Constrained U(ij) Components(s) for C107#       .          6 Check
PLAT218_ALERT_3_C Constrained U(ij) Components(s) for S3#         .          6 Check
PLAT218_ALERT_3_C Constrained U(ij) Components(s) for C108#       .          6 Check
PLAT218_ALERT_3_C Constrained U(ij) Components(s) for O31#        .          6 Check
PLAT218_ALERT_3_C Constrained U(ij) Components(s) for C109#       .          6 Check
PLAT218_ALERT_3_C Constrained U(ij) Components(s) for N32#        .          6 Check
```

```
PLAT218_ALERT_3_C Constrained U(ij) Components(s) for C110#      .          6 Check
PLAT218_ALERT_3_C Constrained U(ij) Components(s) for C111#      .          6 Check
PLAT218_ALERT_3_C Constrained U(ij) Components(s) for C112#      .          6 Check
PLAT218_ALERT_3_C Constrained U(ij) Components(s) for C113#      .          6 Check
PLAT218_ALERT_3_C Constrained U(ij) Components(s) for N33#       .          6 Check
PLAT218_ALERT_3_C Constrained U(ij) Components(s) for C114#      .          6 Check
PLAT218_ALERT_3_C Constrained U(ij) Components(s) for N34#       .          6 Check
PLAT218_ALERT_3_C Constrained U(ij) Components(s) for N35#       .          6 Check
PLAT218_ALERT_3_C Constrained U(ij) Components(s) for C115#      .          6 Check
PLAT218_ALERT_3_C Constrained U(ij) Components(s) for O32#       .          6 Check
PLAT218_ALERT_3_C Constrained U(ij) Components(s) for N36#       .          6 Check
PLAT218_ALERT_3_C Constrained U(ij) Components(s) for C116#      .          6 Check
PLAT218_ALERT_3_C Constrained U(ij) Components(s) for C117#      .          6 Check
PLAT218_ALERT_3_C Constrained U(ij) Components(s) for C118#      .          6 Check
PLAT218_ALERT_3_C Constrained U(ij) Components(s) for C119#      .          6 Check
PLAT218_ALERT_3_C Constrained U(ij) Components(s) for C120#      .          6 Check
PLAT218_ALERT_3_C Constrained U(ij) Components(s) for O33#       .          6 Check
PLAT218_ALERT_3_C Constrained U(ij) Components(s) for C121#      .          6 Check
PLAT218_ALERT_3_C Constrained U(ij) Components(s) for N37#       .          6 Check
PLAT218_ALERT_3_C Constrained U(ij) Components(s) for C122#      .          6 Check
PLAT218_ALERT_3_C Constrained U(ij) Components(s) for C123#      .          6 Check
PLAT218_ALERT_3_C Constrained U(ij) Components(s) for C124#      .          6 Check
PLAT218_ALERT_3_C Constrained U(ij) Components(s) for C125#      .          6 Check
PLAT218_ALERT_3_C Constrained U(ij) Components(s) for C126#      .          6 Check
PLAT218_ALERT_3_C Constrained U(ij) Components(s) for O34#       .          6 Check
PLAT218_ALERT_3_C Constrained U(ij) Components(s) for N38#       .          6 Check
PLAT218_ALERT_3_C Constrained U(ij) Components(s) for C127#      .          6 Check
PLAT218_ALERT_3_C Constrained U(ij) Components(s) for C128#      .          6 Check
PLAT218_ALERT_3_C Constrained U(ij) Components(s) for C129#      .          6 Check
PLAT218_ALERT_3_C Constrained U(ij) Components(s) for C130#      .          6 Check
PLAT218_ALERT_3_C Constrained U(ij) Components(s) for O35#       .          6 Check
PLAT218_ALERT_3_C Constrained U(ij) Components(s) for C131#      .          6 Check
PLAT218_ALERT_3_C Constrained U(ij) Components(s) for C132#      .          6 Check
PLAT218_ALERT_3_C Constrained U(ij) Components(s) for N39#       .          6 Check
PLAT218_ALERT_3_C Constrained U(ij) Components(s) for C133#      .          6 Check
PLAT218_ALERT_3_C Constrained U(ij) Components(s) for O36#       .          6 Check
PLAT218_ALERT_3_C Constrained U(ij) Components(s) for N40#       .          6 Check
PLAT218_ALERT_3_C Constrained U(ij) Components(s) for C134#      .          6 Check
PLAT218_ALERT_3_C Constrained U(ij) Components(s) for C135#      .          6 Check
PLAT218_ALERT_3_C Constrained U(ij) Components(s) for O37#       .          6 Check
PLAT218_ALERT_3_C Constrained U(ij) Components(s) for C136#      .          6 Check
PLAT218_ALERT_3_C Constrained U(ij) Components(s) for C137#      .          6 Check
PLAT218_ALERT_3_C Constrained U(ij) Components(s) for O38#       .          6 Check
PLAT218_ALERT_3_C Constrained U(ij) Components(s) for N41#       .          6 Check
PLAT218_ALERT_3_C Constrained U(ij) Components(s) for C138#      .          6 Check
PLAT218_ALERT_3_C Constrained U(ij) Components(s) for C139#      .          6 Check
PLAT218_ALERT_3_C Constrained U(ij) Components(s) for O39#       .          6 Check
PLAT218_ALERT_3_C Constrained U(ij) Components(s) for O40#       .          6 Check
PLAT218_ALERT_3_C Constrained U(ij) Components(s) for C140#      .          6 Check
PLAT218_ALERT_3_C Constrained U(ij) Components(s) for N42#       .          6 Check
PLAT218_ALERT_3_C Constrained U(ij) Components(s) for C141#      .          6 Check
PLAT218_ALERT_3_C Constrained U(ij) Components(s) for C142#      .          6 Check
PLAT218_ALERT_3_C Constrained U(ij) Components(s) for C143#      .          6 Check
PLAT218_ALERT_3_C Constrained U(ij) Components(s) for C144#      .          6 Check
PLAT218_ALERT_3_C Constrained U(ij) Components(s) for C145#      .          6 Check
PLAT218_ALERT_3_C Constrained U(ij) Components(s) for O41#       .          6 Check
PLAT218_ALERT_3_C Constrained U(ij) Components(s) for N43#       .          6 Check
```

```
PLAT218_ALERT_3_C Constrained U(ij) Components(s) for C146#       .            6 Check
PLAT218_ALERT_3_C Constrained U(ij) Components(s) for C147#       .            6 Check
PLAT218_ALERT_3_C Constrained U(ij) Components(s) for O42#        .            6 Check
PLAT218_ALERT_3_C Constrained U(ij) Components(s) for O43#        .            6 Check
PLAT218_ALERT_3_C Constrained U(ij) Components(s) for C148#       .            6 Check
PLAT218_ALERT_3_C Constrained U(ij) Components(s) for N44#        .            6 Check
PLAT218_ALERT_3_C Constrained U(ij) Components(s) for C149#       .            6 Check
PLAT218_ALERT_3_C Constrained U(ij) Components(s) for C150#       .            6 Check
PLAT218_ALERT_3_C Constrained U(ij) Components(s) for C151#       .            6 Check
PLAT218_ALERT_3_C Constrained U(ij) Components(s) for C152#       .            6 Check
PLAT218_ALERT_3_C Constrained U(ij) Components(s) for O44#        .            6 Check
PLAT218_ALERT_3_C Constrained U(ij) Components(s) for O45#        .            6 Check
PLAT218_ALERT_3_C Constrained U(ij) Components(s) for C153#       .            6 Check
PLAT218_ALERT_3_C Constrained U(ij) Components(s) for O46#        .            6 Check
PLAT218_ALERT_3_C Constrained U(ij) Components(s) for N45#        .            6 Check
PLAT218_ALERT_3_C Constrained U(ij) Components(s) for C154#       .            6 Check
PLAT218_ALERT_3_C Constrained U(ij) Components(s) for C155#       .            6 Check
PLAT218_ALERT_3_C Constrained U(ij) Components(s) for C156#       .            6 Check
PLAT218_ALERT_3_C Constrained U(ij) Components(s) for O47#        .            6 Check
PLAT218_ALERT_3_C Constrained U(ij) Components(s) for N46#        .            6 Check
PLAT218_ALERT_3_C Constrained U(ij) Components(s) for C157#       .            6 Check
PLAT218_ALERT_3_C Constrained U(ij) Components(s) for C158#       .            6 Check
PLAT218_ALERT_3_C Constrained U(ij) Components(s) for C159#       .            6 Check
PLAT218_ALERT_3_C Constrained U(ij) Components(s) for C160#       .            6 Check
PLAT218_ALERT_3_C Constrained U(ij) Components(s) for C161#       .            6 Check
PLAT218_ALERT_3_C Constrained U(ij) Components(s) for C162#       .            6 Check
PLAT218_ALERT_3_C Constrained U(ij) Components(s) for O48#        .            6 Check
PLAT218_ALERT_3_C Constrained U(ij) Components(s) for N47#        .            6 Check
PLAT218_ALERT_3_C Constrained U(ij) Components(s) for C163#       .            6 Check
PLAT218_ALERT_3_C Constrained U(ij) Components(s) for C164#       .            6 Check
PLAT218_ALERT_3_C Constrained U(ij) Components(s) for C165#       .            6 Check
PLAT218_ALERT_3_C Constrained U(ij) Components(s) for C166#       .            6 Check
PLAT218_ALERT_3_C Constrained U(ij) Components(s) for C167#       .            6 Check
PLAT218_ALERT_3_C Constrained U(ij) Components(s) for C168#       .            6 Check
PLAT218_ALERT_3_C Constrained U(ij) Components(s) for O49#        .            6 Check
PLAT218_ALERT_3_C Constrained U(ij) Components(s) for N48#        .            6 Check
PLAT218_ALERT_3_C Constrained U(ij) Components(s) for C169#       .            6 Check
PLAT218_ALERT_3_C Constrained U(ij) Components(s) for C170#       .            6 Check
PLAT218_ALERT_3_C Constrained U(ij) Components(s) for C171#       .            6 Check
PLAT218_ALERT_3_C Constrained U(ij) Components(s) for C172#       .            6 Check
PLAT218_ALERT_3_C Constrained U(ij) Components(s) for C173#       .            6 Check
PLAT218_ALERT_3_C Constrained U(ij) Components(s) for C174#       .            6 Check
PLAT218_ALERT_3_C Constrained U(ij) Components(s) for O50#        .            6 Check
PLAT218_ALERT_3_C Constrained U(ij) Components(s) for N49#        .            6 Check
PLAT218_ALERT_3_C Constrained U(ij) Components(s) for C175#       .            6 Check
PLAT218_ALERT_3_C Constrained U(ij) Components(s) for S4#         .            6 Check
PLAT218_ALERT_3_C Constrained U(ij) Components(s) for C176#       .            6 Check
PLAT218_ALERT_3_C Constrained U(ij) Components(s) for O51#        .            6 Check
PLAT218_ALERT_3_C Constrained U(ij) Components(s) for C177#       .            6 Check
PLAT218_ALERT_3_C Constrained U(ij) Components(s) for N50#        .            6 Check
PLAT218_ALERT_3_C Constrained U(ij) Components(s) for C178#       .            6 Check
PLAT218_ALERT_3_C Constrained U(ij) Components(s) for C179#       .            6 Check
PLAT218_ALERT_3_C Constrained U(ij) Components(s) for C180#       .            6 Check
PLAT218_ALERT_3_C Constrained U(ij) Components(s) for O52#        .            6 Check
PLAT218_ALERT_3_C Constrained U(ij) Components(s) for N51#        .            6 Check
PLAT218_ALERT_3_C Constrained U(ij) Components(s) for C181#       .            6 Check
PLAT218_ALERT_3_C Constrained U(ij) Components(s) for C182#       .            6 Check
```

```
PLAT218_ALERT_3_C Constrained U(ij) Components(s) for O53#       .          6 Check
PLAT218_ALERT_3_C Constrained U(ij) Components(s) for C183#      .          6 Check
PLAT218_ALERT_3_C Constrained U(ij) Components(s) for C184#      .          6 Check
PLAT218_ALERT_3_C Constrained U(ij) Components(s) for O54#       .          6 Check
PLAT218_ALERT_3_C Constrained U(ij) Components(s) for N52#       .          6 Check
PLAT218_ALERT_3_C Constrained U(ij) Components(s) for C185#      .          6 Check
PLAT218_ALERT_3_C Constrained U(ij) Components(s) for C186#      .          6 Check
PLAT218_ALERT_3_C Constrained U(ij) Components(s) for C187#      .          6 Check
PLAT218_ALERT_3_C Constrained U(ij) Components(s) for C188#      .          6 Check
PLAT218_ALERT_3_C Constrained U(ij) Components(s) for C189#      .          6 Check
PLAT218_ALERT_3_C Constrained U(ij) Components(s) for C190#      .          6 Check
PLAT218_ALERT_3_C Constrained U(ij) Components(s) for C191#      .          6 Check
PLAT218_ALERT_3_C Constrained U(ij) Components(s) for C192#      .          6 Check
PLAT218_ALERT_3_C Constrained U(ij) Components(s) for O55#       .          6 Check
PLAT218_ALERT_3_C Constrained U(ij) Components(s) for C193#      .          6 Check
PLAT218_ALERT_3_C Constrained U(ij) Components(s) for O56#       .          6 Check
PLAT218_ALERT_3_C Constrained U(ij) Components(s) for N53#       .          6 Check
PLAT218_ALERT_3_C Constrained U(ij) Components(s) for C194#      .          6 Check
PLAT218_ALERT_3_C Constrained U(ij) Components(s) for C195#      .          6 Check
PLAT218_ALERT_3_C Constrained U(ij) Components(s) for C196#      .          6 Check
PLAT218_ALERT_3_C Constrained U(ij) Components(s) for C197#      .          6 Check
PLAT218_ALERT_3_C Constrained U(ij) Components(s) for C198#      .          6 Check
PLAT218_ALERT_3_C Constrained U(ij) Components(s) for C199#      .          6 Check
PLAT218_ALERT_3_C Constrained U(ij) Components(s) for C200#      .          6 Check
PLAT218_ALERT_3_C Constrained U(ij) Components(s) for C201#      .          6 Check
PLAT218_ALERT_3_C Constrained U(ij) Components(s) for O57#       .          6 Check
PLAT218_ALERT_3_C Constrained U(ij) Components(s) for C202#      .          6 Check
PLAT218_ALERT_3_C Constrained U(ij) Components(s) for O58#       .          6 Check
PLAT218_ALERT_3_C Constrained U(ij) Components(s) for N54#       .          6 Check
PLAT218_ALERT_3_C Constrained U(ij) Components(s) for C203#      .          6 Check
PLAT218_ALERT_3_C Constrained U(ij) Components(s) for C204#      .          6 Check
PLAT218_ALERT_3_C Constrained U(ij) Components(s) for C205#      .          6 Check
PLAT218_ALERT_3_C Constrained U(ij) Components(s) for C206#      .          6 Check
PLAT218_ALERT_3_C Constrained U(ij) Components(s) for C207#      .          6 Check
PLAT218_ALERT_3_C Constrained U(ij) Components(s) for C208#      .          6 Check
PLAT218_ALERT_3_C Constrained U(ij) Components(s) for C209#      .          6 Check
PLAT218_ALERT_3_C Constrained U(ij) Components(s) for C210#      .          6 Check
PLAT218_ALERT_3_C Constrained U(ij) Components(s) for O59#       .          6 Check
PLAT218_ALERT_3_C Constrained U(ij) Components(s) for C211#      .          6 Check
PLAT218_ALERT_3_C Constrained U(ij) Components(s) for O60#       .          6 Check
PLAT218_ALERT_3_C Constrained U(ij) Components(s) for N55#       .          6 Check
PLAT218_ALERT_3_C Constrained U(ij) Components(s) for C212#      .          6 Check
PLAT218_ALERT_3_C Constrained U(ij) Components(s) for C213#      .          6 Check
PLAT218_ALERT_3_C Constrained U(ij) Components(s) for O61#       .          6 Check
PLAT218_ALERT_3_C Constrained U(ij) Components(s) for C214#      .          6 Check
PLAT218_ALERT_3_C Constrained U(ij) Components(s) for C215#      .          6 Check
PLAT218_ALERT_3_C Constrained U(ij) Components(s) for O62#       .          6 Check
PLAT218_ALERT_3_C Constrained U(ij) Components(s) for N56#       .          6 Check
PLAT218_ALERT_3_C Constrained U(ij) Components(s) for C216#      .          6 Check
PLAT218_ALERT_3_C Constrained U(ij) Components(s) for O63#       .          6 Check
PLAT218_ALERT_3_C Constrained U(ij) Components(s) for C217#      .          6 Check
PLAT218_ALERT_3_C Constrained U(ij) Components(s) for N57#       .          6 Check
PLAT218_ALERT_3_C Constrained U(ij) Components(s) for C218#      .          6 Check
PLAT218_ALERT_3_C Constrained U(ij) Components(s) for C219#      .          6 Check
PLAT218_ALERT_3_C Constrained U(ij) Components(s) for S5#        .          6 Check
PLAT218_ALERT_3_C Constrained U(ij) Components(s) for C220#      .          6 Check
PLAT218_ALERT_3_C Constrained U(ij) Components(s) for O64#       .          6 Check
```

```
PLAT218_ALERT_3_C Constrained U(ij) Components(s) for N58#        .           6 Check
PLAT218_ALERT_3_C Constrained U(ij) Components(s) for C221#       .           6 Check
PLAT218_ALERT_3_C Constrained U(ij) Components(s) for C222#       .           6 Check
PLAT218_ALERT_3_C Constrained U(ij) Components(s) for C223#       .           6 Check
PLAT218_ALERT_3_C Constrained U(ij) Components(s) for C224#       .           6 Check
PLAT218_ALERT_3_C Constrained U(ij) Components(s) for C225#       .           6 Check
PLAT218_ALERT_3_C Constrained U(ij) Components(s) for C226#       .           6 Check
PLAT218_ALERT_3_C Constrained U(ij) Components(s) for O65#        .           6 Check
PLAT218_ALERT_3_C Constrained U(ij) Components(s) for N59#        .           6 Check
PLAT218_ALERT_3_C Constrained U(ij) Components(s) for C227#       .           6 Check
PLAT218_ALERT_3_C Constrained U(ij) Components(s) for C228#       .           6 Check
PLAT218_ALERT_3_C Constrained U(ij) Components(s) for C229#       .           6 Check
PLAT218_ALERT_3_C Constrained U(ij) Components(s) for C230#       .           6 Check
PLAT218_ALERT_3_C Constrained U(ij) Components(s) for C231#       .           6 Check
PLAT218_ALERT_3_C Constrained U(ij) Components(s) for O66#        .           6 Check
PLAT218_ALERT_3_C Constrained U(ij) Components(s) for C232#       .           6 Check
PLAT218_ALERT_3_C Constrained U(ij) Components(s) for C233#       .           6 Check
PLAT218_ALERT_3_C Constrained U(ij) Components(s) for N60#        .           6 Check
PLAT218_ALERT_3_C Constrained U(ij) Components(s) for C234#       .           6 Check
PLAT218_ALERT_3_C Constrained U(ij) Components(s) for C235#       .           6 Check
PLAT218_ALERT_3_C Constrained U(ij) Components(s) for C236#       .           6 Check
PLAT218_ALERT_3_C Constrained U(ij) Components(s) for C237#       .           6 Check
PLAT218_ALERT_3_C Constrained U(ij) Components(s) for C238#       .           6 Check
PLAT218_ALERT_3_C Constrained U(ij) Components(s) for C239#       .           6 Check
PLAT218_ALERT_3_C Constrained U(ij) Components(s) for O67#        .           6 Check
PLAT218_ALERT_3_C Constrained U(ij) Components(s) for N61#        .           6 Check
PLAT218_ALERT_3_C Constrained U(ij) Components(s) for C240#       .           6 Check
PLAT218_ALERT_3_C Constrained U(ij) Components(s) for C241#       .           6 Check
PLAT218_ALERT_3_C Constrained U(ij) Components(s) for C242#       .           6 Check
PLAT218_ALERT_3_C Constrained U(ij) Components(s) for C243#       .           6 Check
PLAT218_ALERT_3_C Constrained U(ij) Components(s) for C244#       .           6 Check
PLAT218_ALERT_3_C Constrained U(ij) Components(s) for O68#        .           6 Check
PLAT218_ALERT_3_C Constrained U(ij) Components(s) for N62#        .           6 Check
PLAT218_ALERT_3_C Constrained U(ij) Components(s) for C245#       .           6 Check
PLAT218_ALERT_3_C Constrained U(ij) Components(s) for C246#       .           6 Check
PLAT218_ALERT_3_C Constrained U(ij) Components(s) for O69#        .           6 Check
PLAT218_ALERT_3_C Constrained U(ij) Components(s) for N63#        .           6 Check
PLAT218_ALERT_3_C Constrained U(ij) Components(s) for C247#       .           6 Check
PLAT218_ALERT_3_C Constrained U(ij) Components(s) for C248#       .           6 Check
PLAT218_ALERT_3_C Constrained U(ij) Components(s) for O70#        .           6 Check
PLAT218_ALERT_3_C Constrained U(ij) Components(s) for N64#        .           6 Check
PLAT218_ALERT_3_C Constrained U(ij) Components(s) for C249#       .           6 Check
PLAT218_ALERT_3_C Constrained U(ij) Components(s) for C250#       .           6 Check
PLAT218_ALERT_3_C Constrained U(ij) Components(s) for C251#       .           6 Check
PLAT218_ALERT_3_C Constrained U(ij) Components(s) for O71#        .           6 Check
PLAT218_ALERT_3_C Constrained U(ij) Components(s) for N65#        .           6 Check
PLAT218_ALERT_3_C Constrained U(ij) Components(s) for C252#       .           6 Check
PLAT218_ALERT_3_C Constrained U(ij) Components(s) for C253#       .           6 Check
PLAT218_ALERT_3_C Constrained U(ij) Components(s) for O72#        .           6 Check
PLAT218_ALERT_3_C Constrained U(ij) Components(s) for C254#       .           6 Check
PLAT218_ALERT_3_C Constrained U(ij) Components(s) for C255#       .           6 Check
PLAT218_ALERT_3_C Constrained U(ij) Components(s) for O73#        .           6 Check
PLAT218_ALERT_3_C Constrained U(ij) Components(s) for N66#        .           6 Check
PLAT218_ALERT_3_C Constrained U(ij) Components(s) for C256#       .           6 Check
PLAT218_ALERT_3_C Constrained U(ij) Components(s) for C257#       .           6 Check
PLAT218_ALERT_3_C Constrained U(ij) Components(s) for C258#       .           6 Check
PLAT218_ALERT_3_C Constrained U(ij) Components(s) for O74#        .           6 Check
```

```
PLAT218_ALERT_3_C Constrained U(ij) Components(s) for C259#      .          6 Check
PLAT218_ALERT_3_C Constrained U(ij) Components(s) for O75#       .          6 Check
PLAT218_ALERT_3_C Constrained U(ij) Components(s) for N67#       .          6 Check
PLAT218_ALERT_3_C Constrained U(ij) Components(s) for C260#      .          6 Check
PLAT218_ALERT_3_C Constrained U(ij) Components(s) for C261#      .          6 Check
PLAT218_ALERT_3_C Constrained U(ij) Components(s) for S6#        .          6 Check
PLAT218_ALERT_3_C Constrained U(ij) Components(s) for C262#      .          6 Check
PLAT218_ALERT_3_C Constrained U(ij) Components(s) for O76#       .          6 Check
PLAT218_ALERT_3_C Constrained U(ij) Components(s) for N68#       .          6 Check
PLAT218_ALERT_3_C Constrained U(ij) Components(s) for C263#      .          6 Check
PLAT218_ALERT_3_C Constrained U(ij) Components(s) for C264#      .          6 Check
PLAT218_ALERT_3_C Constrained U(ij) Components(s) for C265#      .          6 Check
PLAT218_ALERT_3_C Constrained U(ij) Components(s) for C266#      .          6 Check
PLAT218_ALERT_3_C Constrained U(ij) Components(s) for C267#      .          6 Check
PLAT218_ALERT_3_C Constrained U(ij) Components(s) for O77#       .          6 Check
PLAT218_ALERT_3_C Constrained U(ij) Components(s) for N69#       .          6 Check
PLAT218_ALERT_3_C Constrained U(ij) Components(s) for C268#      .          6 Check
PLAT218_ALERT_3_C Constrained U(ij) Components(s) for C269#      .          6 Check
PLAT218_ALERT_3_C Constrained U(ij) Components(s) for O78#       .          6 Check
PLAT218_ALERT_3_C Constrained U(ij) Components(s) for N70#       .          6 Check
PLAT218_ALERT_3_C Constrained U(ij) Components(s) for C270#      .          6 Check
PLAT218_ALERT_3_C Constrained U(ij) Components(s) for C271#      .          6 Check
PLAT218_ALERT_3_C Constrained U(ij) Components(s) for C272#      .          6 Check
PLAT218_ALERT_3_C Constrained U(ij) Components(s) for O79#       .          6 Check
PLAT218_ALERT_3_C Constrained U(ij) Components(s) for O80#       .          6 Check
PLAT218_ALERT_3_C Constrained U(ij) Components(s) for C273#      .          6 Check
PLAT218_ALERT_3_C Constrained U(ij) Components(s) for O81#       .          6 Check
PLAT218_ALERT_3_C Constrained U(ij) Components(s) for N71#       .          6 Check
PLAT218_ALERT_3_C Constrained U(ij) Components(s) for C274#      .          6 Check
PLAT218_ALERT_3_C Constrained U(ij) Components(s) for C275#      .          6 Check
PLAT218_ALERT_3_C Constrained U(ij) Components(s) for O82#       .          6 Check
PLAT218_ALERT_3_C Constrained U(ij) Components(s) for O83#       .          6 Check
PLAT218_ALERT_3_C Constrained U(ij) Components(s) for C276#      .          6 Check
PLAT218_ALERT_3_C Constrained U(ij) Components(s) for C277#      .          6 Check
PLAT218_ALERT_3_C Constrained U(ij) Components(s) for O84#       .          6 Check
PLAT218_ALERT_3_C Constrained U(ij) Components(s) for N72#       .          6 Check
PLAT218_ALERT_3_C Constrained U(ij) Components(s) for C278#      .          6 Check
PLAT218_ALERT_3_C Constrained U(ij) Components(s) for C279#      .          6 Check
PLAT218_ALERT_3_C Constrained U(ij) Components(s) for C280#      .          6 Check
PLAT218_ALERT_3_C Constrained U(ij) Components(s) for C281#      .          6 Check
PLAT218_ALERT_3_C Constrained U(ij) Components(s) for C282#      .          6 Check
PLAT218_ALERT_3_C Constrained U(ij) Components(s) for C283#      .          6 Check
PLAT218_ALERT_3_C Constrained U(ij) Components(s) for C284#      .          6 Check
PLAT218_ALERT_3_C Constrained U(ij) Components(s) for C285#      .          6 Check
PLAT218_ALERT_3_C Constrained U(ij) Components(s) for O85#       .          6 Check
PLAT218_ALERT_3_C Constrained U(ij) Components(s) for C286#      .          6 Check
PLAT218_ALERT_3_C Constrained U(ij) Components(s) for O86#       .          6 Check
PLAT218_ALERT_3_C Constrained U(ij) Components(s) for N73#       .          6 Check
PLAT218_ALERT_3_C Constrained U(ij) Components(s) for C287#      .          6 Check
PLAT218_ALERT_3_C Constrained U(ij) Components(s) for C288#      .          6 Check
PLAT218_ALERT_3_C Constrained U(ij) Components(s) for C289#      .          6 Check
PLAT218_ALERT_3_C Constrained U(ij) Components(s) for O87#       .          6 Check
PLAT218_ALERT_3_C Constrained U(ij) Components(s) for N74#       .          6 Check
PLAT218_ALERT_3_C Constrained U(ij) Components(s) for C290#      .          6 Check
PLAT218_ALERT_3_C Constrained U(ij) Components(s) for C291#      .          6 Check
PLAT218_ALERT_3_C Constrained U(ij) Components(s) for C292#      .          6 Check
PLAT218_ALERT_3_C Constrained U(ij) Components(s) for N75#       .          6 Check
```

```
PLAT218_ALERT_3_C Constrained U(ij) Components(s) for O88#       .          6 Check
PLAT218_ALERT_3_C Constrained U(ij) Components(s) for C293#      .          6 Check
PLAT218_ALERT_3_C Constrained U(ij) Components(s) for O89#       .          6 Check
PLAT218_ALERT_3_C Constrained U(ij) Components(s) for O90#       .          6 Check
PLAT218_ALERT_3_C Constrained U(ij) Components(s) for O91#       .          6 Check
PLAT218_ALERT_3_C Constrained U(ij) Components(s) for C294#      .          6 Check
PLAT218_ALERT_3_C Constrained U(ij) Components(s) for C295#      .          6 Check
PLAT218_ALERT_3_C Constrained U(ij) Components(s) for C296#      .          6 Check
PLAT218_ALERT_3_C Constrained U(ij) Components(s) for O92#       .          6 Check
PLAT218_ALERT_3_C Constrained U(ij) Components(s) for C297#      .          6 Check
PLAT218_ALERT_3_C Constrained U(ij) Components(s) for C298#      .          6 Check
PLAT218_ALERT_3_C Constrained U(ij) Components(s) for C299#      .          6 Check
PLAT218_ALERT_3_C Constrained U(ij) Components(s) for O93#       .          6 Check
PLAT218_ALERT_3_C Constrained U(ij) Components(s) for O94#       .          6 Check
PLAT218_ALERT_3_C Constrained U(ij) Components(s) for O95#       .          6 Check
PLAT218_ALERT_3_C Constrained U(ij) Components(s) for O96#       .          6 Check
PLAT218_ALERT_3_C Constrained U(ij) Components(s) for O97#       .          6 Check
PLAT218_ALERT_3_C Constrained U(ij) Components(s) for O98#       .          6 Check
PLAT218_ALERT_3_C Constrained U(ij) Components(s) for O99#       .          6 Check
PLAT218_ALERT_3_C Constrained U(ij) Components(s) for O100#      .          6 Check
PLAT218_ALERT_3_C Constrained U(ij) Components(s) for O101#      .          6 Check
PLAT218_ALERT_3_C Constrained U(ij) Components(s) for O102#      .          6 Check
PLAT218_ALERT_3_C Constrained U(ij) Components(s) for O103#      .          6 Check
PLAT218_ALERT_3_C Constrained U(ij) Components(s) for O104#      .          6 Check
PLAT218_ALERT_3_C Constrained U(ij) Components(s) for O105#      .          6 Check
PLAT218_ALERT_3_C Constrained U(ij) Components(s) for O106#      .          6 Check
PLAT218_ALERT_3_C Constrained U(ij) Components(s) for O107#      .          6 Check
PLAT218_ALERT_3_C Constrained U(ij) Components(s) for O108#      .          6 Check
PLAT218_ALERT_3_C Constrained U(ij) Components(s) for O109#      .          6 Check
PLAT218_ALERT_3_C Constrained U(ij) Components(s) for O110#      .          6 Check
PLAT218_ALERT_3_C Constrained U(ij) Components(s) for O111#      .          6 Check
PLAT218_ALERT_3_C Constrained U(ij) Components(s) for O112#      .          6 Check
PLAT218_ALERT_3_C Constrained U(ij) Components(s) for O113#      .          6 Check
PLAT218_ALERT_3_C Constrained U(ij) Components(s) for O114#      .          6 Check
PLAT218_ALERT_3_C Constrained U(ij) Components(s) for O115#      .          6 Check
PLAT218_ALERT_3_C Constrained U(ij) Components(s) for O116#      .          6 Check
PLAT218_ALERT_3_C Constrained U(ij) Components(s) for O117#      .          6 Check
PLAT218_ALERT_3_C Constrained U(ij) Components(s) for O118#      .          6 Check
PLAT218_ALERT_3_C Constrained U(ij) Components(s) for O119#      .          6 Check
PLAT218_ALERT_3_C Constrained U(ij) Components(s) for O120#      .          6 Check
PLAT218_ALERT_3_C Constrained U(ij) Components(s) for O121#      .          6 Check
PLAT218_ALERT_3_C Constrained U(ij) Components(s) for O122#      .          6 Check
PLAT218_ALERT_3_C Constrained U(ij) Components(s) for O123#      .          6 Check
PLAT218_ALERT_3_C Constrained U(ij) Components(s) for O124#      .          6 Check
PLAT218_ALERT_3_C Constrained U(ij) Components(s) for O125#      .          6 Check
PLAT218_ALERT_3_C Constrained U(ij) Components(s) for O126#      .          6 Check
PLAT218_ALERT_3_C Constrained U(ij) Components(s) for O127#      .          6 Check
PLAT218_ALERT_3_C Constrained U(ij) Components(s) for O128#      .          6 Check
PLAT218_ALERT_3_C Constrained U(ij) Components(s) for O129#      .          6 Check
PLAT218_ALERT_3_C Constrained U(ij) Components(s) for O130#      .          6 Check
PLAT218_ALERT_3_C Constrained U(ij) Components(s) for O131#      .          6 Check
PLAT218_ALERT_3_C Constrained U(ij) Components(s) for O132#      .          6 Check
PLAT218_ALERT_3_C Constrained U(ij) Components(s) for O133#      .          6 Check
PLAT218_ALERT_3_C Constrained U(ij) Components(s) for O134#      .          6 Check
PLAT218_ALERT_3_C Constrained U(ij) Components(s) for O135#      .          6 Check
PLAT218_ALERT_3_C Constrained U(ij) Components(s) for O136#      .          6 Check
PLAT218_ALERT_3_C Constrained U(ij) Components(s) for O137#      .          6 Check
```

```
PLAT218_ALERT_3_C Constrained U(ij) Components(s) for O138#      .          6 Check
PLAT218_ALERT_3_C Constrained U(ij) Components(s) for O139#      .          6 Check
PLAT218_ALERT_3_C Constrained U(ij) Components(s) for O140#      .          6 Check
PLAT218_ALERT_3_C Constrained U(ij) Components(s) for O141#      .          6 Check
PLAT218_ALERT_3_C Constrained U(ij) Components(s) for O142#      .          6 Check
PLAT218_ALERT_3_C Constrained U(ij) Components(s) for O143#      .          6 Check
PLAT218_ALERT_3_C Constrained U(ij) Components(s) for O144#      .          6 Check
PLAT218_ALERT_3_C Constrained U(ij) Components(s) for O145#      .          6 Check
PLAT218_ALERT_3_C Constrained U(ij) Components(s) for O146#      .          6 Check
PLAT218_ALERT_3_C Constrained U(ij) Components(s) for O147#      .          6 Check
PLAT218_ALERT_3_C Constrained U(ij) Components(s) for O148#      .          6 Check
PLAT218_ALERT_3_C Constrained U(ij) Components(s) for O149#      .          6 Check
PLAT218_ALERT_3_C Constrained U(ij) Components(s) for O150#      .          6 Check
PLAT218_ALERT_3_C Constrained U(ij) Components(s) for O151#      .          6 Check
PLAT218_ALERT_3_C Constrained U(ij) Components(s) for O152#      .          6 Check
PLAT218_ALERT_3_C Constrained U(ij) Components(s) for O153#      .          6 Check
PLAT218_ALERT_3_C Constrained U(ij) Components(s) for O154#      .          6 Check
PLAT218_ALERT_3_C Constrained U(ij) Components(s) for O155#      .          6 Check
PLAT218_ALERT_3_C Constrained U(ij) Components(s) for O156#      .          6 Check
PLAT218_ALERT_3_C Constrained U(ij) Components(s) for O157#      .          6 Check
PLAT218_ALERT_3_C Constrained U(ij) Components(s) for O158#      .          6 Check
PLAT218_ALERT_3_C Constrained U(ij) Components(s) for O159#      .          6 Check
PLAT218_ALERT_3_C Constrained U(ij) Components(s) for O160#      .          6 Check
PLAT218_ALERT_3_C Constrained U(ij) Components(s) for O161#      .          6 Check
PLAT218_ALERT_3_C Constrained U(ij) Components(s) for O162#      .          6 Check
PLAT218_ALERT_3_C Constrained U(ij) Components(s) for O163#      .          6 Check
PLAT218_ALERT_3_C Constrained U(ij) Components(s) for O164#      .          6 Check
PLAT218_ALERT_3_C Constrained U(ij) Components(s) for O165#      .          6 Check
PLAT218_ALERT_3_C Constrained U(ij) Components(s) for O166#      .          6 Check
PLAT222_ALERT_3_C NonSolvent Resd 1  H   Uiso(max)/Uiso(min) Range       10.0 Ratio
PLAT241_ALERT_2_C High   'MainMol' Ueq as Compared to Neighbors of    Cg_A:41 Check
PLAT242_ALERT_2_C Low    'MainMol' Ueq as Compared to Neighbors of    Cg_A:12 Check
PLAT242_ALERT_2_C Low    'MainMol' Ueq as Compared to Neighbors of    Cg_A:14 Check
PLAT242_ALERT_2_C Low    'MainMol' Ueq as Compared to Neighbors of    Cz_A:17 Check
PLAT242_ALERT_2_C Low    'MainMol' Ueq as Compared to Neighbors of    Cg_A:18 Check
PLAT242_ALERT_2_C Low    'MainMol' Ueq as Compared to Neighbors of    Cd_A:23 Check
PLAT242_ALERT_2_C Low    'MainMol' Ueq as Compared to Neighbors of    Cb_A:34 Check
PLAT242_ALERT_2_C Low    'MainMol' Ueq as Compared to Neighbors of    Cb_A:35 Check
PLAT242_ALERT_2_C Low    'MainMol' Ueq as Compared to Neighbors of    Cg1_A:3 Check
PLAT242_ALERT_2_C Low    'MainMol' Ueq as Compared to Neighbors of    Cg_A:46 Check
PLAT245_ALERT_2_C U(iso) H2_S:19   Smaller than U(eq) O_S:19    by      0.016 Ang**2
PLAT245_ALERT_2_C U(iso) H1_S:19   Smaller than U(eq) O_S:19    by      0.016 Ang**2
PLAT260_ALERT_2_C Large Average Ueq of Residue Including   Ox_S:29      0.281 Check
PLAT260_ALERT_2_C Large Average Ueq of Residue Including  Ox_A:101      0.196 Check
PLAT260_ALERT_2_C Large Average Ueq of Residue Including     O_S:3      0.123 Check
PLAT260_ALERT_2_C Large Average Ueq of Residue Including     O_S:9      0.148 Check
PLAT260_ALERT_2_C Large Average Ueq of Residue Including    O_S:13      0.111 Check
PLAT260_ALERT_2_C Large Average Ueq of Residue Including    O_S:14      0.120 Check
PLAT260_ALERT_2_C Large Average Ueq of Residue Including   Ox_S:16      0.141 Check
PLAT260_ALERT_2_C Large Average Ueq of Residue Including    O_S:17      0.145 Check
PLAT260_ALERT_2_C Large Average Ueq of Residue Including    O_S:18      0.105 Check
PLAT260_ALERT_2_C Large Average Ueq of Residue Including    O_S:20      0.109 Check
PLAT260_ALERT_2_C Large Average Ueq of Residue Including    O_S:21      0.102 Check
PLAT260_ALERT_2_C Large Average Ueq of Residue Including    O_S:23      0.117 Check
PLAT260_ALERT_2_C Large Average Ueq of Residue Including    O_S:24      0.115 Check
PLAT260_ALERT_2_C Large Average Ueq of Residue Including    O_S:25      0.124 Check
PLAT260_ALERT_2_C Large Average Ueq of Residue Including    O_S:27      0.147 Check
```

```
PLAT260_ALERT_2_C Large Average Ueq of Residue Including    O_S:29      0.117 Check
PLAT260_ALERT_2_C Large Average Ueq of Residue Including    O_S:30      0.130 Check
PLAT260_ALERT_2_C Large Average Ueq of Residue Including   Ox_S:33      0.160 Check
PLAT260_ALERT_2_C Large Average Ueq of Residue Including    O_S:34      0.177 Check
PLAT260_ALERT_2_C Large Average Ueq of Residue Including    O_S:35      0.219 Check
PLAT260_ALERT_2_C Large Average Ueq of Residue Including    O_S:36      0.203 Check
PLAT260_ALERT_2_C Large Average Ueq of Residue Including    O_S:37      0.245 Check
PLAT260_ALERT_2_C Large Average Ueq of Residue Including    O_S:38      0.162 Check
PLAT260_ALERT_2_C Large Average Ueq of Residue Including    O_S:40      0.264 Check
PLAT260_ALERT_2_C Large Average Ueq of Residue Including    O_S:41      0.179 Check
PLAT260_ALERT_2_C Large Average Ueq of Residue Including    O_S:42      0.268 Check
PLAT260_ALERT_2_C Large Average Ueq of Residue Including    O_S:46      0.266 Check
PLAT260_ALERT_2_C Large Average Ueq of Residue Including   Ox_S:11      0.193 Check
PLAT260_ALERT_2_C Large Average Ueq of Residue Including   Ox_S:18      0.148 Check
PLAT260_ALERT_2_C Large Average Ueq of Residue Including    O_S:22      0.110 Check
PLAT260_ALERT_2_C Large Average Ueq of Residue Including   Ox_S:22      0.123 Check
PLAT260_ALERT_2_C Large Average Ueq of Residue Including   Ox_S:26      0.209 Check
PLAT303_ALERT_2_C Full Occupancy Atom Hg1D_A:34 with # Connections       1.22 Check
PLAT309_ALERT_2_C Single Bonded Oxygen (C-O > 1.3 Ang) ..........    Og_A:22 Check
PLAT309_ALERT_2_C Single Bonded Oxygen (C-O > 1.3 Ang) ..........   OgY_A:22 Check
PLAT309_ALERT_2_C Single Bonded Oxygen (C-O > 1.3 Ang) ..........    O_A:101 Check
PLAT309_ALERT_2_C Single Bonded Oxygen (C-O > 1.3 Ang) ..........   Ox_A:101 Check
PLAT417_ALERT_2_C Short Inter D-H..H-D       H1_S:1   ..H2_S:7   .       2.13 Ang.
                                           -1+x,y,z  =      1_455 Check
PLAT741_ALERT_1_C Bond    Calc  1.8097(18), Rep     1.80970 ......    Missing s.u.
              SG_A:3   -CB_A:3         1_555   1_555 ........     #   32 Check
PLAT741_ALERT_1_C Bond    Calc    2.010(2), Rep     2.00980 ......    Missing s.u.
              SG_A:3   -SG_A:40        1_555   1_555 ........     #   33 Check
PLAT741_ALERT_1_C Bond    Calc  1.8200(18), Rep     1.81980 ......    Missing s.u.
              SG_A:4   -CB_A:4         1_555   1_555 ........     #   40 Check
PLAT741_ALERT_1_C Bond    Calc    2.038(2), Rep     2.03800 ......    Missing s.u.
              SG_A:4   -SG_A:32        1_555   1_555 ........     #   41 Check
PLAT741_ALERT_1_C Bond    Calc  1.5124(15), Rep     1.51220 ......    Missing s.u.
              CB_A:5   -CG_A:5         1_555   1_555 ........     #   49 Check
PLAT741_ALERT_1_C Bond    Calc  1.5249(15), Rep     1.52480 ......    Missing s.u.
              CG_A:5   -CD_A:5         1_555   1_555 ........     #   50 Check
PLAT741_ALERT_1_C Bond    Calc  1.4038(14), Rep     1.40370 ......    Missing s.u.
              OG_A:6   -CB_A:6         1_555   1_555 ........     #   57 Check
PLAT741_ALERT_1_C Bond    Calc  1.4170(14), Rep     1.41690 ......    Missing s.u.
              CB_A:11  -OG_A:11        1_555   1_555 ........     #  126 Check
PLAT741_ALERT_1_C Bond    Calc  1.3265(13), Rep     1.32640 ......    Missing s.u.
              ND2_A:12 -CG_A:12        1_555   1_555 ........     #  132 Check
PLAT741_ALERT_1_C Bond    Calc  1.2098(12), Rep     1.20980 ......    Missing s.u.
              OD1_A:12 -CG_A:12        1_555   1_555 ........     #  133 Check
PLAT741_ALERT_1_C Bond    Calc  1.5086(15), Rep     1.50850 ......    Missing s.u.
              CB_A:12  -CG_A:12        1_555   1_555 ........     #  134 Check
PLAT741_ALERT_1_C Bond    Calc  1.5405(16), Rep     1.54050 ......    Missing s.u.
              CB_A:14  -CG_A:14        1_555   1_555 ........     #  165 Check
PLAT741_ALERT_1_C Bond    Calc  1.2164(12), Rep     1.21640 ......    Missing s.u.
              CG_A:14  -OD1_A:14       1_555   1_555 ........     #  166 Check
PLAT741_ALERT_1_C Bond    Calc  1.3179(13), Rep     1.31780 ......    Missing s.u.
              CG_A:14  -ND2_A:14       1_555   1_555 ........     #  167 Check
PLAT741_ALERT_1_C Bond    Calc  1.5222(15), Rep     1.52220 ......    Missing s.u.
              CB_A:15  -CG2_A:15       1_555   1_555 ........     #  173 Check
PLAT741_ALERT_1_C Bond    Calc  1.5239(15), Rep     1.52380 ......    Missing s.u.
              CB_A:15  -CG1_A:15       1_555   1_555 ........     #  174 Check
PLAT741_ALERT_1_C Bond    Calc  1.8185(18), Rep     1.81850 ......    Missing s.u.
```

```
              CB_A:16  -SG_A:16         1_555   1_555 ........     #  179 Check
PLAT741_ALERT_1_C Bond    Calc    2.036(2), Rep     2.03560 ......    Missing s.u.
              SG_A:16  -SG_A:26         1_555   1_555 ........     #  180 Check
PLAT741_ALERT_1_C Bond    Calc  1.5246(15), Rep     1.52450 ......    Missing s.u.
              CB_A:17  -CG_A:17         1_555   1_555 ........     #  187 Check
PLAT741_ALERT_1_C Bond    Calc  1.5158(15), Rep     1.51570 ......    Missing s.u.
              CG_A:17  -CD_A:17         1_555   1_555 ........     #  188 Check
PLAT741_ALERT_1_C Bond    Calc  1.4629(15), Rep     1.46280 ......    Missing s.u.
              CD_A:17  -NE_A:17         1_555   1_555 ........     #  189 Check
PLAT741_ALERT_1_C Bond    Calc  1.3252(13), Rep     1.32510 ......    Missing s.u.
              NE_A:17  -CZ_A:17         1_555   1_555 ........     #  190 Check
PLAT741_ALERT_1_C Bond    Calc  1.3096(13), Rep     1.30950 ......    Missing s.u.
              CZ_A:17  -NH1_A:17        1_555   1_555 ........     #  191 Check
PLAT741_ALERT_1_C Bond    Calc  1.3314(13), Rep     1.33140 ......    Missing s.u.
              CZ_A:17  -NH2_A:17        1_555   1_555 ........     #  192 Check
PLAT741_ALERT_1_C Bond    Calc  1.5154(15), Rep     1.51540 ......    Missing s.u.
              CB_A:18  -CG_A:18         1_555   1_555 ........     #  198 Check
PLAT741_ALERT_1_C Bond    Calc  1.5281(15), Rep     1.52810 ......    Missing s.u.
              CD1_A:18 -CG_A:18         1_555   1_555 ........     #  199 Check
PLAT741_ALERT_1_C Bond    Calc  1.5224(15), Rep     1.52240 ......    Missing s.u.
              CD2_A:18 -CG_A:18         1_555   1_555 ........     #  200 Check
PLAT741_ALERT_1_C Bond    Calc  1.4291(14), Rep     1.42910 ......    Missing s.u.
              CB_A:21  -OG1_A:21        1_555   1_555 ........     #  227 Check
PLAT741_ALERT_1_C Bond    Calc  1.5076(15), Rep     1.50750 ......    Missing s.u.
              CB_A:21  -CG2_A:21        1_555   1_555 ........     #  228 Check
PLAT741_ALERT_1_C Bond    Calc  1.5243(15), Rep     1.52430 ......    Missing s.u.
              CB_A:23  -CG_A:23         1_555   1_555 ........     #  256 Check
PLAT741_ALERT_1_C Bond    Calc  1.2139(12), Rep     1.21390 ......    Missing s.u.
              CD_A:23  -OE2_A:23        1_555   1_555 ........     #  257 Check
PLAT741_ALERT_1_C Bond    Calc  1.2310(12), Rep     1.23100 ......    Missing s.u.
              CD_A:23  -OE1_A:23        1_555   1_555 ........     #  258 Check
PLAT741_ALERT_1_C Bond    Calc  1.5316(15), Rep     1.53160 ......    Missing s.u.
              CD_A:23  -CG_A:23         1_555   1_555 ........     #  259 Check
PLAT741_ALERT_1_C Bond    Calc  1.8258(18), Rep     1.82560 ......    Missing s.u.
              SG_A:26  -CB_A:26         1_555   1_555 ........     #  296 Check
PLAT741_ALERT_1_C Bond    Calc  1.4157(14), Rep     1.41560 ......    Missing s.u.
              CB_A:28  -OG1_A:28        1_555   1_555 ........     #  307 Check
PLAT741_ALERT_1_C Bond    Calc  1.5005(15), Rep     1.50050 ......    Missing s.u.
              CB_A:28  -CG2_A:28        1_555   1_555 ........     #  308 Check
PLAT741_ALERT_1_C Bond    Calc  1.5077(15), Rep     1.50750 ......    Missing s.u.
              CG2_A:30 -CB_A:30         1_555   1_555 ........     #  355 Check
PLAT741_ALERT_1_C Bond    Calc  1.4387(15), Rep     1.43870 ......    Missing s.u.
              OG1_A:30 -CB_A:30         1_555   1_555 ........     #  356 Check
PLAT741_ALERT_1_C Bond    Calc  1.8295(19), Rep     1.82950 ......    Missing s.u.
              CB_A:32  -SG_A:32         1_555   1_555 ........     #  366 Check
PLAT741_ALERT_1_C Bond    Calc  1.5219(15), Rep     1.52180 ......    Missing s.u.
              CB_A:33  -CG2_A:33        1_555   1_555 ........     #  372 Check
PLAT741_ALERT_1_C Bond    Calc  1.5386(16), Rep     1.53860 ......    Missing s.u.
              CB_A:33  -CG1_A:33        1_555   1_555 ........     #  373 Check
PLAT741_ALERT_1_C Bond    Calc  1.5125(15), Rep     1.51240 ......    Missing s.u.
              CG1_A:33 -CD1_A:33        1_555   1_555 ........     #  374 Check
PLAT741_ALERT_1_C Bond    Calc  1.5173(15), Rep     1.51730 ......    Missing s.u.
              CB_A:34  -CG1_A:34        1_555   1_555 ........     #  380 Check
PLAT741_ALERT_1_C Bond    Calc  1.5364(16), Rep     1.53640 ......    Missing s.u.
              CB_A:34  -CG2_A:34        1_555   1_555 ........     #  381 Check
PLAT741_ALERT_1_C Bond    Calc  1.5400(16), Rep     1.54000 ......    Missing s.u.
              CB_A:35  -CG1_A:35        1_555   1_555 ........     #  389 Check
```

```
PLAT741_ALERT_1_C Bond    Calc  1.5410(16), Rep     1.54090 ......    Missing s.u.
             CB_A:35  -CG2_A:35        1_555   1_555 ........    #  390 Check
PLAT741_ALERT_1_C Bond    Calc  1.5271(15), Rep     1.52700 ......    Missing s.u.
             CG1_A:35 -CD1_A:35        1_555   1_555 ........    #  391 Check
PLAT741_ALERT_1_C Bond    Calc  1.8207(18), Rep     1.82050 ......    Missing s.u.
             CB_A:40  -SG_A:40         1_555   1_555 ........    #  433 Check
PLAT741_ALERT_1_C Bond    Calc  1.5048(15), Rep     1.50470 ......    Missing s.u.
             CB_A:44  -CG_A:44         1_555   1_555 ........    #  469 Check
PLAT741_ALERT_1_C Bond    Calc  1.3823(14), Rep     1.38220 ......    Missing s.u.
             CG_A:44  -CD1_A:44        1_555   1_555 ........    #  470 Check
PLAT741_ALERT_1_C Bond    Calc  1.3904(14), Rep     1.39040 ......    Missing s.u.
             CG_A:44  -CD2_A:44        1_555   1_555 ........    #  471 Check
PLAT741_ALERT_1_C Bond    Calc  1.3733(14), Rep     1.37330 ......    Missing s.u.
             CD1_A:44 -CE1_A:44        1_555   1_555 ........    #  472 Check
PLAT741_ALERT_1_C Bond    Calc  1.3877(14), Rep     1.38770 ......    Missing s.u.
             CD2_A:44 -CE2_A:44        1_555   1_555 ........    #  473 Check
PLAT741_ALERT_1_C Bond    Calc  1.4074(14), Rep     1.40730 ......    Missing s.u.
             CE1_A:44 -CZ_A:44         1_555   1_555 ........    #  474 Check
PLAT741_ALERT_1_C Bond    Calc  1.3675(14), Rep     1.36740 ......    Missing s.u.
             CE2_A:44 -CZ_A:44         1_555   1_555 ........    #  475 Check
PLAT741_ALERT_1_C Bond    Calc  1.3696(14), Rep     1.36960 ......    Missing s.u.
             CZ_A:44  -OH_A:44         1_555   1_555 ........    #  476 Check
PLAT741_ALERT_1_C Bond    Calc  1.5065(15), Rep     1.50630 ......    Missing s.u.
             CB_A:46  -CG_A:46         1_555   1_555 ........    #  487 Check
PLAT741_ALERT_1_C Bond    Calc  1.2274(12), Rep     1.22730 ......    Missing s.u.
             CG_A:46  -OD1_A:46        1_555   1_555 ........    #  488 Check
PLAT741_ALERT_1_C Bond    Calc  1.3150(13), Rep     1.31500 ......    Missing s.u.
             CG_A:46  -ND2_A:46        1_555   1_555 ........    #  489 Check
PLAT742_ALERT_1_C Angle   Calc   103.67(7), Rep      103.70 ......    Missing s.u.
             CB_A:3  -SG_A:3  -SG_A:40    1_555  1_555  1_555    #   42 Check
PLAT742_ALERT_1_C Angle   Calc   103.96(4), Rep      104.00 ......    Missing s.u.
             CB_A:4  -SG_A:4  -SG_A:32    1_555  1_555  1_555    #   53 Check
PLAT742_ALERT_1_C Angle   Calc   102.80(7), Rep      102.80 ......    Missing s.u.
             CB_A:5  -CG_A:5  -CD_A:5     1_555  1_555  1_555    #   68 Check
PLAT742_ALERT_1_C Angle   Calc   123.61(2), Rep      123.60 ......    Missing s.u.
             OD1_A:12-CG_A:12 -ND2_A:12   1_555  1_555  1_555    #  189 Check
PLAT742_ALERT_1_C Angle   Calc   121.76(5), Rep      121.80 ......    Missing s.u.
             OD1_A:12-CG_A:12 -CB_A:12    1_555  1_555  1_555    #  190 Check
PLAT742_ALERT_1_C Angle   Calc   114.61(6), Rep      114.60 ......    Missing s.u.
             ND2_A:12-CG_A:12 -CB_A:12    1_555  1_555  1_555    #  191 Check
PLAT742_ALERT_1_C Angle   Calc   124.27(5), Rep      124.30 ......    Missing s.u.
             OD1_A:14-CG_A:14 -ND2_A:14   1_555  1_555  1_555    #  230 Check
PLAT742_ALERT_1_C Angle   Calc   120.55(3), Rep      120.60 ......    Missing s.u.
             OD1_A:14-CG_A:14 -CB_A:14    1_555  1_555  1_555    #  231 Check
PLAT742_ALERT_1_C Angle   Calc   115.09(5), Rep      115.10 ......    Missing s.u.
             ND2_A:14-CG_A:14 -CB_A:14    1_555  1_555  1_555    #  232 Check
PLAT742_ALERT_1_C Angle   Calc   110.57(3), Rep      110.60 ......    Missing s.u.
             CG2_A:15-CB_A:15 -CG1_A:15   1_555  1_555  1_555    #  240 Check
PLAT742_ALERT_1_C Angle   Calc   104.14(5), Rep      104.10 ......    Missing s.u.
             CB_A:16 -SG_A:16 -SG_A:26    1_555  1_555  1_555    #  248 Check
PLAT742_ALERT_1_C Angle   Calc   113.38(7), Rep      113.40 ......    Missing s.u.
             CD_A:17 -CG_A:17 -CB_A:17    1_555  1_555  1_555    #  260 Check
PLAT742_ALERT_1_C Angle   Calc   110.08(3), Rep      110.10 ......    Missing s.u.
             NE_A:17 -CD_A:17 -CG_A:17    1_555  1_555  1_555    #  261 Check
PLAT742_ALERT_1_C Angle   Calc   125.78(4), Rep      125.80 ......    Missing s.u.
             CZ_A:17 -NE_A:17 -CD_A:17    1_555  1_555  1_555    #  262 Check
PLAT742_ALERT_1_C Angle   Calc   121.72(5), Rep      121.70 ......    Missing s.u.
```

```
             NH1_A:17-CZ_A:17 -NE_A:17    1_555  1_555  1_555     #  263 Check
PLAT742_ALERT_1_C Angle   Calc   118.97(5), Rep      119.00 ......    Missing s.u.
             NH1_A:17-CZ_A:17 -NH2_A:17   1_555  1_555  1_555     #  264 Check
PLAT742_ALERT_1_C Angle   Calc   119.29(4), Rep      119.30 ......    Missing s.u.
             NE_A:17 -CZ_A:17 -NH2_A:17   1_555  1_555  1_555     #  265 Check
PLAT742_ALERT_1_C Angle   Calc   111.82(6), Rep      111.80 ......    Missing s.u.
             CB_A:18 -CG_A:18 -CD2_A:18   1_555  1_555  1_555     #  279 Check
PLAT742_ALERT_1_C Angle   Calc   108.18(6), Rep      108.20 ......    Missing s.u.
             CB_A:18 -CG_A:18 -CD1_A:18   1_555  1_555  1_555     #  280 Check
PLAT742_ALERT_1_C Angle   Calc   109.85(7), Rep      109.90 ......    Missing s.u.
             CD2_A:18-CG_A:18 -CD1_A:18   1_555  1_555  1_555     #  281 Check
PLAT742_ALERT_1_C Angle   Calc   111.64(7), Rep      111.60 ......    Missing s.u.
             OG1_A:21-CB_A:21 -CG2_A:21   1_555  1_555  1_555     #  316 Check
PLAT742_ALERT_1_C Angle   Calc   122.50(7), Rep      122.50 ......    Missing s.u.
             OE2_A:23-CD_A:23 -OE1_A:23   1_555  1_555  1_555     #  361 Check
PLAT742_ALERT_1_C Angle   Calc   119.97(3), Rep      120.00 ......    Missing s.u.
             OE2_A:23-CD_A:23 -CG_A:23    1_555  1_555  1_555     #  362 Check
PLAT742_ALERT_1_C Angle   Calc   117.35(4), Rep      117.40 ......    Missing s.u.
             OE1_A:23-CD_A:23 -CG_A:23    1_555  1_555  1_555     #  363 Check
PLAT742_ALERT_1_C Angle   Calc   113.11(4), Rep      113.10 ......    Missing s.u.
             CB_A:23 -CG_A:23 -CD_A:23    1_555  1_555  1_555     #  364 Check
PLAT742_ALERT_1_C Angle   Calc   103.15(6), Rep      103.20 ......    Missing s.u.
             CB_A:26 -SG_A:26 -SG_A:16    1_555  1_555  1_555     #  418 Check
PLAT742_ALERT_1_C Angle   Calc   111.37(3), Rep      111.40 ......    Missing s.u.
             OG1_A:28-CB_A:28 -CG2_A:28   1_555  1_555  1_555     #  434 Check
PLAT742_ALERT_1_C Angle   Calc   112.76(5), Rep      112.80 ......    Missing s.u.
             OG1_A:30-CB_A:30 -CG2_A:30   1_555  1_555  1_555     #  504 Check
PLAT742_ALERT_1_C Angle   Calc   105.40(5), Rep      105.40 ......    Missing s.u.
             CB_A:32 -SG_A:32 -SG_A:4     1_555  1_555  1_555     #  520 Check
PLAT742_ALERT_1_C Angle   Calc   112.87(6), Rep      112.90 ......    Missing s.u.
             CG2_A:33-CB_A:33 -CG1_A:33   1_555  1_555  1_555     #  528 Check
PLAT742_ALERT_1_C Angle   Calc   114.65(6), Rep      114.60 ......    Missing s.u.
             CD1_A:33-CG1_A:33-CB_A:33    1_555  1_555  1_555     #  531 Check
PLAT742_ALERT_1_C Angle   Calc   111.62(5), Rep      111.60 ......    Missing s.u.
             CG1_A:34-CB_A:34 -CG2_A:34   1_555  1_555  1_555     #  540 Check
PLAT742_ALERT_1_C Angle   Calc   113.64(3), Rep      113.60 ......    Missing s.u.
             CG1_A:35-CB_A:35 -CG2_A:35   1_555  1_555  1_555     #  551 Check
PLAT742_ALERT_1_C Angle   Calc   111.98(3), Rep      112.00 ......    Missing s.u.
             CD1_A:35-CG1_A:35-CB_A:35    1_555  1_555  1_555     #  554 Check
PLAT742_ALERT_1_C Angle   Calc   102.93(4), Rep      102.90 ......    Missing s.u.
             CB_A:40 -SG_A:40 -SG_A:3     1_555  1_555  1_555     #  618 Check
PLAT742_ALERT_1_C Angle   Calc   118.34(3), Rep      118.30 ......    Missing s.u.
             CD1_A:44-CG_A:44 -CD2_A:44   1_555  1_555  1_555     #  669 Check
PLAT742_ALERT_1_C Angle   Calc   123.24(5), Rep      123.20 ......    Missing s.u.
             CD1_A:44-CG_A:44 -CB_A:44    1_555  1_555  1_555     #  670 Check
PLAT742_ALERT_1_C Angle   Calc   118.42(5), Rep      118.40 ......    Missing s.u.
             CD2_A:44-CG_A:44 -CB_A:44    1_555  1_555  1_555     #  671 Check
PLAT742_ALERT_1_C Angle   Calc   121.33(5), Rep      121.30 ......    Missing s.u.
             CE1_A:44-CD1_A:44-CG_A:44    1_555  1_555  1_555     #  672 Check
PLAT742_ALERT_1_C Angle   Calc   121.42(5), Rep      121.40 ......    Missing s.u.
             CE2_A:44-CD2_A:44-CG_A:44    1_555  1_555  1_555     #  673 Check
PLAT742_ALERT_1_C Angle   Calc   119.29(5), Rep      119.30 ......    Missing s.u.
             CD1_A:44-CE1_A:44-CZ_A:44    1_555  1_555  1_555     #  674 Check
PLAT742_ALERT_1_C Angle   Calc   119.26(5), Rep      119.30 ......    Missing s.u.
             CZ_A:44 -CE2_A:44-CD2_A:44   1_555  1_555  1_555     #  675 Check
PLAT742_ALERT_1_C Angle   Calc   121.85(5), Rep      121.80 ......    Missing s.u.
             CE2_A:44-CZ_A:44 -OH_A:44    1_555  1_555  1_555     #  676 Check
```

```
PLAT742_ALERT_1_C Angle   Calc   120.35(3), Rep      120.40 ......    Missing s.u.
             CE2_A:44-CZ_A:44 -CE1_A:44   1_555  1_555  1_555     #  677 Check
PLAT742_ALERT_1_C Angle   Calc   117.80(5), Rep      117.80 ......    Missing s.u.
             OH_A:44 -CZ_A:44 -CE1_A:44   1_555  1_555  1_555     #  678 Check
PLAT742_ALERT_1_C Angle   Calc   124.10(4), Rep      124.10 ......    Missing s.u.
             OD1_A:46-CG_A:46 -ND2_A:46   1_555  1_555  1_555     #  694 Check
PLAT742_ALERT_1_C Angle   Calc   119.81(5), Rep      119.80 ......    Missing s.u.
             OD1_A:46-CG_A:46 -CB_A:46    1_555  1_555  1_555     #  695 Check
PLAT742_ALERT_1_C Angle   Calc   116.01(3), Rep      116.00 ......    Missing s.u.
             ND2_A:46-CG_A:46 -CB_A:46    1_555  1_555  1_555     #  696 Check
PLAT767_ALERT_4_C INS Embedded LIST 6 Instruction Should be LIST 4     Please Check
PLAT790_ALERT_4_C Centre of Gravity not Within Unit Cell: Resd.  #          1 Note
             C200.33 H311.74 N54.92 O64.56 S6
PLAT910_ALERT_3_C Missing # of FCF Reflection(s) Below Theta(Min).          8 Note
               1  0  0,  -1  0  1,   0  0  1,   0  1  1,   1  0  1,  -1  0  2,
               0  0  2,   1  0  2,
PLAT911_ALERT_3_C Missing FCF Refl Between Thmin & STh/L=    0.600        718 Report
               1 11  0,   2  0  0,  20  6  0,  21  6  0,  22  6  0,  23  6  0,
              24  6  0,  25  5  0,  26  5  0, -26  5  1, -24  6  1, -23  6  1,
             -22  6  1, -21  6  1, -20  6  1,  -2  0  1,  -1 11  1,   0 11  1,
               1  1  1,   1 11  1,  21  6  1,  22  6  1,  23  6  1,  24  5  1,
              25  5  1,  26  5  1, -25  6  2, -24  6  2, -23  6  2, -22  6  2,
             -21  6  2,  -1 11  2,   0  1  2,   0 11  2,   1 11  2,  22  6  2,
              24  5  2,  25  5  2,  26  5  2, -25  6  3, -24  6  3, -23  6  3,
             -22  6  3, -21  6  3, -19  7  3,  -1 11  3,   0 11  3,   1 11  3,
              24  5  3,  25  5  3,  26  5  3, -26  6  4, -25  6  4, -24  6  4,
             -23  6  4, -22  6  4, -21  6  4, -20  7  4, -19  7  4,   0 11  4,
               1 11  4, -26  6  5, -25  6  5, -24  6  5, -23  6  5, -22  6  5,
             -21  6  5, -20  7  5, -19  7  5,   0 11  5, -26  6  6, -25  6  6,
             -24  6  6, -23  6  6, -22  6  6, -21  6  6, -20  7  6, -19  7  6,
             -26  6  7, -25  6  7, -24  6  7, -23  6  7, -22  6  7, -21  6  7,
             -19  7  7,   0 10  7,   1 10  7,   1 20  7, -25  6  8, -24  6  8,
             -23  6  8, -22  6  8, -21  6  8, -19  7  8,   0 10  8,   0 20  8,
PLAT913_ALERT_3_C Missing # of Very Strong Reflections in FCF ....          9 Note
               1  0  0,   2  0  0,  -1  0  1,   0  0  1,   0  1  1,   1  0  1,
               1  1  1,   0  0  2,   0  1  2,
PLAT914_ALERT_3_C No Bijvoet Pairs in FCF for Non-centro Structure     Please Check
PLAT918_ALERT_3_C Reflection(s) with I(obs) much Smaller I(calc) .         24 Check
PLAT975_ALERT_2_C Check Calcd Resid. Dens.  0.70Ang From C2_A:10 .       0.45 eA-3
```

Alert level G

```
ABSMU01_ALERT_1_G  Calculation of _exptl_absorpt_correction_mu
               not performed for this radiation type.
PLAT002_ALERT_2_G Number of Distance or Angle Restraints on AtSite        279 Note
PLAT003_ALERT_2_G Number of Uiso or Uij Restrained non-H Atoms ...        519 Report
PLAT007_ALERT_5_G Number of Unrefined Donor-H Atoms .............         122 Report
             H1#   H2#   H3#   H9#   H19#  H20#  H33#  H37#  H48#  H50#  H54#
             H101# H106# H112# H115# H116# H117# H118# H132# H136# H137# H139#
PLAT033_ALERT_4_G Flack x Value Deviates > 3.0 * sigma from Zero .      0.420 Note
PLAT092_ALERT_4_G Check: Wavelength Given is not Cu,Ga,Mo,Ag,In Ka    0.39950 Ang.
PLAT152_ALERT_1_G The Supplied and Calc. Volume s.u. Differ by ...         -2 Units
PLAT169_ALERT_4_G The CIF-Embedded .res File Contains AFIX 1 Recds          1 Report
PLAT172_ALERT_4_G The CIF-Embedded .res File Contains DFIX Records         72 Report
PLAT173_ALERT_4_G The CIF-Embedded .res File Contains DANG Records         78 Report
PLAT174_ALERT_4_G The CIF-Embedded .res File Contains FLAT Records          4 Report
PLAT178_ALERT_4_G The CIF-Embedded .res File Contains SIMU Records          1 Report
```

PLAT179_ALERT_4_G The CIF-Embedded .res File Contains CHIV Records 23 Report
PLAT188_ALERT_3_G A Non-default SIMU Restraint Value has been used 0.2000 Report
PLAT199_ALERT_1_G Reported _cell_measurement_temperature ..... (K) 293 Check
PLAT200_ALERT_1_G Reported _diffrn_ambient_temperature ..... (K) 293 Check
PLAT300_ALERT_4_G Atom Site Occupancy of Og1_A:1 Constrained at 0.7142 Check
PLAT300_ALERT_4_G Atom Site Occupancy of O_A:1 Constrained at 0.7142 Check
PLAT300_ALERT_4_G Atom Site Occupancy of Ox_A:2 Constrained at 0.6624 Check
PLAT300_ALERT_4_G Atom Site Occupancy of Og1X_A:2 Constrained at 0.6624 Check
PLAT300_ALERT_4_G Atom Site Occupancy of O_A:7 Constrained at 0.6327 Check
PLAT300_ALERT_4_G Atom Site Occupancy of O_A:10 Constrained at 0.5231 Check
PLAT300_ALERT_4_G Atom Site Occupancy of Ox_A:13 Constrained at 0.5121 Check
PLAT300_ALERT_4_G Atom Site Occupancy of O_A:19 Constrained at 0.6696 Check
PLAT300_ALERT_4_G Atom Site Occupancy of O_A:36 Constrained at 0.9225 Check
PLAT300_ALERT_4_G Atom Site Occupancy of Ox_A:37 Constrained at 0.5163 Check
PLAT300_ALERT_4_G Atom Site Occupancy of Og1_A:39 Constrained at 0.637 Check
PLAT300_ALERT_4_G Atom Site Occupancy of O_A:39 Constrained at 0.637 Check
PLAT300_ALERT_4_G Atom Site Occupancy of Od1_A:43 Constrained at 0.5166 Check
PLAT300_ALERT_4_G Atom Site Occupancy of Od2_A:43 Constrained at 0.5166 Check
PLAT300_ALERT_4_G Atom Site Occupancy of O_A:43 Constrained at 0.5166 Check
PLAT300_ALERT_4_G Atom Site Occupancy of N_A:1 Constrained at 0.7142 Check
PLAT300_ALERT_4_G Atom Site Occupancy of Nx_A:2 Constrained at 0.6624 Check
PLAT300_ALERT_4_G Atom Site Occupancy of N_A:7 Constrained at 0.6327 Check
PLAT300_ALERT_4_G Atom Site Occupancy of N_A:10 Constrained at 0.5231 Check
PLAT300_ALERT_4_G Atom Site Occupancy of Ne_A:10 Constrained at 0.5231 Check
PLAT300_ALERT_4_G Atom Site Occupancy of Nh1_A:10 Constrained at 0.5231 Check
PLAT300_ALERT_4_G Atom Site Occupancy of Nh2_A:10 Constrained at 0.5231 Check
PLAT300_ALERT_4_G Atom Site Occupancy of Nx_A:13 Constrained at 0.5121 Check
PLAT300_ALERT_4_G Atom Site Occupancy of N_A:19 Constrained at 0.6696 Check
PLAT300_ALERT_4_G Atom Site Occupancy of N_A:36 Constrained at 0.9225 Check
PLAT300_ALERT_4_G Atom Site Occupancy of Nx_A:37 Constrained at 0.5163 Check
PLAT300_ALERT_4_G Atom Site Occupancy of N_A:39 Constrained at 0.637 Check
PLAT300_ALERT_4_G Atom Site Occupancy of N_A:43 Constrained at 0.5166 Check
PLAT300_ALERT_4_G Atom Site Occupancy of Ox_A:1 Constrained at 0.2858 Check
PLAT300_ALERT_4_G Atom Site Occupancy of Og1X_A:1 Constrained at 0.2858 Check
PLAT300_ALERT_4_G Atom Site Occupancy of O_A:2 Constrained at 0.3376 Check
PLAT300_ALERT_4_G Atom Site Occupancy of Og1_A:2 Constrained at 0.3376 Check
PLAT300_ALERT_4_G Atom Site Occupancy of Ox_A:7 Constrained at 0.3673 Check
PLAT300_ALERT_4_G Atom Site Occupancy of O_A:8 Constrained at 0.4517 Check
PLAT300_ALERT_4_G Atom Site Occupancy of Ox_A:8 Constrained at 0.3975 Check
PLAT300_ALERT_4_G Atom Site Occupancy of Oy_A:8 Constrained at 0.1508 Check
PLAT300_ALERT_4_G Atom Site Occupancy of Ox_A:10 Constrained at 0.4769 Check
PLAT300_ALERT_4_G Atom Site Occupancy of O_A:13 Constrained at 0.4879 Check
PLAT300_ALERT_4_G Atom Site Occupancy of Ox_A:19 Constrained at 0.3304 Check
PLAT300_ALERT_4_G Atom Site Occupancy of Og_A:22 Constrained at 0.3101 Check
PLAT300_ALERT_4_G Atom Site Occupancy of O_A:22 Constrained at 0.3101 Check
PLAT300_ALERT_4_G Atom Site Occupancy of Ox_A:22 Constrained at 0.3589 Check
PLAT300_ALERT_4_G Atom Site Occupancy of OgY_A:22 Constrained at 0.331 Check
PLAT300_ALERT_4_G Atom Site Occupancy of Oy_A:22 Constrained at 0.331 Check
PLAT300_ALERT_4_G Atom Site Occupancy of O_A:25 Constrained at 0.3542 Check
PLAT300_ALERT_4_G Atom Site Occupancy of Ox_A:25 Constrained at 0.3589 Check
PLAT300_ALERT_4_G Atom Site Occupancy of Oy_A:25 Constrained at 0.2869 Check
PLAT300_ALERT_4_G Atom Site Occupancy of Oh_A:29 Constrained at 0.3589 Check
PLAT300_ALERT_4_G Atom Site Occupancy of O_A:29 Constrained at 0.3589 Check
PLAT300_ALERT_4_G Atom Site Occupancy of OhX_A:29 Constrained at 0.2274 Check
PLAT300_ALERT_4_G Atom Site Occupancy of Ox_A:29 Constrained at 0.2274 Check
PLAT300_ALERT_4_G Atom Site Occupancy of OhY_A:29 Constrained at 0.4137 Check
PLAT300_ALERT_4_G Atom Site Occupancy of Oy_A:29 Constrained at 0.4137 Check

```
PLAT300_ALERT_4_G Atom Site Occupancy of O_A:37     Constrained at     0.4837 Check
PLAT300_ALERT_4_G Atom Site Occupancy of Og1X_A:39  Constrained at      0.363 Check
PLAT300_ALERT_4_G Atom Site Occupancy of Ox_A:39    Constrained at      0.363 Check
PLAT300_ALERT_4_G Atom Site Occupancy of Od1X_A:43  Constrained at     0.4834 Check
PLAT300_ALERT_4_G Atom Site Occupancy of Od2X_A:43  Constrained at     0.4834 Check
PLAT300_ALERT_4_G Atom Site Occupancy of Ox_A:43    Constrained at     0.4834 Check
PLAT300_ALERT_4_G Atom Site Occupancy of Ca_A:1     Constrained at     0.7142 Check
PLAT300_ALERT_4_G Atom Site Occupancy of Cb_A:1     Constrained at     0.7142 Check
PLAT300_ALERT_4_G Atom Site Occupancy of Cg2_A:1    Constrained at     0.7142 Check
PLAT300_ALERT_4_G Atom Site Occupancy of C_A:1      Constrained at     0.7142 Check
PLAT300_ALERT_4_G Atom Site Occupancy of Cx_A:2     Constrained at     0.6624 Check
PLAT300_ALERT_4_G Atom Site Occupancy of CbX_A:2    Constrained at     0.6624 Check
PLAT300_ALERT_4_G Atom Site Occupancy of CaX_A:2    Constrained at     0.6624 Check
PLAT300_ALERT_4_G Atom Site Occupancy of Cg2X_A:2   Constrained at     0.6624 Check
PLAT300_ALERT_4_G Atom Site Occupancy of C_A:7      Constrained at     0.6327 Check
PLAT300_ALERT_4_G Atom Site Occupancy of Ca_A:7     Constrained at     0.6327 Check
PLAT300_ALERT_4_G Atom Site Occupancy of Cg1_A:7    Constrained at     0.6327 Check
PLAT300_ALERT_4_G Atom Site Occupancy of Cg2_A:7    Constrained at     0.6327 Check
PLAT300_ALERT_4_G Atom Site Occupancy of Cd1_A:7    Constrained at     0.6327 Check
PLAT300_ALERT_4_G Atom Site Occupancy of Cb_A:7     Constrained at     0.6327 Check
PLAT300_ALERT_4_G Atom Site Occupancy of Ca_A:10    Constrained at     0.5231 Check
PLAT300_ALERT_4_G Atom Site Occupancy of Cb_A:10    Constrained at     0.5231 Check
PLAT300_ALERT_4_G Atom Site Occupancy of Cd_A:10    Constrained at     0.5231 Check
PLAT300_ALERT_4_G Atom Site Occupancy of Cg_A:10    Constrained at     0.5231 Check
PLAT300_ALERT_4_G Atom Site Occupancy of Cz_A:10    Constrained at     0.5231 Check
PLAT300_ALERT_4_G Atom Site Occupancy of C_A:10     Constrained at     0.5231 Check
PLAT300_ALERT_4_G Atom Site Occupancy of CaX_A:13   Constrained at     0.5121 Check
PLAT300_ALERT_4_G Atom Site Occupancy of CbX_A:13   Constrained at     0.5121 Check
PLAT300_ALERT_4_G Atom Site Occupancy of CgX_A:13   Constrained at     0.5121 Check
PLAT300_ALERT_4_G Atom Site Occupancy of Cd1X_A:13  Constrained at     0.5121 Check
PLAT300_ALERT_4_G Atom Site Occupancy of Cd2X_A:13  Constrained at     0.5121 Check
PLAT300_ALERT_4_G Atom Site Occupancy of Ce1X_A:13  Constrained at     0.5121 Check
PLAT300_ALERT_4_G Atom Site Occupancy of Ce2X_A:13  Constrained at     0.5121 Check
PLAT300_ALERT_4_G Atom Site Occupancy of CzX_A:13   Constrained at     0.5121 Check
PLAT300_ALERT_4_G Atom Site Occupancy of Cx_A:13    Constrained at     0.5121 Check
PLAT300_ALERT_4_G Atom Site Occupancy of Ca_A:19    Constrained at     0.6696 Check
PLAT300_ALERT_4_G Atom Site Occupancy of Cb_A:19    Constrained at     0.6696 Check
PLAT300_ALERT_4_G Atom Site Occupancy of Cg_A:19    Constrained at     0.6696 Check
PLAT300_ALERT_4_G Atom Site Occupancy of Cd_A:19    Constrained at     0.6696 Check
PLAT300_ALERT_4_G Atom Site Occupancy of C_A:19     Constrained at     0.6696 Check
PLAT300_ALERT_4_G Atom Site Occupancy of Cd1_A:34   Constrained at     0.7782 Check
PLAT300_ALERT_4_G Atom Site Occupancy of Ca_A:36    Constrained at     0.9225 Check
PLAT300_ALERT_4_G Atom Site Occupancy of Cb_A:36    Constrained at     0.9225 Check
PLAT300_ALERT_4_G Atom Site Occupancy of Cg_A:36    Constrained at     0.9225 Check
PLAT300_ALERT_4_G Atom Site Occupancy of Cd_A:36    Constrained at     0.9225 Check
PLAT300_ALERT_4_G Atom Site Occupancy of C_A:36     Constrained at     0.9225 Check
PLAT300_ALERT_4_G Atom Site Occupancy of CaX_A:37   Constrained at     0.5163 Check
PLAT300_ALERT_4_G Atom Site Occupancy of Cx_A:37    Constrained at     0.5163 Check
PLAT300_ALERT_4_G Atom Site Occupancy of Ca_A:39    Constrained at      0.637 Check
PLAT300_ALERT_4_G Atom Site Occupancy of Cb_A:39    Constrained at      0.637 Check
PLAT300_ALERT_4_G Atom Site Occupancy of Cg2_A:39   Constrained at      0.637 Check
PLAT300_ALERT_4_G Atom Site Occupancy of C_A:39     Constrained at      0.637 Check
PLAT300_ALERT_4_G Atom Site Occupancy of Ca_A:43    Constrained at     0.5166 Check
PLAT300_ALERT_4_G Atom Site Occupancy of Cb_A:43    Constrained at     0.5166 Check
PLAT300_ALERT_4_G Atom Site Occupancy of Cg_A:43    Constrained at     0.5166 Check
PLAT300_ALERT_4_G Atom Site Occupancy of C_A:43     Constrained at     0.5166 Check
PLAT300_ALERT_4_G Atom Site Occupancy of Nx_A:1     Constrained at     0.2858 Check
```

```
PLAT300_ALERT_4_G Atom Site Occupancy of N_A:2      Constrained at     0.3376 Check
PLAT300_ALERT_4_G Atom Site Occupancy of Nx_A:7     Constrained at     0.3673 Check
PLAT300_ALERT_4_G Atom Site Occupancy of N_A:8      Constrained at     0.4517 Check
PLAT300_ALERT_4_G Atom Site Occupancy of Nx_A:8     Constrained at     0.3975 Check
PLAT300_ALERT_4_G Atom Site Occupancy of Ny_A:8     Constrained at     0.1508 Check
PLAT300_ALERT_4_G Atom Site Occupancy of Nx_A:10    Constrained at     0.4769 Check
PLAT300_ALERT_4_G Atom Site Occupancy of NeX_A:10   Constrained at     0.4769 Check
PLAT300_ALERT_4_G Atom Site Occupancy of Nh1X_A:10  Constrained at     0.4769 Check
PLAT300_ALERT_4_G Atom Site Occupancy of Nh2X_A:10  Constrained at     0.4769 Check
PLAT300_ALERT_4_G Atom Site Occupancy of N_A:13     Constrained at     0.4879 Check
PLAT300_ALERT_4_G Atom Site Occupancy of Nx_A:19    Constrained at     0.3304 Check
PLAT300_ALERT_4_G Atom Site Occupancy of N_A:22     Constrained at     0.3101 Check
PLAT300_ALERT_4_G Atom Site Occupancy of Nx_A:22    Constrained at     0.3589 Check
PLAT300_ALERT_4_G Atom Site Occupancy of Ny_A:22    Constrained at      0.331 Check
PLAT300_ALERT_4_G Atom Site Occupancy of N_A:25     Constrained at     0.3542 Check
PLAT300_ALERT_4_G Atom Site Occupancy of Nx_A:25    Constrained at     0.3589 Check
PLAT300_ALERT_4_G Atom Site Occupancy of Ny_A:25    Constrained at     0.2869 Check
PLAT300_ALERT_4_G Atom Site Occupancy of N_A:29     Constrained at     0.3589 Check
PLAT300_ALERT_4_G Atom Site Occupancy of Nx_A:29    Constrained at     0.2274 Check
PLAT300_ALERT_4_G Atom Site Occupancy of Ny_A:29    Constrained at     0.4137 Check
PLAT300_ALERT_4_G Atom Site Occupancy of N_A:37     Constrained at     0.4837 Check
PLAT300_ALERT_4_G Atom Site Occupancy of Nx_A:39    Constrained at      0.363 Check
PLAT300_ALERT_4_G Atom Site Occupancy of Nx_A:43    Constrained at     0.4834 Check
PLAT300_ALERT_4_G Atom Site Occupancy of Cg2X_A:1   Constrained at     0.2858 Check
PLAT300_ALERT_4_G Atom Site Occupancy of CaX_A:1    Constrained at     0.2858 Check
PLAT300_ALERT_4_G Atom Site Occupancy of Cx_A:1     Constrained at     0.2858 Check
PLAT300_ALERT_4_G Atom Site Occupancy of CbX_A:1    Constrained at     0.2858 Check
PLAT300_ALERT_4_G Atom Site Occupancy of C_A:2      Constrained at     0.3376 Check
PLAT300_ALERT_4_G Atom Site Occupancy of Cg2_A:2    Constrained at     0.3376 Check
PLAT300_ALERT_4_G Atom Site Occupancy of Ca_A:2     Constrained at     0.3376 Check
PLAT300_ALERT_4_G Atom Site Occupancy of Cb_A:2     Constrained at     0.3376 Check
PLAT300_ALERT_4_G Atom Site Occupancy of CaX_A:7    Constrained at     0.3673 Check
PLAT300_ALERT_4_G Atom Site Occupancy of Cg1X_A:7   Constrained at     0.3673 Check
PLAT300_ALERT_4_G Atom Site Occupancy of Cg2X_A:7   Constrained at     0.3673 Check
PLAT300_ALERT_4_G Atom Site Occupancy of Cx_A:7     Constrained at     0.3673 Check
PLAT300_ALERT_4_G Atom Site Occupancy of CbX_A:7    Constrained at     0.3673 Check
PLAT300_ALERT_4_G Atom Site Occupancy of Cd1X_A:7   Constrained at     0.3673 Check
PLAT300_ALERT_4_G Atom Site Occupancy of Cb_A:8     Constrained at     0.4517 Check
PLAT300_ALERT_4_G Atom Site Occupancy of Ca_A:8     Constrained at     0.4517 Check
PLAT300_ALERT_4_G Atom Site Occupancy of Cg1_A:8    Constrained at     0.4517 Check
PLAT300_ALERT_4_G Atom Site Occupancy of C_A:8      Constrained at     0.4517 Check
PLAT300_ALERT_4_G Atom Site Occupancy of Cg2_A:8    Constrained at     0.4517 Check
PLAT300_ALERT_4_G Atom Site Occupancy of CaX_A:8    Constrained at     0.3975 Check
PLAT300_ALERT_4_G Atom Site Occupancy of CbX_A:8    Constrained at     0.3975 Check
PLAT300_ALERT_4_G Atom Site Occupancy of Cx_A:8     Constrained at     0.3975 Check
PLAT300_ALERT_4_G Atom Site Occupancy of Cg2X_A:8   Constrained at     0.3975 Check
PLAT300_ALERT_4_G Atom Site Occupancy of Cg1X_A:8   Constrained at     0.3975 Check
PLAT300_ALERT_4_G Atom Site Occupancy of CaY_A:8    Constrained at     0.1508 Check
PLAT300_ALERT_4_G Atom Site Occupancy of CbY_A:8    Constrained at     0.1508 Check
PLAT300_ALERT_4_G Atom Site Occupancy of Cg2Y_A:8   Constrained at     0.1508 Check
PLAT300_ALERT_4_G Atom Site Occupancy of Cg1Y_A:8   Constrained at     0.1508 Check
PLAT300_ALERT_4_G Atom Site Occupancy of Cy_A:8     Constrained at     0.1508 Check
PLAT300_ALERT_4_G Atom Site Occupancy of Cx_A:10    Constrained at     0.4769 Check
PLAT300_ALERT_4_G Atom Site Occupancy of CaX_A:10   Constrained at     0.4769 Check
PLAT300_ALERT_4_G Atom Site Occupancy of CbX_A:10   Constrained at     0.4769 Check
PLAT300_ALERT_4_G Atom Site Occupancy of CdX_A:10   Constrained at     0.4769 Check
PLAT300_ALERT_4_G Atom Site Occupancy of CgX_A:10   Constrained at     0.4769 Check
```

```
PLAT300_ALERT_4_G Atom Site Occupancy of CzX_A:10   Constrained at     0.4769 Check
PLAT300_ALERT_4_G Atom Site Occupancy of C_A:13     Constrained at     0.4879 Check
PLAT300_ALERT_4_G Atom Site Occupancy of Ca_A:13    Constrained at     0.4879 Check
PLAT300_ALERT_4_G Atom Site Occupancy of Cb_A:13    Constrained at     0.4879 Check
PLAT300_ALERT_4_G Atom Site Occupancy of Cg_A:13    Constrained at     0.4879 Check
PLAT300_ALERT_4_G Atom Site Occupancy of Cd2_A:13   Constrained at     0.4879 Check
PLAT300_ALERT_4_G Atom Site Occupancy of Cd1_A:13   Constrained at     0.4879 Check
PLAT300_ALERT_4_G Atom Site Occupancy of Ce1_A:13   Constrained at     0.4879 Check
PLAT300_ALERT_4_G Atom Site Occupancy of Ce2_A:13   Constrained at     0.4879 Check
PLAT300_ALERT_4_G Atom Site Occupancy of Cz_A:13    Constrained at     0.4879 Check
PLAT300_ALERT_4_G Atom Site Occupancy of CaX_A:19   Constrained at     0.3304 Check
PLAT300_ALERT_4_G Atom Site Occupancy of CbX_A:19   Constrained at     0.3304 Check
PLAT300_ALERT_4_G Atom Site Occupancy of CgX_A:19   Constrained at     0.3304 Check
PLAT300_ALERT_4_G Atom Site Occupancy of CdX_A:19   Constrained at     0.3304 Check
PLAT300_ALERT_4_G Atom Site Occupancy of Cx_A:19    Constrained at     0.3304 Check
PLAT300_ALERT_4_G Atom Site Occupancy of Ca_A:22    Constrained at     0.3101 Check
PLAT300_ALERT_4_G Atom Site Occupancy of Cb_A:22    Constrained at     0.3101 Check
PLAT300_ALERT_4_G Atom Site Occupancy of C_A:22     Constrained at     0.3101 Check
PLAT300_ALERT_4_G Atom Site Occupancy of CaX_A:22   Constrained at     0.3589 Check
PLAT300_ALERT_4_G Atom Site Occupancy of CbX_A:22   Constrained at     0.3589 Check
PLAT300_ALERT_4_G Atom Site Occupancy of CgX_A:22   Constrained at     0.3589 Check
PLAT300_ALERT_4_G Atom Site Occupancy of CdX_A:22   Constrained at     0.3589 Check
PLAT300_ALERT_4_G Atom Site Occupancy of Cx_A:22    Constrained at     0.3589 Check
PLAT300_ALERT_4_G Atom Site Occupancy of CaY_A:22   Constrained at      0.331 Check
PLAT300_ALERT_4_G Atom Site Occupancy of CbY_A:22   Constrained at      0.331 Check
PLAT300_ALERT_4_G Atom Site Occupancy of Cy_A:22    Constrained at      0.331 Check
PLAT300_ALERT_4_G Atom Site Occupancy of Ca_A:25    Constrained at     0.3542 Check
PLAT300_ALERT_4_G Atom Site Occupancy of Cb_A:25    Constrained at     0.3542 Check
PLAT300_ALERT_4_G Atom Site Occupancy of Cg1_A:25   Constrained at     0.3542 Check
PLAT300_ALERT_4_G Atom Site Occupancy of Cg2_A:25   Constrained at     0.3542 Check
PLAT300_ALERT_4_G Atom Site Occupancy of Cd1_A:25   Constrained at     0.3542 Check
PLAT300_ALERT_4_G Atom Site Occupancy of C_A:25     Constrained at     0.3542 Check
PLAT300_ALERT_4_G Atom Site Occupancy of CaX_A:25   Constrained at     0.3589 Check
PLAT300_ALERT_4_G Atom Site Occupancy of CbX_A:25   Constrained at     0.3589 Check
PLAT300_ALERT_4_G Atom Site Occupancy of Cd1X_A:25  Constrained at     0.3589 Check
PLAT300_ALERT_4_G Atom Site Occupancy of Cd2X_A:25  Constrained at     0.3589 Check
PLAT300_ALERT_4_G Atom Site Occupancy of CgX_A:25   Constrained at     0.3589 Check
PLAT300_ALERT_4_G Atom Site Occupancy of Cx_A:25    Constrained at     0.3589 Check
PLAT300_ALERT_4_G Atom Site Occupancy of CaY_A:25   Constrained at     0.2869 Check
PLAT300_ALERT_4_G Atom Site Occupancy of CbY_A:25   Constrained at     0.2869 Check
PLAT300_ALERT_4_G Atom Site Occupancy of Cg1Y_A:25  Constrained at     0.2869 Check
PLAT300_ALERT_4_G Atom Site Occupancy of Cg2Y_A:25  Constrained at     0.2869 Check
PLAT300_ALERT_4_G Atom Site Occupancy of Cd1Y_A:25  Constrained at     0.2869 Check
PLAT300_ALERT_4_G Atom Site Occupancy of Cy_A:25    Constrained at     0.2869 Check
PLAT300_ALERT_4_G Atom Site Occupancy of Ca_A:29    Constrained at     0.3589 Check
PLAT300_ALERT_4_G Atom Site Occupancy of Cb_A:29    Constrained at     0.3589 Check
PLAT300_ALERT_4_G Atom Site Occupancy of Cg_A:29    Constrained at     0.3589 Check
PLAT300_ALERT_4_G Atom Site Occupancy of Cd1_A:29   Constrained at     0.3589 Check
PLAT300_ALERT_4_G Atom Site Occupancy of Cd2_A:29   Constrained at     0.3589 Check
PLAT300_ALERT_4_G Atom Site Occupancy of Ce1_A:29   Constrained at     0.3589 Check
PLAT300_ALERT_4_G Atom Site Occupancy of Ce2_A:29   Constrained at     0.3589 Check
PLAT300_ALERT_4_G Atom Site Occupancy of Cz_A:29    Constrained at     0.3589 Check
PLAT300_ALERT_4_G Atom Site Occupancy of C_A:29     Constrained at     0.3589 Check
PLAT300_ALERT_4_G Atom Site Occupancy of CaX_A:29   Constrained at     0.2274 Check
PLAT300_ALERT_4_G Atom Site Occupancy of CbX_A:29   Constrained at     0.2274 Check
PLAT300_ALERT_4_G Atom Site Occupancy of CgX_A:29   Constrained at     0.2274 Check
PLAT300_ALERT_4_G Atom Site Occupancy of Cd1X_A:29  Constrained at     0.2274 Check
```

PLAT300_ALERT_4_G Atom Site Occupancy of Cd2X_A:29 Constrained at 0.2274 Check
PLAT300_ALERT_4_G Atom Site Occupancy of Ce1X_A:29 Constrained at 0.2274 Check
PLAT300_ALERT_4_G Atom Site Occupancy of Ce2X_A:29 Constrained at 0.2274 Check
PLAT300_ALERT_4_G Atom Site Occupancy of CzX_A:29 Constrained at 0.2274 Check
PLAT300_ALERT_4_G Atom Site Occupancy of Cx_A:29 Constrained at 0.2274 Check
PLAT300_ALERT_4_G Atom Site Occupancy of CaY_A:29 Constrained at 0.4137 Check
PLAT300_ALERT_4_G Atom Site Occupancy of CbY_A:29 Constrained at 0.4137 Check
PLAT300_ALERT_4_G Atom Site Occupancy of CgY_A:29 Constrained at 0.4137 Check
PLAT300_ALERT_4_G Atom Site Occupancy of Cd1Y_A:29 Constrained at 0.4137 Check
PLAT300_ALERT_4_G Atom Site Occupancy of Cd2Y_A:29 Constrained at 0.4137 Check
PLAT300_ALERT_4_G Atom Site Occupancy of Ce2Y_A:29 Constrained at 0.4137 Check
PLAT300_ALERT_4_G Atom Site Occupancy of Ce1Y_A:29 Constrained at 0.4137 Check
PLAT300_ALERT_4_G Atom Site Occupancy of CzY_A:29 Constrained at 0.4137 Check
PLAT300_ALERT_4_G Atom Site Occupancy of Cy_A:29 Constrained at 0.4137 Check
PLAT300_ALERT_4_G Atom Site Occupancy of Cd1Y_A:34 Constrained at 0.2218 Check
PLAT300_ALERT_4_G Atom Site Occupancy of Ca_A:37 Constrained at 0.4837 Check
PLAT300_ALERT_4_G Atom Site Occupancy of C_A:37 Constrained at 0.4837 Check
PLAT300_ALERT_4_G Atom Site Occupancy of CaX_A:39 Constrained at 0.363 Check
PLAT300_ALERT_4_G Atom Site Occupancy of CbX_A:39 Constrained at 0.363 Check
PLAT300_ALERT_4_G Atom Site Occupancy of Cg2X_A:39 Constrained at 0.363 Check
PLAT300_ALERT_4_G Atom Site Occupancy of Cx_A:39 Constrained at 0.363 Check
PLAT300_ALERT_4_G Atom Site Occupancy of CaX_A:43 Constrained at 0.4834 Check
PLAT300_ALERT_4_G Atom Site Occupancy of CbX_A:43 Constrained at 0.4834 Check
PLAT300_ALERT_4_G Atom Site Occupancy of CgX_A:43 Constrained at 0.4834 Check
PLAT300_ALERT_4_G Atom Site Occupancy of Cx_A:43 Constrained at 0.4834 Check
PLAT300_ALERT_4_G Atom Site Occupancy of H0A_A:1 Constrained at 0.7142 Check
PLAT300_ALERT_4_G Atom Site Occupancy of H0B_A:1 Constrained at 0.7142 Check
PLAT300_ALERT_4_G Atom Site Occupancy of H0C_A:1 Constrained at 0.7142 Check
PLAT300_ALERT_4_G Atom Site Occupancy of Ha_A:1 Constrained at 0.7142 Check
PLAT300_ALERT_4_G Atom Site Occupancy of Hb_A:1 Constrained at 0.7142 Check
PLAT300_ALERT_4_G Atom Site Occupancy of Hg2A_A:1 Constrained at 0.7142 Check
PLAT300_ALERT_4_G Atom Site Occupancy of Hg2B_A:1 Constrained at 0.7142 Check
PLAT300_ALERT_4_G Atom Site Occupancy of Hg2C_A:1 Constrained at 0.7142 Check
PLAT300_ALERT_4_G Atom Site Occupancy of Hg1_A:1 Constrained at 0.7142 Check
PLAT300_ALERT_4_G Atom Site Occupancy of H0_A:2 Constrained at 0.6624 Check
PLAT300_ALERT_4_G Atom Site Occupancy of Hg1_A:2 Constrained at 0.6624 Check
PLAT300_ALERT_4_G Atom Site Occupancy of Hb_A:2 Constrained at 0.6624 Check
PLAT300_ALERT_4_G Atom Site Occupancy of Ha_A:2 Constrained at 0.6624 Check
PLAT300_ALERT_4_G Atom Site Occupancy of Hg2A_A:2 Constrained at 0.6624 Check
PLAT300_ALERT_4_G Atom Site Occupancy of Hg2B_A:2 Constrained at 0.6624 Check
PLAT300_ALERT_4_G Atom Site Occupancy of Hg2C_A:2 Constrained at 0.6624 Check
PLAT300_ALERT_4_G Atom Site Occupancy of Ha_A:7 Constrained at 0.6327 Check
PLAT300_ALERT_4_G Atom Site Occupancy of H0_A:7 Constrained at 0.6327 Check
PLAT300_ALERT_4_G Atom Site Occupancy of Hg1A_A:7 Constrained at 0.6327 Check
PLAT300_ALERT_4_G Atom Site Occupancy of Hg1B_A:7 Constrained at 0.6327 Check
PLAT300_ALERT_4_G Atom Site Occupancy of Hg2A_A:7 Constrained at 0.6327 Check
PLAT300_ALERT_4_G Atom Site Occupancy of Hg2B_A:7 Constrained at 0.6327 Check
PLAT300_ALERT_4_G Atom Site Occupancy of Hg2C_A:7 Constrained at 0.6327 Check
PLAT300_ALERT_4_G Atom Site Occupancy of Hd1A_A:7 Constrained at 0.6327 Check
PLAT300_ALERT_4_G Atom Site Occupancy of Hd1B_A:7 Constrained at 0.6327 Check
PLAT300_ALERT_4_G Atom Site Occupancy of Hd1C_A:7 Constrained at 0.6327 Check
PLAT300_ALERT_4_G Atom Site Occupancy of Hb_A:7 Constrained at 0.6327 Check
PLAT300_ALERT_4_G Atom Site Occupancy of H0_A:10 Constrained at 0.5231 Check
PLAT300_ALERT_4_G Atom Site Occupancy of Ha_A:10 Constrained at 0.5231 Check
PLAT300_ALERT_4_G Atom Site Occupancy of Hb1_A:10 Constrained at 0.5231 Check
PLAT300_ALERT_4_G Atom Site Occupancy of Hb2_A:10 Constrained at 0.5231 Check
PLAT300_ALERT_4_G Atom Site Occupancy of Hd1_A:10 Constrained at 0.5231 Check

```
PLAT300_ALERT_4_G Atom Site Occupancy of Hd2_A:10   Constrained at     0.5231 Check
PLAT300_ALERT_4_G Atom Site Occupancy of He_A:10    Constrained at     0.5231 Check
PLAT300_ALERT_4_G Atom Site Occupancy of Hg1_A:10   Constrained at     0.5231 Check
PLAT300_ALERT_4_G Atom Site Occupancy of Hg2_A:10   Constrained at     0.5231 Check
PLAT300_ALERT_4_G Atom Site Occupancy of Hh1A_A:10  Constrained at     0.5231 Check
PLAT300_ALERT_4_G Atom Site Occupancy of Hh1B_A:10  Constrained at     0.5231 Check
PLAT300_ALERT_4_G Atom Site Occupancy of Hh2A_A:10  Constrained at     0.5231 Check
PLAT300_ALERT_4_G Atom Site Occupancy of Hh2B_A:10  Constrained at     0.5231 Check
PLAT300_ALERT_4_G Atom Site Occupancy of H0A_A:13   Constrained at     0.5121 Check
PLAT300_ALERT_4_G Atom Site Occupancy of Ha1_A:13   Constrained at     0.5121 Check
PLAT300_ALERT_4_G Atom Site Occupancy of Hb3_A:13   Constrained at     0.5121 Check
PLAT300_ALERT_4_G Atom Site Occupancy of Hb4_A:13   Constrained at     0.5121 Check
PLAT300_ALERT_4_G Atom Site Occupancy of Hd1A_A:13  Constrained at     0.5121 Check
PLAT300_ALERT_4_G Atom Site Occupancy of Hd2A_A:13  Constrained at     0.5121 Check
PLAT300_ALERT_4_G Atom Site Occupancy of He1A_A:13  Constrained at     0.5121 Check
PLAT300_ALERT_4_G Atom Site Occupancy of He2A_A:13  Constrained at     0.5121 Check
PLAT300_ALERT_4_G Atom Site Occupancy of Hz1_A:13   Constrained at     0.5121 Check
PLAT300_ALERT_4_G Atom Site Occupancy of Ha_A:19    Constrained at     0.6696 Check
PLAT300_ALERT_4_G Atom Site Occupancy of Hb1_A:19   Constrained at     0.6696 Check
PLAT300_ALERT_4_G Atom Site Occupancy of Hb2_A:19   Constrained at     0.6696 Check
PLAT300_ALERT_4_G Atom Site Occupancy of Hg1_A:19   Constrained at     0.6696 Check
PLAT300_ALERT_4_G Atom Site Occupancy of Hg2_A:19   Constrained at     0.6696 Check
PLAT300_ALERT_4_G Atom Site Occupancy of Hd1_A:19   Constrained at     0.6696 Check
PLAT300_ALERT_4_G Atom Site Occupancy of Hd2_A:19   Constrained at     0.6696 Check
PLAT300_ALERT_4_G Atom Site Occupancy of Hd1X_A:34  Constrained at     0.7782 Check
PLAT300_ALERT_4_G Atom Site Occupancy of Hd1Y_A:34  Constrained at     0.7782 Check
PLAT300_ALERT_4_G Atom Site Occupancy of Hd1Z_A:34  Constrained at     0.7782 Check
PLAT300_ALERT_4_G Atom Site Occupancy of Ha_A:36    Constrained at     0.9225 Check
PLAT300_ALERT_4_G Atom Site Occupancy of Hb1_A:36   Constrained at     0.9225 Check
PLAT300_ALERT_4_G Atom Site Occupancy of Hb2_A:36   Constrained at     0.9225 Check
PLAT300_ALERT_4_G Atom Site Occupancy of Hg1_A:36   Constrained at     0.9225 Check
PLAT300_ALERT_4_G Atom Site Occupancy of Hg2_A:36   Constrained at     0.9225 Check
PLAT300_ALERT_4_G Atom Site Occupancy of Hd1_A:36   Constrained at     0.9225 Check
PLAT300_ALERT_4_G Atom Site Occupancy of Hd2_A:36   Constrained at     0.9225 Check
PLAT300_ALERT_4_G Atom Site Occupancy of H0A_A:37   Constrained at     0.5163 Check
PLAT300_ALERT_4_G Atom Site Occupancy of Ha3_A:37   Constrained at     0.5163 Check
PLAT300_ALERT_4_G Atom Site Occupancy of Ha4_A:37   Constrained at     0.5163 Check
PLAT300_ALERT_4_G Atom Site Occupancy of H0_A:39    Constrained at      0.637 Check
PLAT300_ALERT_4_G Atom Site Occupancy of Ha_A:39    Constrained at      0.637 Check
PLAT300_ALERT_4_G Atom Site Occupancy of Hb_A:39    Constrained at      0.637 Check
PLAT300_ALERT_4_G Atom Site Occupancy of Hg1_A:39   Constrained at      0.637 Check
PLAT300_ALERT_4_G Atom Site Occupancy of Hg2A_A:39  Constrained at      0.637 Check
PLAT300_ALERT_4_G Atom Site Occupancy of Hg2B_A:39  Constrained at      0.637 Check
PLAT300_ALERT_4_G Atom Site Occupancy of Hg2C_A:39  Constrained at      0.637 Check
PLAT300_ALERT_4_G Atom Site Occupancy of H0_A:43    Constrained at     0.5166 Check
PLAT300_ALERT_4_G Atom Site Occupancy of Ha_A:43    Constrained at     0.5166 Check
PLAT300_ALERT_4_G Atom Site Occupancy of Hb1_A:43   Constrained at     0.5166 Check
PLAT300_ALERT_4_G Atom Site Occupancy of Hb2_A:43   Constrained at     0.5166 Check
PLAT300_ALERT_4_G Atom Site Occupancy of Hg2D_A:1   Constrained at     0.2858 Check
PLAT300_ALERT_4_G Atom Site Occupancy of Hg2E_A:1   Constrained at     0.2858 Check
PLAT300_ALERT_4_G Atom Site Occupancy of Hg2F_A:1   Constrained at     0.2858 Check
PLAT300_ALERT_4_G Atom Site Occupancy of Ha1_A:1    Constrained at     0.2858 Check
PLAT300_ALERT_4_G Atom Site Occupancy of H0D_A:1    Constrained at     0.2858 Check
PLAT300_ALERT_4_G Atom Site Occupancy of H0E_A:1    Constrained at     0.2858 Check
PLAT300_ALERT_4_G Atom Site Occupancy of H0F_A:1    Constrained at     0.2858 Check
PLAT300_ALERT_4_G Atom Site Occupancy of Hb1_A:1    Constrained at     0.2858 Check
PLAT300_ALERT_4_G Atom Site Occupancy of Hg1A_A:1   Constrained at     0.2858 Check
```

```
PLAT300_ALERT_4_G Atom Site Occupancy of Hg2D_A:2   Constrained at     0.3376 Check
PLAT300_ALERT_4_G Atom Site Occupancy of Hg2E_A:2   Constrained at     0.3376 Check
PLAT300_ALERT_4_G Atom Site Occupancy of Hg2F_A:2   Constrained at     0.3376 Check
PLAT300_ALERT_4_G Atom Site Occupancy of Hg1A_A:2   Constrained at     0.3376 Check
PLAT300_ALERT_4_G Atom Site Occupancy of Ha1_A:2    Constrained at     0.3376 Check
PLAT300_ALERT_4_G Atom Site Occupancy of Hb1_A:2    Constrained at     0.3376 Check
PLAT300_ALERT_4_G Atom Site Occupancy of H0A_A:2    Constrained at     0.3376 Check
PLAT300_ALERT_4_G Atom Site Occupancy of Ha1_A:7    Constrained at     0.3673 Check
PLAT300_ALERT_4_G Atom Site Occupancy of Hg1C_A:7   Constrained at     0.3673 Check
PLAT300_ALERT_4_G Atom Site Occupancy of Hg1D_A:7   Constrained at     0.3673 Check
PLAT300_ALERT_4_G Atom Site Occupancy of Hg2D_A:7   Constrained at     0.3673 Check
PLAT300_ALERT_4_G Atom Site Occupancy of Hg2E_A:7   Constrained at     0.3673 Check
PLAT300_ALERT_4_G Atom Site Occupancy of Hg2F_A:7   Constrained at     0.3673 Check
PLAT300_ALERT_4_G Atom Site Occupancy of H0A_A:7    Constrained at     0.3673 Check
PLAT300_ALERT_4_G Atom Site Occupancy of Hb1_A:7    Constrained at     0.3673 Check
PLAT300_ALERT_4_G Atom Site Occupancy of Hd1D_A:7   Constrained at     0.3673 Check
PLAT300_ALERT_4_G Atom Site Occupancy of Hd1E_A:7   Constrained at     0.3673 Check
PLAT300_ALERT_4_G Atom Site Occupancy of Hd1F_A:7   Constrained at     0.3673 Check
PLAT300_ALERT_4_G Atom Site Occupancy of HbX_A:8    Constrained at     0.4517 Check
PLAT300_ALERT_4_G Atom Site Occupancy of HaX_A:8    Constrained at     0.4517 Check
PLAT300_ALERT_4_G Atom Site Occupancy of Hg1A_A:8   Constrained at     0.4517 Check
PLAT300_ALERT_4_G Atom Site Occupancy of Hg1B_A:8   Constrained at     0.4517 Check
PLAT300_ALERT_4_G Atom Site Occupancy of Hg1C_A:8   Constrained at     0.4517 Check
PLAT300_ALERT_4_G Atom Site Occupancy of H0_A:8     Constrained at     0.4517 Check
PLAT300_ALERT_4_G Atom Site Occupancy of Hg2A_A:8   Constrained at     0.4517 Check
PLAT300_ALERT_4_G Atom Site Occupancy of Hg2B_A:8   Constrained at     0.4517 Check
PLAT300_ALERT_4_G Atom Site Occupancy of Hg2C_A:8   Constrained at     0.4517 Check
PLAT300_ALERT_4_G Atom Site Occupancy of Ha1_A:8    Constrained at     0.3975 Check
PLAT300_ALERT_4_G Atom Site Occupancy of Hb1_A:8    Constrained at     0.3975 Check
PLAT300_ALERT_4_G Atom Site Occupancy of Hg2D_A:8   Constrained at     0.3975 Check
PLAT300_ALERT_4_G Atom Site Occupancy of Hg2E_A:8   Constrained at     0.3975 Check
PLAT300_ALERT_4_G Atom Site Occupancy of Hg2F_A:8   Constrained at     0.3975 Check
PLAT300_ALERT_4_G Atom Site Occupancy of H0A_A:8    Constrained at     0.3975 Check
PLAT300_ALERT_4_G Atom Site Occupancy of Hg1D_A:8   Constrained at     0.3975 Check
PLAT300_ALERT_4_G Atom Site Occupancy of Hg1E_A:8   Constrained at     0.3975 Check
PLAT300_ALERT_4_G Atom Site Occupancy of Hg1F_A:8   Constrained at     0.3975 Check
PLAT300_ALERT_4_G Atom Site Occupancy of Ha_A:8     Constrained at     0.1508 Check
PLAT300_ALERT_4_G Atom Site Occupancy of Hb_A:8     Constrained at     0.1508 Check
PLAT300_ALERT_4_G Atom Site Occupancy of Hg21_A:8   Constrained at     0.1508 Check
PLAT300_ALERT_4_G Atom Site Occupancy of Hg22_A:8   Constrained at     0.1508 Check
PLAT300_ALERT_4_G Atom Site Occupancy of Hg23_A:8   Constrained at     0.1508 Check
PLAT300_ALERT_4_G Atom Site Occupancy of Hg11_A:8   Constrained at     0.1508 Check
PLAT300_ALERT_4_G Atom Site Occupancy of Hg12_A:8   Constrained at     0.1508 Check
PLAT300_ALERT_4_G Atom Site Occupancy of Hg13_A:8   Constrained at     0.1508 Check
PLAT300_ALERT_4_G Atom Site Occupancy of H0A_A:10   Constrained at     0.4769 Check
PLAT300_ALERT_4_G Atom Site Occupancy of Ha1_A:10   Constrained at     0.4769 Check
PLAT300_ALERT_4_G Atom Site Occupancy of Hb3_A:10   Constrained at     0.4769 Check
PLAT300_ALERT_4_G Atom Site Occupancy of Hb4_A:10   Constrained at     0.4769 Check
PLAT300_ALERT_4_G Atom Site Occupancy of Hd3_A:10   Constrained at     0.4769 Check
PLAT300_ALERT_4_G Atom Site Occupancy of Hd4_A:10   Constrained at     0.4769 Check
PLAT300_ALERT_4_G Atom Site Occupancy of Hg3_A:10   Constrained at     0.4769 Check
PLAT300_ALERT_4_G Atom Site Occupancy of Hg4_A:10   Constrained at     0.4769 Check
PLAT300_ALERT_4_G Atom Site Occupancy of He1_A:10   Constrained at     0.4769 Check
PLAT300_ALERT_4_G Atom Site Occupancy of Hh1C_A:10  Constrained at     0.4769 Check
PLAT300_ALERT_4_G Atom Site Occupancy of Hh1D_A:10  Constrained at     0.4769 Check
PLAT300_ALERT_4_G Atom Site Occupancy of Hh2C_A:10  Constrained at     0.4769 Check
PLAT300_ALERT_4_G Atom Site Occupancy of Hh2D_A:10  Constrained at     0.4769 Check
```

PLAT300_ALERT_4_G Atom Site Occupancy of H0_A:13 Constrained at 0.4879 Check
PLAT300_ALERT_4_G Atom Site Occupancy of Ha_A:13 Constrained at 0.4879 Check
PLAT300_ALERT_4_G Atom Site Occupancy of Hb1_A:13 Constrained at 0.4879 Check
PLAT300_ALERT_4_G Atom Site Occupancy of Hb2_A:13 Constrained at 0.4879 Check
PLAT300_ALERT_4_G Atom Site Occupancy of Hd2_A:13 Constrained at 0.4879 Check
PLAT300_ALERT_4_G Atom Site Occupancy of Hd1_A:13 Constrained at 0.4879 Check
PLAT300_ALERT_4_G Atom Site Occupancy of He1_A:13 Constrained at 0.4879 Check
PLAT300_ALERT_4_G Atom Site Occupancy of He2_A:13 Constrained at 0.4879 Check
PLAT300_ALERT_4_G Atom Site Occupancy of Hz_A:13 Constrained at 0.4879 Check
PLAT300_ALERT_4_G Atom Site Occupancy of Ha1_A:19 Constrained at 0.3304 Check
PLAT300_ALERT_4_G Atom Site Occupancy of Hb3_A:19 Constrained at 0.3304 Check
PLAT300_ALERT_4_G Atom Site Occupancy of Hb4_A:19 Constrained at 0.3304 Check
PLAT300_ALERT_4_G Atom Site Occupancy of Hg3_A:19 Constrained at 0.3304 Check
PLAT300_ALERT_4_G Atom Site Occupancy of Hg4_A:19 Constrained at 0.3304 Check
PLAT300_ALERT_4_G Atom Site Occupancy of Hd3_A:19 Constrained at 0.3304 Check
PLAT300_ALERT_4_G Atom Site Occupancy of Hd4_A:19 Constrained at 0.3304 Check
PLAT300_ALERT_4_G Atom Site Occupancy of Ha_A:22 Constrained at 0.3101 Check
PLAT300_ALERT_4_G Atom Site Occupancy of Hb1_A:22 Constrained at 0.3101 Check
PLAT300_ALERT_4_G Atom Site Occupancy of Hb2_A:22 Constrained at 0.3101 Check
PLAT300_ALERT_4_G Atom Site Occupancy of Ha1_A:22 Constrained at 0.3589 Check
PLAT300_ALERT_4_G Atom Site Occupancy of Hb3_A:22 Constrained at 0.3589 Check
PLAT300_ALERT_4_G Atom Site Occupancy of Hb4_A:22 Constrained at 0.3589 Check
PLAT300_ALERT_4_G Atom Site Occupancy of Hg1_A:22 Constrained at 0.3589 Check
PLAT300_ALERT_4_G Atom Site Occupancy of Hg2_A:22 Constrained at 0.3589 Check
PLAT300_ALERT_4_G Atom Site Occupancy of Hd1_A:22 Constrained at 0.3589 Check
PLAT300_ALERT_4_G Atom Site Occupancy of Hd2_A:22 Constrained at 0.3589 Check
PLAT300_ALERT_4_G Atom Site Occupancy of Ha2_A:22 Constrained at 0.331 Check
PLAT300_ALERT_4_G Atom Site Occupancy of Hb5_A:22 Constrained at 0.331 Check
PLAT300_ALERT_4_G Atom Site Occupancy of Hb6_A:22 Constrained at 0.331 Check
PLAT300_ALERT_4_G Atom Site Occupancy of H0_A:25 Constrained at 0.3542 Check
PLAT300_ALERT_4_G Atom Site Occupancy of HaX_A:25 Constrained at 0.3542 Check
PLAT300_ALERT_4_G Atom Site Occupancy of HbX_A:25 Constrained at 0.3542 Check
PLAT300_ALERT_4_G Atom Site Occupancy of Hg1X_A:25 Constrained at 0.3542 Check
PLAT300_ALERT_4_G Atom Site Occupancy of Hg1Y_A:25 Constrained at 0.3542 Check
PLAT300_ALERT_4_G Atom Site Occupancy of Hg2X_A:25 Constrained at 0.3542 Check
PLAT300_ALERT_4_G Atom Site Occupancy of Hg2Y_A:25 Constrained at 0.3542 Check
PLAT300_ALERT_4_G Atom Site Occupancy of Hg2Z_A:25 Constrained at 0.3542 Check
PLAT300_ALERT_4_G Atom Site Occupancy of Hd1X_A:25 Constrained at 0.3542 Check
PLAT300_ALERT_4_G Atom Site Occupancy of Hd1Y_A:25 Constrained at 0.3542 Check
PLAT300_ALERT_4_G Atom Site Occupancy of Hd1Z_A:25 Constrained at 0.3542 Check
PLAT300_ALERT_4_G Atom Site Occupancy of Hy_A:25 Constrained at 0.3589 Check
PLAT300_ALERT_4_G Atom Site Occupancy of HaY_A:25 Constrained at 0.3589 Check
PLAT300_ALERT_4_G Atom Site Occupancy of Hb2_A:25 Constrained at 0.3589 Check
PLAT300_ALERT_4_G Atom Site Occupancy of Hb3_A:25 Constrained at 0.3589 Check
PLAT300_ALERT_4_G Atom Site Occupancy of Hd11_A:25 Constrained at 0.3589 Check
PLAT300_ALERT_4_G Atom Site Occupancy of Hd12_A:25 Constrained at 0.3589 Check
PLAT300_ALERT_4_G Atom Site Occupancy of Hd13_A:25 Constrained at 0.3589 Check
PLAT300_ALERT_4_G Atom Site Occupancy of Hd21_A:25 Constrained at 0.3589 Check
PLAT300_ALERT_4_G Atom Site Occupancy of Hd22_A:25 Constrained at 0.3589 Check
PLAT300_ALERT_4_G Atom Site Occupancy of Hd23_A:25 Constrained at 0.3589 Check
PLAT300_ALERT_4_G Atom Site Occupancy of Hg_A:25 Constrained at 0.3589 Check
PLAT300_ALERT_4_G Atom Site Occupancy of H_A:25 Constrained at 0.2869 Check
PLAT300_ALERT_4_G Atom Site Occupancy of Ha_A:25 Constrained at 0.2869 Check
PLAT300_ALERT_4_G Atom Site Occupancy of Hb_A:25 Constrained at 0.2869 Check
PLAT300_ALERT_4_G Atom Site Occupancy of Hg1A_A:25 Constrained at 0.2869 Check
PLAT300_ALERT_4_G Atom Site Occupancy of Hg1B_A:25 Constrained at 0.2869 Check
PLAT300_ALERT_4_G Atom Site Occupancy of Hg2A_A:25 Constrained at 0.2869 Check

```
PLAT300_ALERT_4_G Atom Site Occupancy of Hg2B_A:25  Constrained at     0.2869 Check
PLAT300_ALERT_4_G Atom Site Occupancy of Hg2C_A:25  Constrained at     0.2869 Check
PLAT300_ALERT_4_G Atom Site Occupancy of Hd1A_A:25  Constrained at     0.2869 Check
PLAT300_ALERT_4_G Atom Site Occupancy of Hd1B_A:25  Constrained at     0.2869 Check
PLAT300_ALERT_4_G Atom Site Occupancy of Hd1C_A:25  Constrained at     0.2869 Check
PLAT300_ALERT_4_G Atom Site Occupancy of H0_A:29    Constrained at     0.3589 Check
PLAT300_ALERT_4_G Atom Site Occupancy of Ha_A:29    Constrained at     0.3589 Check
PLAT300_ALERT_4_G Atom Site Occupancy of Hb1_A:29   Constrained at     0.3589 Check
PLAT300_ALERT_4_G Atom Site Occupancy of Hb2_A:29   Constrained at     0.3589 Check
PLAT300_ALERT_4_G Atom Site Occupancy of Hd1_A:29   Constrained at     0.3589 Check
PLAT300_ALERT_4_G Atom Site Occupancy of Hd2_A:29   Constrained at     0.3589 Check
PLAT300_ALERT_4_G Atom Site Occupancy of He1_A:29   Constrained at     0.3589 Check
PLAT300_ALERT_4_G Atom Site Occupancy of He2_A:29   Constrained at     0.3589 Check
PLAT300_ALERT_4_G Atom Site Occupancy of Hh_A:29    Constrained at     0.3589 Check
PLAT300_ALERT_4_G Atom Site Occupancy of H0A_A:29   Constrained at     0.2274 Check
PLAT300_ALERT_4_G Atom Site Occupancy of Ha1_A:29   Constrained at     0.2274 Check
PLAT300_ALERT_4_G Atom Site Occupancy of Hb3_A:29   Constrained at     0.2274 Check
PLAT300_ALERT_4_G Atom Site Occupancy of Hb4_A:29   Constrained at     0.2274 Check
PLAT300_ALERT_4_G Atom Site Occupancy of Hd1A_A:29  Constrained at     0.2274 Check
PLAT300_ALERT_4_G Atom Site Occupancy of Hd2A_A:29  Constrained at     0.2274 Check
PLAT300_ALERT_4_G Atom Site Occupancy of He1A_A:29  Constrained at     0.2274 Check
PLAT300_ALERT_4_G Atom Site Occupancy of He2A_A:29  Constrained at     0.2274 Check
PLAT300_ALERT_4_G Atom Site Occupancy of Hh1_A:29   Constrained at     0.2274 Check
PLAT300_ALERT_4_G Atom Site Occupancy of H0B_A:29   Constrained at     0.4137 Check
PLAT300_ALERT_4_G Atom Site Occupancy of Ha2_A:29   Constrained at     0.4137 Check
PLAT300_ALERT_4_G Atom Site Occupancy of Hb5_A:29   Constrained at     0.4137 Check
PLAT300_ALERT_4_G Atom Site Occupancy of Hb6_A:29   Constrained at     0.4137 Check
PLAT300_ALERT_4_G Atom Site Occupancy of Hd1B_A:29  Constrained at     0.4137 Check
PLAT300_ALERT_4_G Atom Site Occupancy of Hd2B_A:29  Constrained at     0.4137 Check
PLAT300_ALERT_4_G Atom Site Occupancy of He2B_A:29  Constrained at     0.4137 Check
PLAT300_ALERT_4_G Atom Site Occupancy of He1B_A:29  Constrained at     0.4137 Check
PLAT300_ALERT_4_G Atom Site Occupancy of Hh2_A:29   Constrained at     0.4137 Check
PLAT300_ALERT_4_G Atom Site Occupancy of Hd1D_A:34  Constrained at     0.2218 Check
PLAT300_ALERT_4_G Atom Site Occupancy of Hd1E_A:34  Constrained at     0.2218 Check
PLAT300_ALERT_4_G Atom Site Occupancy of Hd1F_A:34  Constrained at     0.2218 Check
PLAT300_ALERT_4_G Atom Site Occupancy of H0_A:37    Constrained at     0.4837 Check
PLAT300_ALERT_4_G Atom Site Occupancy of Ha1_A:37   Constrained at     0.4837 Check
PLAT300_ALERT_4_G Atom Site Occupancy of Ha2_A:37   Constrained at     0.4837 Check
PLAT300_ALERT_4_G Atom Site Occupancy of H0A_A:39   Constrained at      0.363 Check
PLAT300_ALERT_4_G Atom Site Occupancy of Ha1_A:39   Constrained at      0.363 Check
PLAT300_ALERT_4_G Atom Site Occupancy of Hb1_A:39   Constrained at      0.363 Check
PLAT300_ALERT_4_G Atom Site Occupancy of Hg2D_A:39  Constrained at      0.363 Check
PLAT300_ALERT_4_G Atom Site Occupancy of Hg2E_A:39  Constrained at      0.363 Check
PLAT300_ALERT_4_G Atom Site Occupancy of Hg2F_A:39  Constrained at      0.363 Check
PLAT300_ALERT_4_G Atom Site Occupancy of Hg1A_A:39  Constrained at      0.363 Check
PLAT300_ALERT_4_G Atom Site Occupancy of H0A_A:43   Constrained at     0.4834 Check
PLAT300_ALERT_4_G Atom Site Occupancy of Ha1_A:43   Constrained at     0.4834 Check
PLAT300_ALERT_4_G Atom Site Occupancy of Hb3_A:43   Constrained at     0.4834 Check
PLAT300_ALERT_4_G Atom Site Occupancy of Hb4_A:43   Constrained at     0.4834 Check
PLAT300_ALERT_4_G Atom Site Occupancy of Ox_S:29    Constrained at     0.3486 Check
PLAT300_ALERT_4_G Atom Site Occupancy of Ox_S:30    Constrained at      0.431 Check
PLAT300_ALERT_4_G Atom Site Occupancy of O_S:32     Constrained at      0.467 Check
PLAT300_ALERT_4_G Atom Site Occupancy of O_S:33     Constrained at     0.2519 Check
PLAT300_ALERT_4_G Atom Site Occupancy of O_S:44     Constrained at     0.3224 Check
PLAT300_ALERT_4_G Atom Site Occupancy of O_S:49     Constrained at      0.422 Check
PLAT300_ALERT_4_G Atom Site Occupancy of Ox_S:49    Constrained at     0.2953 Check
PLAT300_ALERT_4_G Atom Site Occupancy of O_S:52     Constrained at     0.2636 Check
```

```
PLAT300_ALERT_4_G Atom Site Occupancy of Ox_S:53    Constrained at      0.344 Check
PLAT300_ALERT_4_G Atom Site Occupancy of O_S:56     Constrained at     0.4972 Check
PLAT300_ALERT_4_G Atom Site Occupancy of O_S:57     Constrained at     0.3139 Check
PLAT300_ALERT_4_G Atom Site Occupancy of Ox_S:57    Constrained at     0.2726 Check
PLAT300_ALERT_4_G Atom Site Occupancy of Oy_S:57    Constrained at     0.4136 Check
PLAT300_ALERT_4_G Atom Site Occupancy of O_S:69     Constrained at     0.3975 Check
PLAT300_ALERT_4_G Atom Site Occupancy of C2_A:102   Constrained at     0.8274 Check
PLAT300_ALERT_4_G Atom Site Occupancy of C1_A:102   Constrained at     0.4326 Check
PLAT300_ALERT_4_G Atom Site Occupancy of O_A:101    Constrained at     0.5839 Check
PLAT300_ALERT_4_G Atom Site Occupancy of C1_A:101   Constrained at     0.5839 Check
PLAT300_ALERT_4_G Atom Site Occupancy of C2_A:101   Constrained at     0.5839 Check
PLAT300_ALERT_4_G Atom Site Occupancy of Ox_A:101   Constrained at     0.4161 Check
PLAT300_ALERT_4_G Atom Site Occupancy of C1X_A:101  Constrained at     0.4161 Check
PLAT300_ALERT_4_G Atom Site Occupancy of C2X_A:101  Constrained at     0.4161 Check
PLAT300_ALERT_4_G Atom Site Occupancy of Ox_S:46    Constrained at     0.3415 Check
PLAT300_ALERT_4_G Atom Site Occupancy of O_A:102    Constrained at     0.8178 Check
PLAT300_ALERT_4_G Atom Site Occupancy of O_S:2      Constrained at     0.9247 Check
PLAT300_ALERT_4_G Atom Site Occupancy of H2_S:2     Constrained at     0.9247 Check
PLAT300_ALERT_4_G Atom Site Occupancy of H1_S:2     Constrained at     0.9247 Check
PLAT300_ALERT_4_G Atom Site Occupancy of O_S:5      Constrained at     0.9211 Check
PLAT300_ALERT_4_G Atom Site Occupancy of H2_S:5     Constrained at     0.9211 Check
PLAT300_ALERT_4_G Atom Site Occupancy of H1_S:5     Constrained at     0.9211 Check
PLAT300_ALERT_4_G Atom Site Occupancy of O_S:6      Constrained at     0.7977 Check
PLAT300_ALERT_4_G Atom Site Occupancy of H2_S:6     Constrained at     0.7977 Check
PLAT300_ALERT_4_G Atom Site Occupancy of H1_S:6     Constrained at     0.7977 Check
PLAT300_ALERT_4_G Atom Site Occupancy of O_S:8      Constrained at     0.6323 Check
PLAT300_ALERT_4_G Atom Site Occupancy of O_S:9      Constrained at     0.9502 Check
PLAT300_ALERT_4_G Atom Site Occupancy of H2_S:9     Constrained at     0.9502 Check
PLAT300_ALERT_4_G Atom Site Occupancy of H1_S:9     Constrained at     0.9502 Check
PLAT300_ALERT_4_G Atom Site Occupancy of O_S:10     Constrained at     0.8598 Check
PLAT300_ALERT_4_G Atom Site Occupancy of O_S:11     Constrained at     0.6634 Check
PLAT300_ALERT_4_G Atom Site Occupancy of O_S:12     Constrained at     0.7459 Check
PLAT300_ALERT_4_G Atom Site Occupancy of H2_S:12    Constrained at     0.7459 Check
PLAT300_ALERT_4_G Atom Site Occupancy of H1_S:12    Constrained at     0.7459 Check
PLAT300_ALERT_4_G Atom Site Occupancy of O_S:15     Constrained at     0.7436 Check
PLAT300_ALERT_4_G Atom Site Occupancy of H2_S:15    Constrained at     0.7436 Check
PLAT300_ALERT_4_G Atom Site Occupancy of H1_S:15    Constrained at     0.7436 Check
PLAT300_ALERT_4_G Atom Site Occupancy of Ox_S:16    Constrained at     0.5346 Check
PLAT300_ALERT_4_G Atom Site Occupancy of O_S:17     Constrained at     0.8644 Check
PLAT300_ALERT_4_G Atom Site Occupancy of H2_S:17    Constrained at     0.8644 Check
PLAT300_ALERT_4_G Atom Site Occupancy of H1_S:17    Constrained at     0.8644 Check
PLAT300_ALERT_4_G Atom Site Occupancy of O_S:18     Constrained at     0.6159 Check
PLAT300_ALERT_4_G Atom Site Occupancy of O_S:19     Constrained at     0.6681 Check
PLAT300_ALERT_4_G Atom Site Occupancy of H2_S:19    Constrained at     0.6681 Check
PLAT300_ALERT_4_G Atom Site Occupancy of H1_S:19    Constrained at     0.6681 Check
PLAT300_ALERT_4_G Atom Site Occupancy of O_S:20     Constrained at     0.8008 Check
PLAT300_ALERT_4_G Atom Site Occupancy of H2_S:20    Constrained at     0.8008 Check
PLAT300_ALERT_4_G Atom Site Occupancy of H1_S:20    Constrained at     0.8008 Check
PLAT300_ALERT_4_G Atom Site Occupancy of O_S:21     Constrained at     0.6616 Check
PLAT300_ALERT_4_G Atom Site Occupancy of O_S:23     Constrained at     0.6777 Check
PLAT300_ALERT_4_G Atom Site Occupancy of O_S:24     Constrained at     0.6468 Check
PLAT300_ALERT_4_G Atom Site Occupancy of O_S:25     Constrained at     0.7036 Check
PLAT300_ALERT_4_G Atom Site Occupancy of O_S:26     Constrained at     0.5945 Check
PLAT300_ALERT_4_G Atom Site Occupancy of O_S:27     Constrained at     0.8293 Check
PLAT300_ALERT_4_G Atom Site Occupancy of O_S:29     Constrained at     0.5184 Check
PLAT300_ALERT_4_G Atom Site Occupancy of O_S:30     Constrained at     0.5691 Check
PLAT300_ALERT_4_G Atom Site Occupancy of Ox_S:32    Constrained at      0.533 Check
```

```
PLAT300_ALERT_4_G Atom Site Occupancy of Ox_S:33    Constrained at     0.7142 Check
PLAT300_ALERT_4_G Atom Site Occupancy of O_S:34     Constrained at     0.7442 Check
PLAT300_ALERT_4_G Atom Site Occupancy of O_S:35     Constrained at     0.8498 Check
PLAT300_ALERT_4_G Atom Site Occupancy of O_S:36     Constrained at     0.7809 Check
PLAT300_ALERT_4_G Atom Site Occupancy of O_S:37     Constrained at     0.8438 Check
PLAT300_ALERT_4_G Atom Site Occupancy of O_S:38     Constrained at     0.6244 Check
PLAT300_ALERT_4_G Atom Site Occupancy of O_S:39     Constrained at     0.7308 Check
PLAT300_ALERT_4_G Atom Site Occupancy of O_S:40     Constrained at     0.7692 Check
PLAT300_ALERT_4_G Atom Site Occupancy of O_S:41     Constrained at     0.5419 Check
PLAT300_ALERT_4_G Atom Site Occupancy of O_S:42     Constrained at     0.7476 Check
PLAT300_ALERT_4_G Atom Site Occupancy of Ox_S:44    Constrained at     0.6776 Check
PLAT300_ALERT_4_G Atom Site Occupancy of O_S:46     Constrained at     0.6585 Check
PLAT300_ALERT_4_G Atom Site Occupancy of O_S:47     Constrained at     0.7995 Check
PLAT300_ALERT_4_G Atom Site Occupancy of O_S:51     Constrained at     0.7364 Check
PLAT300_ALERT_4_G Atom Site Occupancy of O_S:53     Constrained at      0.656 Check
PLAT300_ALERT_4_G Atom Site Occupancy of Ox_S:8     Constrained at     0.3677 Check
PLAT300_ALERT_4_G Atom Site Occupancy of Ox_S:11    Constrained at     0.3366 Check
PLAT300_ALERT_4_G Atom Site Occupancy of O_S:16     Constrained at     0.4045 Check
PLAT300_ALERT_4_G Atom Site Occupancy of Ox_S:18    Constrained at     0.3841 Check
PLAT300_ALERT_4_G Atom Site Occupancy of O_S:22     Constrained at     0.3904 Check
PLAT300_ALERT_4_G Atom Site Occupancy of Ox_S:22    Constrained at     0.2863 Check
PLAT300_ALERT_4_G Atom Site Occupancy of Ox_S:26    Constrained at     0.4055 Check
PLAT301_ALERT_3_G Main Residue  Disorder ..............(Resd    1)        34% Note
PLAT302_ALERT_4_G Anion/Solvent/Minor-Residue Disorder (Resd    2)       100% Note
PLAT302_ALERT_4_G Anion/Solvent/Minor-Residue Disorder (Resd    3)       100% Note
PLAT302_ALERT_4_G Anion/Solvent/Minor-Residue Disorder (Resd    4)       100% Note
PLAT302_ALERT_4_G Anion/Solvent/Minor-Residue Disorder (Resd    5)        25% Note
PLAT302_ALERT_4_G Anion/Solvent/Minor-Residue Disorder (Resd    6)       100% Note
PLAT302_ALERT_4_G Anion/Solvent/Minor-Residue Disorder (Resd    8)       100% Note
PLAT302_ALERT_4_G Anion/Solvent/Minor-Residue Disorder (Resd   11)       100% Note
PLAT302_ALERT_4_G Anion/Solvent/Minor-Residue Disorder (Resd   12)       100% Note
PLAT302_ALERT_4_G Anion/Solvent/Minor-Residue Disorder (Resd   14)       100% Note
PLAT302_ALERT_4_G Anion/Solvent/Minor-Residue Disorder (Resd   15)       100% Note
PLAT302_ALERT_4_G Anion/Solvent/Minor-Residue Disorder (Resd   16)       100% Note
PLAT302_ALERT_4_G Anion/Solvent/Minor-Residue Disorder (Resd   17)       100% Note
PLAT302_ALERT_4_G Anion/Solvent/Minor-Residue Disorder (Resd   18)       100% Note
PLAT302_ALERT_4_G Anion/Solvent/Minor-Residue Disorder (Resd   21)       100% Note
PLAT302_ALERT_4_G Anion/Solvent/Minor-Residue Disorder (Resd   22)       100% Note
PLAT302_ALERT_4_G Anion/Solvent/Minor-Residue Disorder (Resd   23)       100% Note
PLAT302_ALERT_4_G Anion/Solvent/Minor-Residue Disorder (Resd   24)       100% Note
PLAT302_ALERT_4_G Anion/Solvent/Minor-Residue Disorder (Resd   25)       100% Note
PLAT302_ALERT_4_G Anion/Solvent/Minor-Residue Disorder (Resd   26)       100% Note
PLAT302_ALERT_4_G Anion/Solvent/Minor-Residue Disorder (Resd   27)       100% Note
PLAT302_ALERT_4_G Anion/Solvent/Minor-Residue Disorder (Resd   28)       100% Note
PLAT302_ALERT_4_G Anion/Solvent/Minor-Residue Disorder (Resd   29)       100% Note
PLAT302_ALERT_4_G Anion/Solvent/Minor-Residue Disorder (Resd   30)       100% Note
PLAT302_ALERT_4_G Anion/Solvent/Minor-Residue Disorder (Resd   31)       100% Note
PLAT302_ALERT_4_G Anion/Solvent/Minor-Residue Disorder (Resd   32)       100% Note
PLAT302_ALERT_4_G Anion/Solvent/Minor-Residue Disorder (Resd   33)       100% Note
PLAT302_ALERT_4_G Anion/Solvent/Minor-Residue Disorder (Resd   34)       100% Note
PLAT302_ALERT_4_G Anion/Solvent/Minor-Residue Disorder (Resd   36)       100% Note
PLAT302_ALERT_4_G Anion/Solvent/Minor-Residue Disorder (Resd   37)       100% Note
PLAT302_ALERT_4_G Anion/Solvent/Minor-Residue Disorder (Resd   38)       100% Note
PLAT302_ALERT_4_G Anion/Solvent/Minor-Residue Disorder (Resd   39)       100% Note
PLAT302_ALERT_4_G Anion/Solvent/Minor-Residue Disorder (Resd   40)       100% Note
PLAT302_ALERT_4_G Anion/Solvent/Minor-Residue Disorder (Resd   41)       100% Note
PLAT302_ALERT_4_G Anion/Solvent/Minor-Residue Disorder (Resd   42)       100% Note
```

```
PLAT302_ALERT_4_G Anion/Solvent/Minor-Residue Disorder (Resd   43)       100% Note
PLAT302_ALERT_4_G Anion/Solvent/Minor-Residue Disorder (Resd   44)       100% Note
PLAT302_ALERT_4_G Anion/Solvent/Minor-Residue Disorder (Resd   45)       100% Note
PLAT302_ALERT_4_G Anion/Solvent/Minor-Residue Disorder (Resd   46)       100% Note
PLAT302_ALERT_4_G Anion/Solvent/Minor-Residue Disorder (Resd   47)       100% Note
PLAT302_ALERT_4_G Anion/Solvent/Minor-Residue Disorder (Resd   49)       100% Note
PLAT302_ALERT_4_G Anion/Solvent/Minor-Residue Disorder (Resd   50)       100% Note
PLAT302_ALERT_4_G Anion/Solvent/Minor-Residue Disorder (Resd   53)       100% Note
PLAT302_ALERT_4_G Anion/Solvent/Minor-Residue Disorder (Resd   54)       100% Note
PLAT302_ALERT_4_G Anion/Solvent/Minor-Residue Disorder (Resd   57)       100% Note
PLAT302_ALERT_4_G Anion/Solvent/Minor-Residue Disorder (Resd   58)       100% Note
PLAT302_ALERT_4_G Anion/Solvent/Minor-Residue Disorder (Resd   59)       100% Note
PLAT302_ALERT_4_G Anion/Solvent/Minor-Residue Disorder (Resd   60)       100% Note
PLAT302_ALERT_4_G Anion/Solvent/Minor-Residue Disorder (Resd   61)       100% Note
PLAT302_ALERT_4_G Anion/Solvent/Minor-Residue Disorder (Resd   62)       100% Note
PLAT302_ALERT_4_G Anion/Solvent/Minor-Residue Disorder (Resd   63)       100% Note
PLAT304_ALERT_4_G Non-Integer Number of Atoms in ..... (Resd    1)     637.56 Check
PLAT304_ALERT_4_G Non-Integer Number of Atoms in ..... (Resd    2)       6.30 Check
PLAT304_ALERT_4_G Non-Integer Number of Atoms in ..... (Resd    3)       1.75 Check
PLAT304_ALERT_4_G Non-Integer Number of Atoms in ..... (Resd    4)       1.25 Check
PLAT304_ALERT_4_G Non-Integer Number of Atoms in ..... (Resd    5)       1.34 Check
PLAT304_ALERT_4_G Non-Integer Number of Atoms in ..... (Resd    6)       0.82 Check
PLAT304_ALERT_4_G Non-Integer Number of Atoms in ..... (Resd    8)       2.77 Check
PLAT304_ALERT_4_G Non-Integer Number of Atoms in ..... (Resd   11)       2.76 Check
PLAT304_ALERT_4_G Non-Integer Number of Atoms in ..... (Resd   12)       2.39 Check
PLAT304_ALERT_4_G Non-Integer Number of Atoms in ..... (Resd   14)       0.63 Check
PLAT304_ALERT_4_G Non-Integer Number of Atoms in ..... (Resd   15)       2.85 Check
PLAT304_ALERT_4_G Non-Integer Number of Atoms in ..... (Resd   16)       0.86 Check
PLAT304_ALERT_4_G Non-Integer Number of Atoms in ..... (Resd   17)       0.66 Check
PLAT304_ALERT_4_G Non-Integer Number of Atoms in ..... (Resd   18)       2.24 Check
PLAT304_ALERT_4_G Non-Integer Number of Atoms in ..... (Resd   21)       2.23 Check
PLAT304_ALERT_4_G Non-Integer Number of Atoms in ..... (Resd   22)       0.53 Check
PLAT304_ALERT_4_G Non-Integer Number of Atoms in ..... (Resd   23)       2.59 Check
PLAT304_ALERT_4_G Non-Integer Number of Atoms in ..... (Resd   24)       0.62 Check
PLAT304_ALERT_4_G Non-Integer Number of Atoms in ..... (Resd   26)       2.40 Check
PLAT304_ALERT_4_G Non-Integer Number of Atoms in ..... (Resd   27)       0.66 Check
PLAT304_ALERT_4_G Non-Integer Number of Atoms in ..... (Resd   28)       0.68 Check
PLAT304_ALERT_4_G Non-Integer Number of Atoms in ..... (Resd   29)       0.65 Check
PLAT304_ALERT_4_G Non-Integer Number of Atoms in ..... (Resd   30)       0.70 Check
PLAT304_ALERT_4_G Non-Integer Number of Atoms in ..... (Resd   31)       0.59 Check
PLAT304_ALERT_4_G Non-Integer Number of Atoms in ..... (Resd   32)       0.83 Check
PLAT304_ALERT_4_G Non-Integer Number of Atoms in ..... (Resd   33)       0.52 Check
PLAT304_ALERT_4_G Non-Integer Number of Atoms in ..... (Resd   34)       0.57 Check
PLAT304_ALERT_4_G Non-Integer Number of Atoms in ..... (Resd   36)       0.53 Check
PLAT304_ALERT_4_G Non-Integer Number of Atoms in ..... (Resd   37)       0.71 Check
PLAT304_ALERT_4_G Non-Integer Number of Atoms in ..... (Resd   38)       0.74 Check
PLAT304_ALERT_4_G Non-Integer Number of Atoms in ..... (Resd   39)       0.85 Check
PLAT304_ALERT_4_G Non-Integer Number of Atoms in ..... (Resd   40)       0.78 Check
PLAT304_ALERT_4_G Non-Integer Number of Atoms in ..... (Resd   41)       0.84 Check
PLAT304_ALERT_4_G Non-Integer Number of Atoms in ..... (Resd   42)       0.62 Check
PLAT304_ALERT_4_G Non-Integer Number of Atoms in ..... (Resd   43)       0.73 Check
PLAT304_ALERT_4_G Non-Integer Number of Atoms in ..... (Resd   44)       0.77 Check
PLAT304_ALERT_4_G Non-Integer Number of Atoms in ..... (Resd   45)       0.54 Check
PLAT304_ALERT_4_G Non-Integer Number of Atoms in ..... (Resd   46)       0.75 Check
PLAT304_ALERT_4_G Non-Integer Number of Atoms in ..... (Resd   47)       0.68 Check
PLAT304_ALERT_4_G Non-Integer Number of Atoms in ..... (Resd   49)       0.66 Check
PLAT304_ALERT_4_G Non-Integer Number of Atoms in ..... (Resd   50)       0.80 Check
```

```
PLAT304_ALERT_4_G Non-Integer Number of Atoms in ..... (Resd   53)       0.74 Check
PLAT304_ALERT_4_G Non-Integer Number of Atoms in ..... (Resd   54)       0.66 Check
PLAT304_ALERT_4_G Non-Integer Number of Atoms in ..... (Resd   57)       0.37 Check
PLAT304_ALERT_4_G Non-Integer Number of Atoms in ..... (Resd   58)       0.34 Check
PLAT304_ALERT_4_G Non-Integer Number of Atoms in ..... (Resd   59)       0.40 Check
PLAT304_ALERT_4_G Non-Integer Number of Atoms in ..... (Resd   60)       0.38 Check
PLAT304_ALERT_4_G Non-Integer Number of Atoms in ..... (Resd   61)       0.39 Check
PLAT304_ALERT_4_G Non-Integer Number of Atoms in ..... (Resd   62)       0.29 Check
PLAT304_ALERT_4_G Non-Integer Number of Atoms in ..... (Resd   63)       0.41 Check
PLAT311_ALERT_2_G Isolated Disordered Oxygen Atom (No H's ?) .....    Ox_S:29 Check
PLAT311_ALERT_2_G Isolated Disordered Oxygen Atom (No H's ?) .....    Ox_S:30 Check
PLAT311_ALERT_2_G Isolated Disordered Oxygen Atom (No H's ?) .....     O_S:32 Check
PLAT311_ALERT_2_G Isolated Disordered Oxygen Atom (No H's ?) .....     O_S:33 Check
PLAT311_ALERT_2_G Isolated Disordered Oxygen Atom (No H's ?) .....     O_S:44 Check
PLAT311_ALERT_2_G Isolated Disordered Oxygen Atom (No H's ?) .....     O_S:49 Check
PLAT311_ALERT_2_G Isolated Disordered Oxygen Atom (No H's ?) .....    Ox_S:49 Check
PLAT311_ALERT_2_G Isolated Disordered Oxygen Atom (No H's ?) .....     O_S:52 Check
PLAT311_ALERT_2_G Isolated Disordered Oxygen Atom (No H's ?) .....    Ox_S:53 Check
PLAT311_ALERT_2_G Isolated Disordered Oxygen Atom (No H's ?) .....     O_S:56 Check
PLAT311_ALERT_2_G Isolated Disordered Oxygen Atom (No H's ?) .....     O_S:57 Check
PLAT311_ALERT_2_G Isolated Disordered Oxygen Atom (No H's ?) .....    Ox_S:57 Check
PLAT311_ALERT_2_G Isolated Disordered Oxygen Atom (No H's ?) .....    Oy_S:57 Check
PLAT311_ALERT_2_G Isolated Disordered Oxygen Atom (No H's ?) .....     O_S:69 Check
PLAT311_ALERT_2_G Isolated Disordered Oxygen Atom (No H's ?) .....    O_A:102 Check
PLAT311_ALERT_2_G Isolated Disordered Oxygen Atom (No H's ?) .....      O_S:8 Check
PLAT311_ALERT_2_G Isolated Disordered Oxygen Atom (No H's ?) .....     O_S:10 Check
PLAT311_ALERT_2_G Isolated Disordered Oxygen Atom (No H's ?) .....     O_S:11 Check
PLAT311_ALERT_2_G Isolated Disordered Oxygen Atom (No H's ?) .....    Ox_S:16 Check
PLAT311_ALERT_2_G Isolated Disordered Oxygen Atom (No H's ?) .....     O_S:18 Check
PLAT311_ALERT_2_G Isolated Disordered Oxygen Atom (No H's ?) .....     O_S:21 Check
PLAT311_ALERT_2_G Isolated Disordered Oxygen Atom (No H's ?) .....     O_S:23 Check
PLAT311_ALERT_2_G Isolated Disordered Oxygen Atom (No H's ?) .....     O_S:24 Check
PLAT311_ALERT_2_G Isolated Disordered Oxygen Atom (No H's ?) .....     O_S:25 Check
PLAT311_ALERT_2_G Isolated Disordered Oxygen Atom (No H's ?) .....     O_S:26 Check
PLAT311_ALERT_2_G Isolated Disordered Oxygen Atom (No H's ?) .....     O_S:27 Check
PLAT311_ALERT_2_G Isolated Disordered Oxygen Atom (No H's ?) .....     O_S:29 Check
PLAT311_ALERT_2_G Isolated Disordered Oxygen Atom (No H's ?) .....     O_S:30 Check
PLAT311_ALERT_2_G Isolated Disordered Oxygen Atom (No H's ?) .....    Ox_S:32 Check
PLAT311_ALERT_2_G Isolated Disordered Oxygen Atom (No H's ?) .....    Ox_S:33 Check
PLAT311_ALERT_2_G Isolated Disordered Oxygen Atom (No H's ?) .....     O_S:34 Check
PLAT311_ALERT_2_G Isolated Disordered Oxygen Atom (No H's ?) .....     O_S:35 Check
PLAT311_ALERT_2_G Isolated Disordered Oxygen Atom (No H's ?) .....     O_S:36 Check
PLAT311_ALERT_2_G Isolated Disordered Oxygen Atom (No H's ?) .....     O_S:37 Check
PLAT311_ALERT_2_G Isolated Disordered Oxygen Atom (No H's ?) .....     O_S:38 Check
PLAT311_ALERT_2_G Isolated Disordered Oxygen Atom (No H's ?) .....     O_S:39 Check
PLAT311_ALERT_2_G Isolated Disordered Oxygen Atom (No H's ?) .....     O_S:40 Check
PLAT311_ALERT_2_G Isolated Disordered Oxygen Atom (No H's ?) .....     O_S:41 Check
PLAT311_ALERT_2_G Isolated Disordered Oxygen Atom (No H's ?) .....     O_S:42 Check
PLAT311_ALERT_2_G Isolated Disordered Oxygen Atom (No H's ?) .....    Ox_S:44 Check
PLAT311_ALERT_2_G Isolated Disordered Oxygen Atom (No H's ?) .....     O_S:46 Check
PLAT311_ALERT_2_G Isolated Disordered Oxygen Atom (No H's ?) .....     O_S:47 Check
PLAT311_ALERT_2_G Isolated Disordered Oxygen Atom (No H's ?) .....     O_S:51 Check
PLAT311_ALERT_2_G Isolated Disordered Oxygen Atom (No H's ?) .....     O_S:53 Check
PLAT311_ALERT_2_G Isolated Disordered Oxygen Atom (No H's ?) .....     Ox_S:8 Check
PLAT311_ALERT_2_G Isolated Disordered Oxygen Atom (No H's ?) .....    Ox_S:11 Check
PLAT311_ALERT_2_G Isolated Disordered Oxygen Atom (No H's ?) .....     O_S:16 Check
PLAT311_ALERT_2_G Isolated Disordered Oxygen Atom (No H's ?) .....    Ox_S:18 Check
```

```
PLAT311_ALERT_2_G Isolated Disordered Oxygen Atom (No H's ?) .....     O_S:22 Check
PLAT311_ALERT_2_G Isolated Disordered Oxygen Atom (No H's ?) .....    Ox_S:22 Check
PLAT311_ALERT_2_G Isolated Disordered Oxygen Atom (No H's ?) .....    Ox_S:26 Check
PLAT315_ALERT_2_G Singly Bonded Carbon Detected (H-atoms Missing).   C2_A:101 Check
PLAT315_ALERT_2_G Singly Bonded Carbon Detected (H-atoms Missing).  C2X_A:101 Check
PLAT410_ALERT_2_G Short Intra H...H Contact  Hb1_A:18 ..Hd4_A:19 .       2.14 Ang.
                                                      x,y,z  =      1_555 Check
PLAT415_ALERT_2_G Short Inter D-H..H-X       H2_S:13  ..Hb1_A:39 .       2.13 Ang.
                                                      x,y,z  =      1_555 Check
PLAT416_ALERT_2_G Short Intra D-H..H-D       H0_A:38  ..H0A_A:39 .       2.08 Ang.
                                                      x,y,z  =      1_555 Check
PLAT417_ALERT_2_G Short Inter D-H..H-D       Hg_A:11  ..H1_S:17  .       2.11 Ang.
                                                      x,y,z  =      1_555 Check
PLAT417_ALERT_2_G Short Inter D-H..H-D       Hd2B_A:12..H2_S:19  .       1.73 Ang.
                                                      x,y,z  =      1_555 Check
PLAT417_ALERT_2_G Short Inter D-H..H-D       Hg1_A:21 ..H1_S:6   .       1.28 Ang.
                                                      x,y,z  =      1_555 Check
PLAT417_ALERT_2_G Short Inter D-H..H-D       H0_A:39  ..H2_S:13  .       2.13 Ang.
                                                      x,y,z  =      1_555 Check
PLAT417_ALERT_2_G Short Inter D-H..H-D       H2_S:12  ..H2_S:14  .       2.07 Ang.
                                                      x,y,z  =      1_555 Check
PLAT417_ALERT_2_G Short Inter D-H..H-D       H2_S:13  ..H0A_A:39 .       1.98 Ang.
                                                      x,y,z  =      1_555 Check
PLAT606_ALERT_4_G Solvent Accessible VOID(S) in Structure ........          ! Info
PLAT650_ALERT_4_G SWAT Instruction Used to Model Solvent Disorder           ! Report
PLAT720_ALERT_4_G Number of Unusual/Non-Standard Labels ..........       1025 Note
              N_A:1   H0A_A:1 H0B_A:1 H0C_A:1 Ca_A:1  Ha_A:1  Cb_A:1  Hb_A:1
              Cg2_A:1 Hg2A_A:1Hg2B_A:1Hg2C_A:1Og1_A:1 Hg1_A:1 C_A:1   O_A:1
              Cg2X_A:1Hg2D_A:1Hg2E_A:1Hg2F_A:1CaX_A:1 Ha1_A:1 Nx_A:1  H0D_A:1
              H0E_A:1 H0F_A:1 Cx_A:1  Ox_A:1  CbX_A:1 Hb1_A:1 Og1X_A:1Hg1A_A:1
              Nx_A:2  H0_A:2  Ox_A:2  Cx_A:2  Og1X_A:2Hg1_A:2 CbX_A:2 Hb_A:2
              CaX_A:2 Ha_A:2  Cg2X_A:2Hg2A_A:2Hg2B_A:2Hg2C_A:2C_A:2   O_A:2
              Cg2_A:2 Hg2D_A:2Hg2E_A:2Hg2F_A:2Og1_A:2 Hg1A_A:2Ca_A:2  Ha1_A:2
              Cb_A:2  Hb1_A:2 N_A:2   H0A_A:2 C_A:3   Sg_A:3  O_A:3   N_A:3
              H0_A:3  Ca_A:3  Ha_A:3  Cb_A:3  Hb1_A:3 Hb2_A:3 N_A:4   H0_A:4
              C_A:4   O_A:4   Sg_A:4  Ca_A:4  Ha_A:4  Cb_A:4  Hb1_A:4 Hb2_A:4
              N_A:5   O_A:5   C_A:5   Ca_A:5  Ha_A:5  Cb_A:5  Hb1_A:5 Hb2_A:5
              Cg_A:5  Hg1_A:5 Hg2_A:5 Cd_A:5  Hd1_A:5 Hd2_A:5 N_A:6   H0_A:6
              O_A:6   C_A:6   Ca_A:6  Ha_A:6  Og_A:6  Hg_A:6  Cb_A:6  Hb1_A:6
              Hb2_A:6 C_A:7   O_A:7   Ca_A:7  Ha_A:7  N_A:7   H0_A:7  Cg1_A:7
              Hg1A_A:7Hg1B_A:7Cg2_A:7 Hg2A_A:7Hg2B_A:7Hg2C_A:7Cd1_A:7 Hd1A_A:7
              Hd1B_A:7Hd1C_A:7Cb_A:7  Hb_A:7  CaX_A:7 Ha1_A:7 Cg1X_A:7Hg1C_A:7
              Hg1D_A:7Cg2X_A:7Hg2D_A:7Hg2E_A:7Hg2F_A:7Nx_A:7  H0A_A:7 Cx_A:7
              Ox_A:7  CbX_A:7 Hb1_A:7 Cd1X_A:7Hd1D_A:7Hd1E_A:7Hd1F_A:7O_A:8
              Cb_A:8  HbX_A:8 Ca_A:8  HaX_A:8 Cg1_A:8 Hg1A_A:8Hg1B_A:8Hg1C_A:8
              C_A:8   N_A:8   H0_A:8  Cg2_A:8 Hg2A_A:8Hg2B_A:8Hg2C_A:8CaX_A:8
              Ha1_A:8 CbX_A:8 Hb1_A:8 Cx_A:8  Ox_A:8  Cg2X_A:8Hg2D_A:8Hg2E_A:8
              Hg2F_A:8Nx_A:8  H0A_A:8 Cg1X_A:8Hg1D_A:8Hg1E_A:8Hg1F_A:8CaY_A:8
              Ha_A:8  CbY_A:8 Hb_A:8  Cg2Y_A:8Hg21_A:8Hg22_A:8Hg23_A:8Cg1Y_A:8
              Hg11_A:8Hg12_A:8Hg13_A:8Oy_A:8  Cy_A:8  Ny_A:8  C_A:9   O_A:9
              N_A:9   H0_A:9  Ca_A:9  Ha_A:9  Cb_A:9  Hb1_A:9 Hb2_A:9 Hb3_A:9
              N_A:10  H0_A:10 Ca_A:10 Ha_A:10 Cb_A:10 Hb1_A:10Hb2_A:10Cd_A:10
              Hd1_A:10Hd2_A:10Ne_A:10 He_A:10 Cg_A:10 Hg1_A:10Hg2_A:10Cz_A:10
              Nh1_A:10Hh1A_A:1Hh1B_A:1Nh2_A:10Hh2A_A:1Hh2B_A:1C_A:10  O_A:10
              Nx_A:10 H0A_A:10Cx_A:10 Ox_A:10 CaX_A:10Ha1_A:10CbX_A:10Hb3_A:10
              Hb4_A:10CdX_A:10Hd3_A:10Hd4_A:10CgX_A:10Hg3_A:10Hg4_A:10NeX_A:10
              He1_A:10CzX_A:10Nh1X_A:1Hh1C_A:1Hh1D_A:1Nh2X_A:1Hh2C_A:1Hh2D_A:1
```

N_A:11 H0_A:11 Ca_A:11 Ha_A:11 Cb_A:11 Hb1_A:11Hb2_A:11Og_A:11
Hg_A:11 C_A:11 O_A:11 N_A:12 H0_A:12 Ca_A:12 Ha_A:12 Nd2_A:12
Hd2A_A:1Hd2B_A:1Od1_A:12Cb_A:12 Hb1_A:12Hb2_A:12C_A:12 O_A:12
Cg_A:12 N_A:13 H0_A:13 O_A:13 C_A:13 Ca_A:13 Ha_A:13 Cb_A:13
Hb1_A:13Hb2_A:13Cg_A:13 Cd2_A:13Hd2_A:13Cd1_A:13Hd1_A:13Ce1_A:13
He1_A:13Ce2_A:13He2_A:13Cz_A:13 Hz_A:13 Nx_A:13 H0A_A:13CaX_A:13
Ha1_A:13CbX_A:13Hb3_A:13Hb4_A:13CgX_A:13Cd1X_A:1Hd1A_A:1Cd2X_A:1
Hd2A_A:1Ce1X_A:1He1A_A:1Ce2X_A:1He2A_A:1CzX_A:13Hz1_A:13Cx_A:13
Ox_A:13 N_A:14 H0_A:14 Ca_A:14 Ha_A:14 Cb_A:14 Hb1_A:14Hb2_A:14
Cg_A:14 Od1_A:14Nd2_A:14Hd2A_A:1Hd2B_A:1O_A:14 C_A:14 N_A:15
H0_A:15 Ca_A:15 Ha_A:15 Cb_A:15 Hb_A:15 Cg1_A:15Hg1A_A:1Hg1B_A:1
Hg1C_A:1Cg2_A:15Hg2A_A:1Hg2B_A:1Hg2C_A:1C_A:15 O_A:15 N_A:16
H0_A:16 Cb_A:16 Hb1_A:16Hb2_A:16Sg_A:16 Ca_A:16 Ha_A:16 O_A:16
C_A:16 N_A:17 H0_A:17 Ca_A:17 Ha_A:17 Cb_A:17 Hb1_A:17Hb2_A:17
Cg_A:17 Hg1_A:17Hg2_A:17Cd_A:17 Hd1_A:17Hd2_A:17Ne_A:17 He_A:17
Cz_A:17 Nh2_A:17Hh2A_A:1Hh2B_A:1Nh1_A:17Hh1A_A:1Hh1B_A:1C_A:17
O_A:17 N_A:18 H0_A:18 Ca_A:18 Ha_A:18 Cb_A:18 Hb1_A:18Hb2_A:18
Cd1_A:18Hd1A_A:1Hd1B_A:1Hd1C_A:1Cd2_A:18Hd2A_A:1Hd2B_A:1Hd2C_A:1
C_A:18 O_A:18 Cg_A:18 Hg_A:18 N_A:19 Ca_A:19 Ha_A:19 Cb_A:19
Hb1_A:19Hb2_A:19Cg_A:19 Hg1_A:19Hg2_A:19Cd_A:19 Hd1_A:19Hd2_A:19
C_A:19 O_A:19 Nx_A:19 CaX_A:19Ha1_A:19CbX_A:19Hb3_A:19Hb4_A:19
CgX_A:19Hg3_A:19Hg4_A:19CdX_A:19Hd3_A:19Hd4_A:19Ox_A:19 Cx_A:19
Ca_A:20 Ha1_A:20Ha2_A:20N_A:20 H0_A:20 C_A:20 O_A:20 N_A:21
H0_A:21 Ca_A:21 Ha_A:21 Cb_A:21 Hb_A:21 Og1_A:21Hg1_A:21Cg2_A:21
Hg2A_A:2Hg2B_A:2Hg2C_A:2C_A:21 O_A:21 N_A:22 Ca_A:22 Ha_A:22
Cb_A:22 Hb1_A:22Hb2_A:22Og_A:22 O_A:22 C_A:22 Nx_A:22 CaX_A:22
Ha1_A:22CbX_A:22Hb3_A:22Hb4_A:22CgX_A:22Hg1_A:22Hg2_A:22CdX_A:22
Hd1_A:22Hd2_A:22Cx_A:22 Ox_A:22 Ny_A:22 CaY_A:22Ha2_A:22CbY_A:22
Hb5_A:22Hb6_A:22OgY_A:22Oy_A:22 Cy_A:22 N_A:23 H0_A:23 Ca_A:23
Ha_A:23 Cb_A:23 Hb1_A:23Hb2_A:23Cd_A:23 Cg_A:23 Hg1_A:23Hg2_A:23
Oe1_A:23Oe2_A:23C_A:23 O_A:23 N_A:24 H0_A:24 Ca_A:24 Ha_A:24
Cb_A:24 Hb1_A:24Hb2_A:24Hb3_A:24C_A:24 O_A:24 N_A:25 H0_A:25
Ca_A:25 HaX_A:25Cb_A:25 HbX_A:25Cg1_A:25Hg1X_A:2Hg1Y_A:2Cg2_A:25
Hg2X_A:2Hg2Y_A:2Hg2Z_A:2Cd1_A:25Hd1X_A:2Hd1Y_A:2Hd1Z_A:2C_A:25
O_A:25 Nx_A:25 Hy_A:25 CaX_A:25HaY_A:25CbX_A:25Hb2_A:25Hb3_A:25
Cd1X_A:2Hd11_A:2Hd12_A:2Hd13_A:2Cd2X_A:2Hd21_A:2Hd22_A:2Hd23_A:2
CgX_A:25Hg_A:25 Cx_A:25 Ox_A:25 Ny_A:25 H_A:25 CaY_A:25Ha_A:25
CbY_A:25Hb_A:25 Cg1Y_A:2Hg1A_A:2Hg1B_A:2Cg2Y_A:2Hg2A_A:2Hg2B_A:2
Hg2C_A:2Cd1Y_A:2Hd1A_A:2Hd1B_A:2Hd1C_A:2Cy_A:25 Oy_A:25 N_A:26
H0_A:26 Ca_A:26 Ha_A:26 Sg_A:26 Cb_A:26 Hb1_A:26Hb2_A:26O_A:26
C_A:26 N_A:27 H0_A:27 Ca_A:27 Ha_A:27 Cb_A:27 Hb1_A:27Hb2_A:27
Hb3_A:27C_A:27 O_A:27 N_A:28 H0_A:28 Ca_A:28 Ha_A:28 Cb_A:28
Hb_A:28 Og1_A:28Hg1_A:28Cg2_A:28Hg2A_A:2Hg2B_A:2Hg2C_A:2C_A:28
O_A:28 N_A:29 H0_A:29 Ca_A:29 Ha_A:29 Cb_A:29 Hb1_A:29Hb2_A:29
Cg_A:29 Cd1_A:29Hd1_A:29Cd2_A:29Hd2_A:29Ce1_A:29He1_A:29Ce2_A:29
He2_A:29Cz_A:29 Oh_A:29 Hh_A:29 C_A:29 O_A:29 Nx_A:29 H0A_A:29
CaX_A:29Ha1_A:29CbX_A:29Hb3_A:29Hb4_A:29CgX_A:29Cd1X_A:2Hd1A_A:2
Cd2X_A:2Hd2A_A:2Ce1X_A:2He1A_A:2Ce2X_A:2He2A_A:2CzX_A:29OhX_A:29
Hh1_A:29Cx_A:29 Ox_A:29 Ny_A:29 H0B_A:29CaY_A:29Ha2_A:29CbY_A:29
Hb5_A:29Hb6_A:29CgY_A:29Cd1Y_A:2Hd1B_A:2Cd2Y_A:2Hd2B_A:2Ce2Y_A:2
He2B_A:2Ce1Y_A:2He1B_A:2CzY_A:29OhY_A:29Hh2_A:29Cy_A:29 Oy_A:29
N_A:30 H0_A:30 Ca_A:30 Ha_A:30 Cg2_A:30Hg2A_A:3Hg2B_A:3Hg2C_A:3
Og1_A:30Hg1_A:30Cb_A:30 Hb_A:30 C_A:30 O_A:30 N_A:31 H0_A:31
C_A:31 O_A:31 Ca_A:31 Ha1_A:31Ha2_A:31N_A:32 H0_A:32 Ca_A:32
Ha_A:32 Cb_A:32 Hb1_A:32Hb2_A:32Sg_A:32 C_A:32 O_A:32 N_A:33
H0_A:33 Ca_A:33 Ha_A:33 Cb_A:33 Hb_A:33 Cg2_A:33Hg2A_A:3Hg2B_A:3
Hg2C_A:3Cg1_A:33Hg1A_A:3Hg1B_A:3Cd1_A:33Hd1A_A:3Hd1B_A:3Hd1C_A:3

```
              C_A:33  O_A:33  N_A:34  H0A_A:34Ca_A:34 Ha1_A:34Cb_A:34 Hb1_A:34
              Cg1_A:34Hg1C_A:3Hg1D_A:3Cg2_A:34Hg2D_A:3Hg2E_A:3Hg2F_A:3C_A:34
              O_A:34  Cd1_A:34Hd1X_A:3Hd1Y_A:3Hd1Z_A:3Cd1Y_A:3Hd1D_A:3Hd1E_A:3
              Hd1F_A:3N_A:35  H0_A:35 Ca_A:35 Ha_A:35 Cb_A:35 Hb_A:35 Cg1_A:35
              Hg1A_A:3Hg1B_A:3Cg2_A:35Hg2A_A:3Hg2B_A:3Hg2C_A:3Cd1_A:35Hd1A_A:3
              Hd1B_A:3Hd1C_A:3C_A:35  O_A:35  N_A:36  Ca_A:36 Ha_A:36 Cb_A:36
              Hb1_A:36Hb2_A:36Cg_A:36 Hg1_A:36Hg2_A:36Cd_A:36 Hd1_A:36Hd2_A:36
              C_A:36  O_A:36  N_A:37  H0_A:37 Ca_A:37 Ha1_A:37Ha2_A:37C_A:37
              O_A:37  Nx_A:37 H0A_A:37CaX_A:37Ha3_A:37Ha4_A:37Cx_A:37 Ox_A:37
              N_A:38  H0_A:38 Ca_A:38 Ha_A:38 Cb_A:38 Hb1_A:38Hb2_A:38Hb3_A:38
              C_A:38  O_A:38  N_A:39  H0_A:39 Ca_A:39 Ha_A:39 Cb_A:39 Hb_A:39
              Og1_A:39Hg1_A:39Cg2_A:39Hg2A_A:3Hg2B_A:3Hg2C_A:3C_A:39  O_A:39
              Nx_A:39 H0A_A:39CaX_A:39Ha1_A:39CbX_A:39Hb1_A:39Cg2X_A:3Hg2D_A:3
              Hg2E_A:3Hg2F_A:3Og1X_A:3Hg1A_A:3Cx_A:39 Ox_A:39 N_A:40  H0_A:40
              Cb_A:40 Hb1_A:40Hb2_A:40Ca_A:40 Ha_A:40 Sg_A:40 C_A:40  O_A:40
              N_A:41  Ca_A:41 Ha_A:41 Cg_A:41 Hg1_A:41Hg2_A:41Cb_A:41 Hb1_A:41
              Hb2_A:41Cd_A:41 Hd1_A:41Hd2_A:41C_A:41  O_A:41  N_A:42  H0_A:42
              Ca_A:42 Ha1_A:42Ha2_A:42C_A:42  O_A:42  N_A:43  H0_A:43 Ca_A:43
              Ha_A:43 Cb_A:43 Hb1_A:43Hb2_A:43Cg_A:43 Od1_A:43Od2_A:43C_A:43
              O_A:43  Nx_A:43 H0A_A:43CaX_A:43Ha1_A:43CbX_A:43Hb3_A:43Hb4_A:43
              Od1X_A:4Od2X_A:4CgX_A:43Cx_A:43 Ox_A:43 N_A:44  H0_A:44 Ca_A:44
              Ha_A:44 Cb_A:44 Hb1_A:44Hb2_A:44Cg_A:44 Cd1_A:44Hd1_A:44Cd2_A:44
              Hd2_A:44Ce1_A:44He1_A:44Ce2_A:44He2_A:44Cz_A:44 Oh_A:44 Hh_A:44
              C_A:44  O_A:44  N_A:45  H0_A:45 Ca_A:45 Ha_A:45 Cb_A:45 Hb1_A:45
              Hb2_A:45Hb3_A:45C_A:45  O_A:45  N_A:46  H0_A:46 Ca_A:46 Ha_A:46
              Cb_A:46 Hb1_A:46Hb2_A:46Cg_A:46 Nd2_A:46Hd2A_A:4Hd2B_A:4Od1_A:46
              C_A:46  Ot1_A:46Ot2_A:46O_A:101 C1_A:101C2_A:101C1X_A:10Ox_A:101
              C2X_A:10C1_A:102C2_A:102O_A:102 H2_S:1  O_S:1   H1_S:1  H2_S:2
              O_S:2   H1_S:2  H2_S:3  O_S:3   H2_S:4  O_S:4   H1_S:4  H2_S:5
              O_S:5   H1_S:5  H2_S:6  O_S:6   H1_S:6  H2_S:7  O_S:7   O_S:8
              Ox_S:8  H2_S:9  O_S:9   H1_S:9  O_S:10  O_S:11  Ox_S:11 H2_S:12
              O_S:12  H1_S:12 H2_S:13 O_S:13  H1_S:13 H2_S:14 O_S:14  H1_S:14
              H2_S:15 O_S:15  H1_S:15 O_S:16  Ox_S:16 H2_S:17 O_S:17  H1_S:17
              O_S:18  Ox_S:18 H2_S:19 O_S:19  H1_S:19 H2_S:20 O_S:20  H1_S:20
              O_S:21  O_S:22  Ox_S:22 O_S:23  O_S:24  O_S:25  O_S:26  Ox_S:26
              O_S:27  O_S:29  Ox_S:29 O_S:30  Ox_S:30 O_S:31  O_S:32  Ox_S:32
              O_S:33  Ox_S:33 O_S:34  O_S:35  O_S:36  O_S:37  O_S:38  O_S:39
              O_S:40  O_S:41  O_S:42  O_S:44  Ox_S:44 O_S:45  O_S:46  Ox_S:46
              O_S:47  O_S:48  O_S:49  Ox_S:49 O_S:50  O_S:51  O_S:52  O_S:53
              Ox_S:53 O_S:56  O_S:57  Ox_S:57 Oy_S:57 O_S:69  O_S:71  O_S:73
              O_S:81
PLAT773_ALERT_2_G Check long C-C Bond in CIF: Cg1_A:34 --Cd1Y_A:34       1.85 Ang.
PLAT790_ALERT_4_G Centre of Gravity not Within Unit Cell: Resd.  #          2 Note
              C1.26 O5.04
PLAT790_ALERT_4_G Centre of Gravity not Within Unit Cell: Resd.  #          3 Note
              C2 O
PLAT790_ALERT_4_G Centre of Gravity not Within Unit Cell: Resd.  #          5 Note
              O1.34
PLAT790_ALERT_4_G Centre of Gravity not Within Unit Cell: Resd.  #          6 Note
              O
PLAT790_ALERT_4_G Centre of Gravity not Within Unit Cell: Resd.  #          7 Note
              H2 O
PLAT790_ALERT_4_G Centre of Gravity not Within Unit Cell: Resd.  #          8 Note
              H2 O
PLAT790_ALERT_4_G Centre of Gravity not Within Unit Cell: Resd.  #          9 Note
              H O
PLAT790_ALERT_4_G Centre of Gravity not Within Unit Cell: Resd.  #         10 Note
```

```
              H2 O
PLAT790_ALERT_4_G Centre of Gravity not Within Unit Cell: Resd.  #         11 Note
              H2 O
PLAT790_ALERT_4_G Centre of Gravity not Within Unit Cell: Resd.  #         12 Note
              H2 O
PLAT790_ALERT_4_G Centre of Gravity not Within Unit Cell: Resd.  #         13 Note
              H O
PLAT790_ALERT_4_G Centre of Gravity not Within Unit Cell: Resd.  #         14 Note
              O
PLAT790_ALERT_4_G Centre of Gravity not Within Unit Cell: Resd.  #         16 Note
              O
PLAT790_ALERT_4_G Centre of Gravity not Within Unit Cell: Resd.  #         17 Note
              O
PLAT790_ALERT_4_G Centre of Gravity not Within Unit Cell: Resd.  #         18 Note
              H2 O
PLAT790_ALERT_4_G Centre of Gravity not Within Unit Cell: Resd.  #         19 Note
              H2 O
PLAT790_ALERT_4_G Centre of Gravity not Within Unit Cell: Resd.  #         20 Note
              H2 O
PLAT790_ALERT_4_G Centre of Gravity not Within Unit Cell: Resd.  #         21 Note
              H2 O
PLAT790_ALERT_4_G Centre of Gravity not Within Unit Cell: Resd.  #         22 Note
              O
PLAT790_ALERT_4_G Centre of Gravity not Within Unit Cell: Resd.  #         23 Note
              H2 O
PLAT790_ALERT_4_G Centre of Gravity not Within Unit Cell: Resd.  #         24 Note
              O
PLAT790_ALERT_4_G Centre of Gravity not Within Unit Cell: Resd.  #         25 Note
              H2 O
PLAT790_ALERT_4_G Centre of Gravity not Within Unit Cell: Resd.  #         26 Note
              H2 O
PLAT790_ALERT_4_G Centre of Gravity not Within Unit Cell: Resd.  #         27 Note
              O
PLAT790_ALERT_4_G Centre of Gravity not Within Unit Cell: Resd.  #         28 Note
              O
PLAT790_ALERT_4_G Centre of Gravity not Within Unit Cell: Resd.  #         29 Note
              O
PLAT790_ALERT_4_G Centre of Gravity not Within Unit Cell: Resd.  #         30 Note
              O
PLAT790_ALERT_4_G Centre of Gravity not Within Unit Cell: Resd.  #         31 Note
              O
PLAT790_ALERT_4_G Centre of Gravity not Within Unit Cell: Resd.  #         32 Note
              O
PLAT790_ALERT_4_G Centre of Gravity not Within Unit Cell: Resd.  #         33 Note
              O
PLAT790_ALERT_4_G Centre of Gravity not Within Unit Cell: Resd.  #         34 Note
              O
PLAT790_ALERT_4_G Centre of Gravity not Within Unit Cell: Resd.  #         35 Note
              O
PLAT790_ALERT_4_G Centre of Gravity not Within Unit Cell: Resd.  #         36 Note
              O
PLAT790_ALERT_4_G Centre of Gravity not Within Unit Cell: Resd.  #         38 Note
              O
PLAT790_ALERT_4_G Centre of Gravity not Within Unit Cell: Resd.  #         39 Note
              O
PLAT790_ALERT_4_G Centre of Gravity not Within Unit Cell: Resd.  #         40 Note
              O
```

```
PLAT790_ALERT_4_G Centre of Gravity not Within Unit Cell: Resd.  #        41 Note
              O
PLAT790_ALERT_4_G Centre of Gravity not Within Unit Cell: Resd.  #        42 Note
              O
PLAT790_ALERT_4_G Centre of Gravity not Within Unit Cell: Resd.  #        43 Note
              O
PLAT790_ALERT_4_G Centre of Gravity not Within Unit Cell: Resd.  #        44 Note
              O
PLAT790_ALERT_4_G Centre of Gravity not Within Unit Cell: Resd.  #        45 Note
              O
PLAT790_ALERT_4_G Centre of Gravity not Within Unit Cell: Resd.  #        46 Note
              O
PLAT790_ALERT_4_G Centre of Gravity not Within Unit Cell: Resd.  #        47 Note
              O
PLAT790_ALERT_4_G Centre of Gravity not Within Unit Cell: Resd.  #        48 Note
              O
PLAT790_ALERT_4_G Centre of Gravity not Within Unit Cell: Resd.  #        49 Note
              O
PLAT790_ALERT_4_G Centre of Gravity not Within Unit Cell: Resd.  #        50 Note
              O
PLAT790_ALERT_4_G Centre of Gravity not Within Unit Cell: Resd.  #        51 Note
              O
PLAT790_ALERT_4_G Centre of Gravity not Within Unit Cell: Resd.  #        52 Note
              O
PLAT790_ALERT_4_G Centre of Gravity not Within Unit Cell: Resd.  #        53 Note
              O
PLAT790_ALERT_4_G Centre of Gravity not Within Unit Cell: Resd.  #        54 Note
              O
PLAT790_ALERT_4_G Centre of Gravity not Within Unit Cell: Resd.  #        55 Note
              O
PLAT790_ALERT_4_G Centre of Gravity not Within Unit Cell: Resd.  #        57 Note
              O
PLAT790_ALERT_4_G Centre of Gravity not Within Unit Cell: Resd.  #        58 Note
              O
PLAT790_ALERT_4_G Centre of Gravity not Within Unit Cell: Resd.  #        59 Note
              O
PLAT790_ALERT_4_G Centre of Gravity not Within Unit Cell: Resd.  #        60 Note
              O
PLAT790_ALERT_4_G Centre of Gravity not Within Unit Cell: Resd.  #        61 Note
              O
PLAT790_ALERT_4_G Centre of Gravity not Within Unit Cell: Resd.  #        62 Note
              O
PLAT811_ALERT_5_G No ADDSYM Analysis: Too Many Excluded Atoms ....          ! Info
PLAT860_ALERT_3_G Number of Least-Squares Restraints ............       14395 Note
PLAT868_ALERT_4_G ALERTS Due to the Use of _smtbx_masks Suppressed          ! Info
PLAT899_ALERT_4_G SHELXL2018 is Deprecated and Succeeded by SHELXL     2019/3 Note
PLAT912_ALERT_4_G Missing # of FCF Reflections Above STh/L=  0.600      6861 Note
PLAT933_ALERT_2_G Number of HKL-OMIT Records in Embedded .res File         2 Note
                1  1  1,   0  1  2,
PLAT965_ALERT_2_G The SHELXL WEIGHT Optimisation has not Converged     Please Check
PLAT969_ALERT_5_G The 'Henn et al.' R-Factor-gap value ..........      10.19 Note
              Predicted wR2: Based on SigI**2  1.67 or SHELX Weight 14.90
PLAT978_ALERT_2_G Number C-C Bonds with Positive Residual Density.         0 Info
PLAT992_ALERT_5_G Repd & Actual _reflns_number_gt Values Differ by         2 Check
```

---

109 **ALERT level A** = Most likely a serious problem - resolve or explain

```
 72 ALERT level B = A potentially serious problem, consider carefully
790 ALERT level C = Check. Ensure it is not caused by an omission or oversight
838 ALERT level G = General information/check it is not something unexpected

112 ALERT type 1 CIF construction/syntax error, inconsistent or missing data
373 ALERT type 2 Indicator that the structure model may be wrong or deficient
558 ALERT type 3 Indicator that the structure quality may be low
762 ALERT type 4 Improvement, methodology, query or suggestion
  4 ALERT type 5 Informative message, check
```

It is advisable to attempt to resolve as many as possible of the alerts in all categories. Often the minor alerts point to easily fixed oversights, errors and omissions in your CIF or refinement strategy, so attention to these fine details can be worthwhile. In order to resolve some of the more serious problems it may be necessary to carry out additional measurements or structure refinements. However, the purpose of your study may justify the reported deviations and the more serious of these should normally be commented upon in the discussion or experimental section of a paper or in the "special_details" fields of the CIF. checkCIF was carefully designed to identify outliers and unusual parameters, but every test has its limitations and alerts that are not important in a particular case may appear. Conversely, the absence of alerts does not guarantee there are no aspects of the results needing attention. It is up to the individual to critically assess their own results and, if necessary, seek expert advice.

Publication of your CIF in IUCr journals

A basic structural check has been run on your CIF. These basic checks will be run on all CIFs submitted for publication in IUCr journals (*Acta Crystallographica*, *Journal of Applied Crystallography*, *Journal of Synchrotron Radiation*); however, if you intend to submit to *Acta Crystallographica Section C* or *E* or *IUCrData*, you should make sure that full publication checks are run on the final version of your CIF prior to submission.

Publication of your CIF in other journals

Please refer to the *Notes for Authors* of the relevant journal for any special instructions relating to CIF submission.

PLATON version of 06/01/2024; check.def file version of 05/01/2024

Datablock crambin_rt - ellipsoid plot

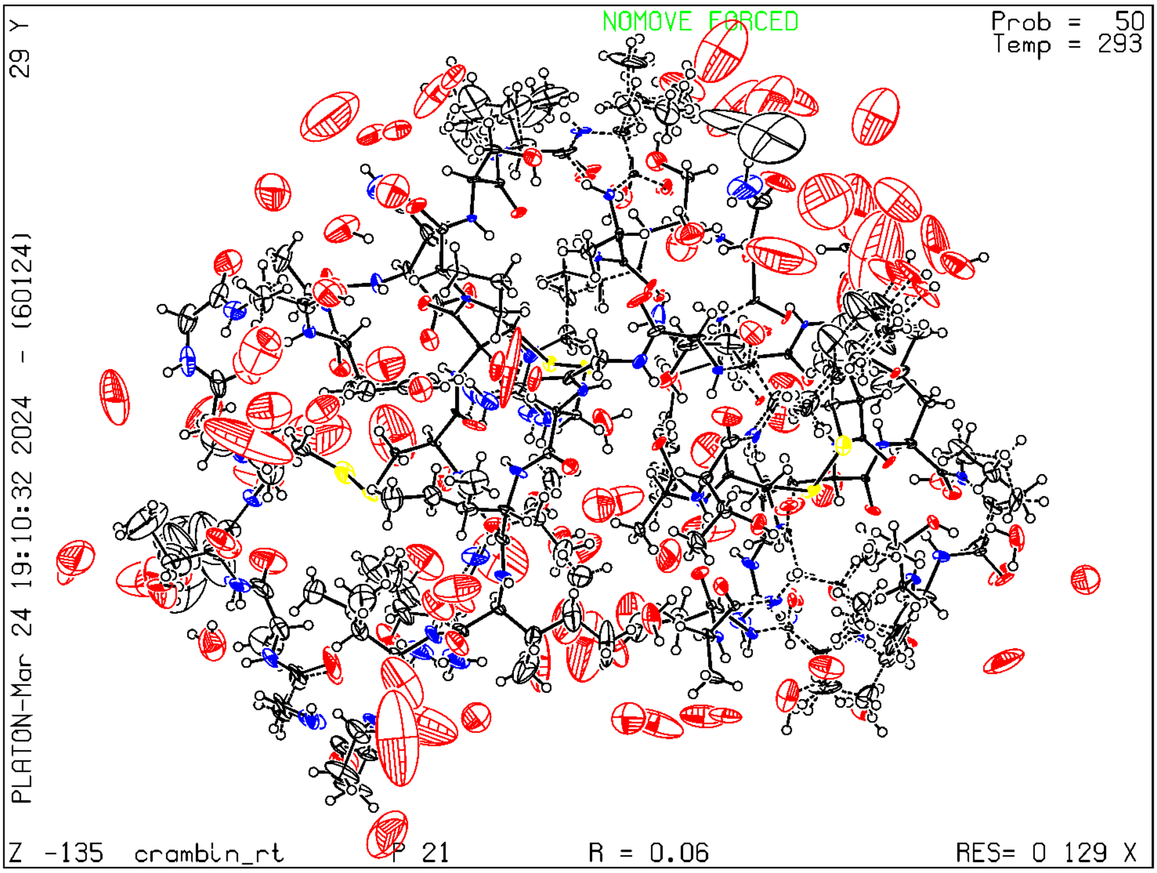

Supplement: Supplementary file 10 [file m-11-00649-sup10.pdf]
