# Supplementary material for: Solvent organization in the ultrahigh-resolution crystal structure of crambin at room temperature
Source: IUCrJ. 2024 Aug 27;11(Pt 5):649–63. doi: 10.1107/S2052252524007784 (PMC11364037; doi:10.1107/S2052252524007784)

IUCrJ

Volume 11 (2024)

Supporting information for article:

Solvent organization in the ultrahigh-resolution crystal structure crambin at room temperature

Julian C.-H. Chen, Miroslaw Gilski, Changsoo Chang, Dominika Borek, Gerd Rosenbaum, Alex Lavens, Zbyszek Otwinowski, Maciej Kubicki, Zbigniew Dauter, Mariusz Jaskolski and Andrzej Joachimiak

Table S1 List of protein structures in PDB with diffraction data acquired at temperatures above 273 K.

Custom Report

Identifier	Structure Data		
**Entry ID**	**Collection Temperature**	**PDB ID**	**High Resolution Limit**
2PWA	277	2PWA	0.83
1P9G	283	1P9G	0.84
3U7T	298	3U7T	0.85
1O56	298	1O56	0.9
7PSY	293	7PSY	0.9
1RB9	293	1RB9	0.92
4AR6	295	4AR6	0.92
5MNH	295	5MNH	0.93
5MOQ	295	5MOQ	0.93, 1.502
6NIZ	295.15	6NIZ	0.93
5MNB	295	5MNB	0.939
5MON	295	5MON	0.939, 1.42
4LZT	295	4LZT	0.95
1AHO	287	1AHO	0.96
5MNO	295	5MNO	0.96
5MOS	295	5MOS	0.96, 1.5
1IC6	295	1IC6	0.98
5CE4	293	5CE4	0.98
5MNM	295	5MNM	0.98
5MOR	295	5MOR	0.98, 1.49
8RC7	293	8RC7	0.98
1C58	277	1C58	0.99
2BF9	293	2BF9	0.99
3X2P	298	3X2P	1.518, 0.99
5MNF	295	5MNF	0.99
5MOP	295	5MOP	0.99, 1.45
1C7K	277	1C7K	1
1LWB	297	1LWB	1.05

1 to 28 of 28 **Structures**

Table S2 List of multiple-conformations residues and their dihedral angles.

	A: Φ	B: Φ	C: Φ	A: Ψ	B: Ψ	C: Ψ	A: X1	B: X1	C: X1	A: X2	B: X2	C: X2	A: X3	B: X3	A: X4	B: X4
Thr1	-109.1	-91.3		142.7	167.1		-63.0	-166.0								
Thr2	-132.9	-132.2		145.4	133.4		59.3	-58.0								
Iie7	-52.0	-69.5		-41.1	-52.1		-68.2	-71.7		175.1	-64.7					
Val8	-64.5	-60.0	-57.8	-47.8	-30.5	-29.1	164.3	73.0	160.5							
Arg10	-61.1	-61.8		-49.1	-40.7		176.9	176.1		64.8	67.0		64.5	69.9	-177.7	169.2
Phe13	-65.5	-65.2		-42.6	-45.5		-171.3	179.8		-90.9	-87.1					
Pro19	103.3	105.4		-4.0	-12.0		24.5	-17.4		-31.1	32.4		24.8	-34.4		
Pro/Ser 22	-54.9	-53.1	-57.0	147.8	144.8	142.5	107.0	-23.8	108.1		30.9					
Ile/Leu 25	-66.8	-62.3	-65.5	-42.1	-39.7	-39.7	-67.9	110.5	-68.4	-173.8	143.2	-72.0				
Tyr 29	-108.9	-101.5	-107.6	-41.3	-41.9	-31.3	174.8	-169.8	-173.6	62.8	67.9	45.5				
ILE 34	-122.0			127.1			-55.3			171.2	-43.6					
Gly37	-124.3	-113.4		-154.8	-172.3											
Thr39	-70.6	-78.1		96.2	113.6		-50.3	-55.2								
Asp43	-131.8	-128.7		0.1	0.0		48.6	61.9		-15.6	-21.9					

Figure S1 *2mF$_o$-DF$_c$* electron density map calculated around residues Val8 and Tyr29. (a) Val8 has triple conformation clearly visible in electron density at 1.3σ. Occupancies of conformers A, B and C refined to 45%, 40%, 15%, respectively. (b) Tyr29 has triple conformation clearly visible in electron density at 1.0σ. Occupancies of conformers A, B and C refined to 41%, 23%, 36%, respectively.

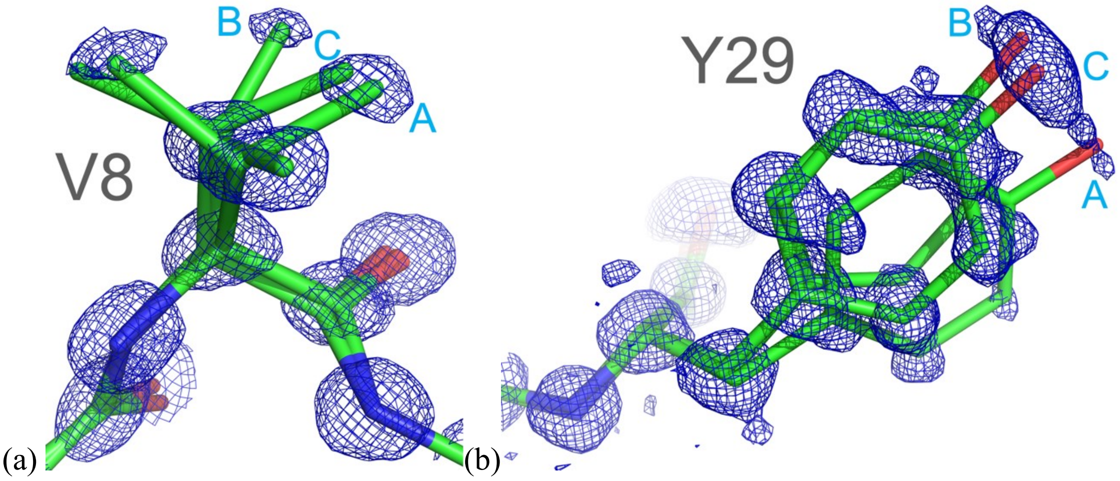

(a) (b)

Figure S2 Uncertainties (s.u.) of the crambin atomic coordinates calculated using the full-matrix least-squares refinement, plotted against their U_{eq} ADP values. U_{eq} is defined as 1/3 of the trace of the orthogonalized U_{ij} tensor.

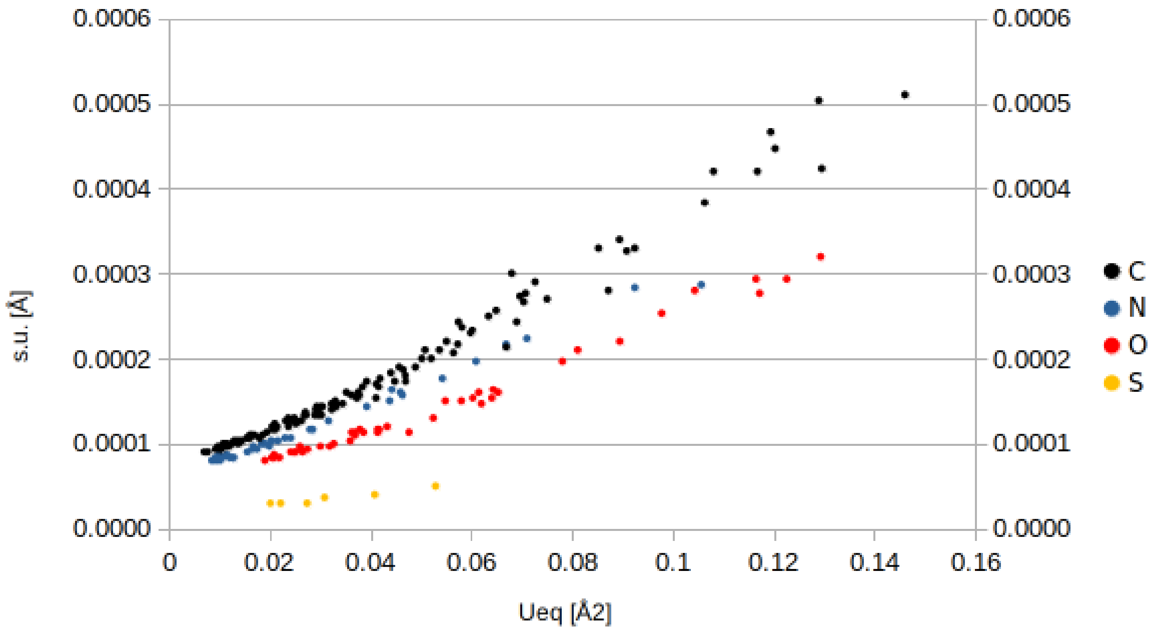

Figure S3 Stereo view of the interactions of Tyr29 side chain at the interface of four molecules of crambin. Tyr29 interacts with three adjacent symmetry-related molecules. The crambin molecule in ASU is in silver and is shown in stick representation. The three symmetry-related molecules are labeled in green, blue, and magenta, with interacting residues indicated. The sequence heterogeneity of PL and SI isoforms is indicated in blue. Residues Tyr29 and Val8 adopt multiple conformations (please see Supplemental Fig. S1), and the aromatic ring of Tyr29 shifts depending on the identity of the adjacent protein sequence (blue). Waters with hydrogen atoms are in stick representation and other water molecules are shown as spheres.

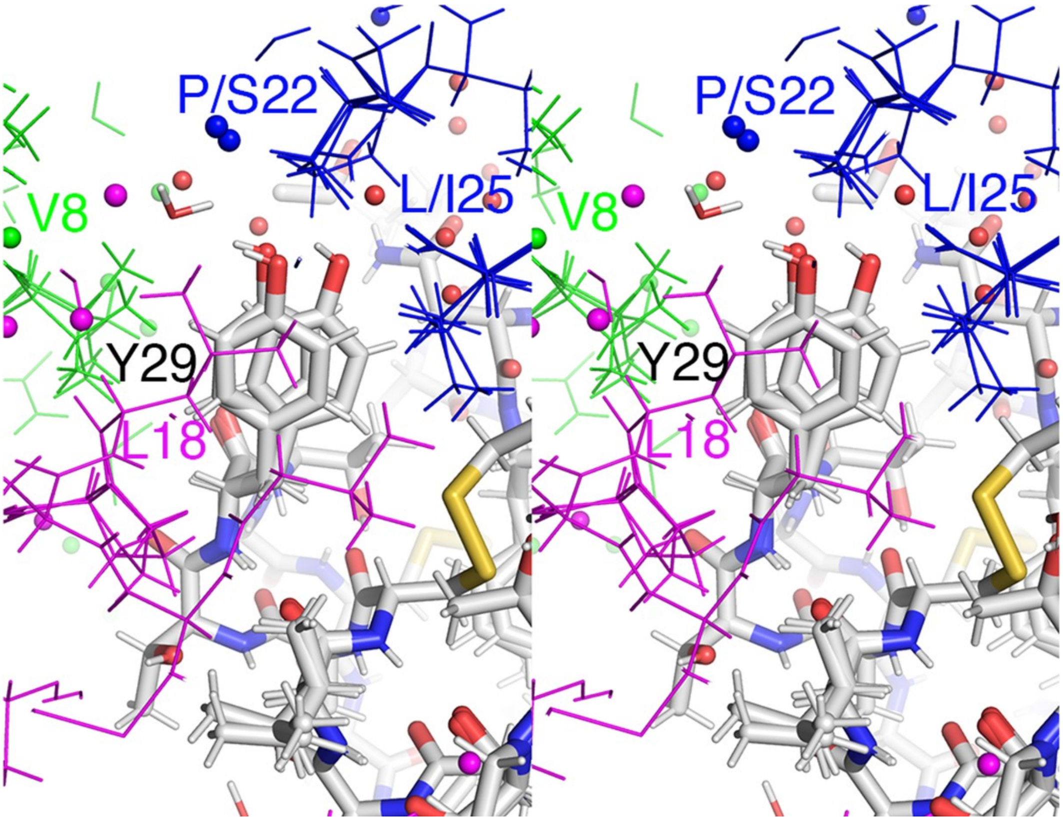

Figure S4 The „coordination" polyhedron of a single crambin molecule in the crystal. Each point represents the center of gravity of the crambin molecule. There are 12 neighboring molecules surrounding the central crambin molecule. The coordination polyhedron is a distorted (elongated) cuboctahedron that is analogous to the cubic closest packing of spheres. Red numbers correspond to distances between centers of neighboring molecules in Å.

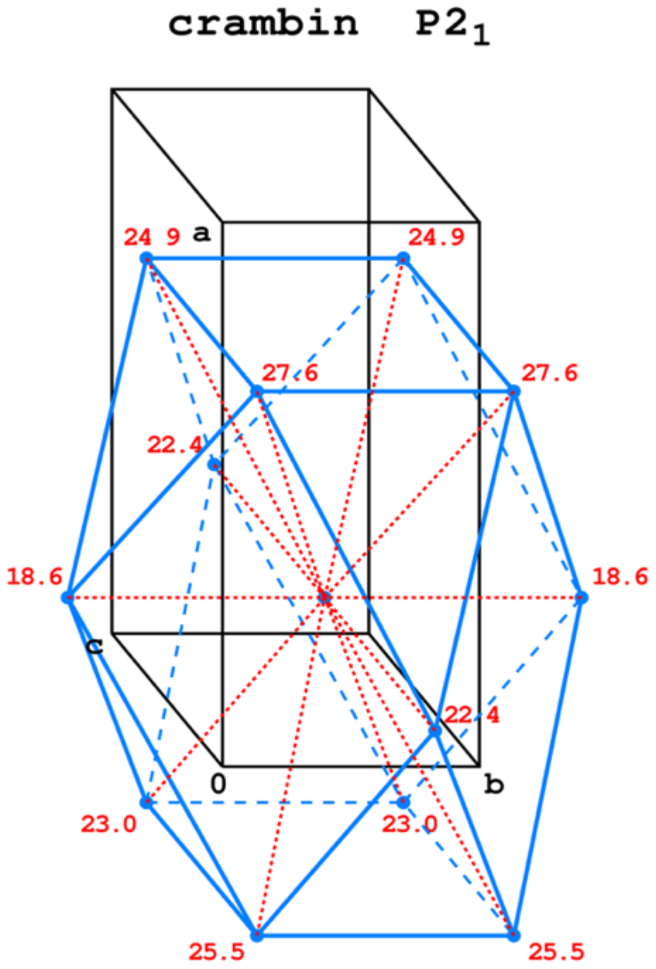

Supplement: Supplementary file 12 [file m-11-00649-sup12.pdf]
